# Publisher Correction: Stapling strategy for slowing helicity interconversion of α-helical peptides and isolating chiral auxiliary-free one-handed forms

**DOI:** 10.1038/s41467-024-46219-6

**Published:** 2024-02-28

**Authors:** Naoki Ousaka, Mark J. MacLachlan, Shigehisa Akine

**Affiliations:** 1 Nano Life Science Institute (WPI-NanoLSI), Kanazawa University, Kakuma-machi, Kanazawa, 920-1192 Japan; 2 Department of Chemistry, University of British Columbia, 2036 Main Mall, Vancouver, BC V6T 1Z1 Canada; 3 Quantum Matter Institute, University of British Columbia, 2355 East Mall, Vancouver, BC V6T 1Z4 Canada; 4 Graduate School of Natural Science and Technology, Kanazawa University, Kanazawa, 920-1192 Japan

**Keywords:** Stereochemistry, Supramolecular chemistry, Peptides

Correction to: *Nature Communications* 10.1038/s41467-023-42493-y, published online 26 October 2023

The original version of this article contained an error in Ref. 49, which was incorrectly given as: *J. Chem. Soc., Perkin Trans*. **2**, 1651–1657 (1998). The correct form of Ref. 49 is: *J. Chem. Soc. Perkin Trans*. *2*, 1651–1657 (1998). This has been corrected in the PDF and HTML versions of the Article.

