# Supplementary material for: Stapling strategy for slowing helicity interconversion of α-helical peptides and isolating chiral auxiliary-free one-handed forms
Source: Nat Commun. 2023 Oct 26;14:6834. doi: 10.1038/s41467-023-42493-y (PMC10603036; doi:10.1038/s41467-023-42493-y)
Supplement: Supplementary file 1 — Supplementary Information [file 41467_2023_42493_MOESM1_ESM.pdf]

Supplementary information:

Stapling strategy for slowing helicity interconversion of α-helical peptides and isolating chiral auxiliary-free one-handed forms

Naoki Ousaka,[1]* Mark J. MacLachlan[1,2,3]* and Shigehisa Akine[1,4]*

[1]Nano Life Science Institute (WPI-NanoLSI), Kanazawa University, Kakuma-machi, Kanazawa 920-1192, Japan. [2]Department of Chemistry, University of British Columbia, 2036 Main Mall, Vancouver, BC, V6T 1Z1, Canada. [3]Quantum Matter Institute, University of British Columbia, 2355 East Mall, Vancouver, BC, V6T 1Z4, Canada. [4]Graduate School of Natural Science and Technology, Kanazawa University, Kanazawa 920-1192, Japan.

E-mail: ousaka@staff.kanazawa-u.ac.jp (N.O.); mmaclach@chem.ubc.ca (M.J.M.); akine@se.kanazawa-u.ac.jp (S.A.)

Table of Contents

1. Materials and Instruments S2
2. Synthetic Procedures S3
3. Molecular Design Strategy for Singly and Doubly Crosslinked (Stapled) α-Helical Peptides S27
4. ESI-TOF Mass Spectra and HPLC Chromatograms of **c1-Val-O*t*Bu**, **c1-Val-OH**, **dc2-Val-OH and dc2-Aib-OMe** S28
5. Theoretical Studies on the Structures of Stapled Peptides **c1-Val-OH** and **dc2-Aib-OMe** S30
6. X-ray Crystallographic Analysis of **dc2-Aib-OH** S37
7. Conformational Analysis of **c1-Val-OH** by ^{1}H-^{1}H NOESY and VT-NMR Spectroscopy S43
8. Conformational Analysis of **dc2-Aib-OMe** by ^{1}H-^{1}H NOESY and VT-NMR Spectroscopy S48
9. Determination of the Molar Ratios of (*P*)- and (*M*)- **c1-Val-O*t*Bu** and (*P*)- and (*M*)- **c1-Val-OH** by ^{1}H NMR and CD Spectroscopies S52
10. Kinetic and Thermodynamic Analyses of Acid/Base-Triggered Reversible Helicity Inversion and Solvent-Induced Helicity Inversion of **c1-Val-OH** S53
11. Determination of the Association Constants of **c1-Val-OH** with Various Organic Bases S58
12. Kinetic and Thermodynamic Analyses of the Transformation of the Kinetically-Trapped (*M*)-Rich **dc2-Val-OH** into the Thermodynamically-Stable (*P*)-Rich **dc2-Val-OH** S61
13. Kinetic and Thermodynamic Analyses of the Racemization of (*M*)-**dc2-Aib-OMe** S66
14. ^{1}H and ^{13}C NMR Spectra S68
15. Supporting References S95

1. Materials and Instruments

Instruments

NMR spectra were measured using a Bruker Ascend 400 spectrometer operating at 400 MHz for ^{1}H and 100 MHz for ^{13}C using tetramethylsilane (TMS) or a residual solvent peak as the internal standard. Absorption and CD spectra were measured in a 0.1-cm or 1.0-cm quartz cell using a JASCO V-750 spectrophotometer and a JASCO J-1500 spectropolarimeter, respectively. The temperature was controlled with a JASCO PTC-510 apparatus. Electrospray ionization (ESI) time-of-flight (TOF) mass spectra were recorded using a Bruker Daltonics micrOTOF II spectrometer. Size exclusion chromatography (SEC) fractionations were performed using an LC-9201 liquid chromatograph (Japan Analytical Industry, Tokyo, Japan) equipped with an SEC column (JALGEL-2HR-40 (4 (i.d.) × 60 cm)) and UV-visible (254 nm, JASCO UV-2075 Plus) and RI (JAI RI-50s) detectors; $CHCl_3$/MeOH (9/1, v/v) was used as the eluent at a flow rate of 9.0 mL/min. The high-performance liquid chromatography (HPLC) analyses and separations were performed on a JASCO PU-4180 liquid chromatograph equipped with UV-visible (JASCO MD-4010) and CD (JASCO CD-4095) detectors using a CHIRALPAK IB N-5 column (0.46 (i.d.) × 25 cm or 1.0 (i.d.) × 25 cm, Daicel, Osaka, Japan) or a COSMOSIL $5C_{18}$-MS-II (0.46 (i.d.) × 25 cm, Nacalai Tesque, Kyoto, Japan).

Materials

All starting materials were purchased from commercial suppliers and were used without further purification unless otherwise noted. 1,1,2,2-Tetrachloroethane was distilled over CaH_2 under reduced pressure and stored over 3 Å molecular sieves. *n*-Butanol was distilled over 3 Å molecular sieves and stored over 3 Å molecular sieves. Silica gel (SiO_2) and aminopropyl-modified silica gel (NH-SiO_2) for the flash chromatography were purchased from Kanto Chemical Co., Inc. and Fuji Silysia Chemical Ltd. (Kasugai, Japan) or Fujifilm Wako Pure Chemical Co., respectively. HCl·H-Aib-OMe[1], Z-Api(Boc)-OH[2], Z-Ac_6c-O^{t}Bu[3], and Z-(Ac_6c)$_2$-OH[3] were synthesized according to the reported methods.

2. Synthetic Procedures

Abbreviations of chemicals and substituents:

Z: benzyloxycarbonyl,

Boc: *tert*-butoxycarbonyl,

Aib: α-aminoisobutyric acid,

Ac_6c: 1-aminocyclohexanecarboxylic acid,

Api: 4-aminopiperidine-4-carboxylic acid,

dppf: 1,1'-bis(diphenylphosphino)ferrocene,

Pin_2B_2: bis(pinacolate)diboron,

HOSu: *N*-hydroxysuccinimide,

COMU: 1-[(1-(cyano-2-ethoxy-2-oxoethylideneaminooxy) dimethylaminomorpholino)] uronium hexafluorophosphate,

EDC·HCl: 1-ethyl-3-(3-dimethylaminopropyl)-carbodiimide hydrochloride,

DIPEA: *N*,*N*-diisopropylethylamine,

TFA: trifluoroacetic acid,

TBD: 1,5,7-triazabicyclo[4.4.0]dec-5-ene,

DBU: 1,8-diazabicyclo[5.4.0]undec-7-ene.

General Procedures for the Peptide Syntheses.

Boc- or -O^tBu and Z-protecting groups were removed by treatment with TFA, formic acid, or 4N HCl in 1,4-dioxane and 10% Pd-C/H_2 in MeOH or MeOH/CH_2Cl_2, respectively. The resulting N- or C-deprotected peptides were used without further purification unless otherwise noted. Peptide coupling reactions were carried out by a COMU method[4,5].

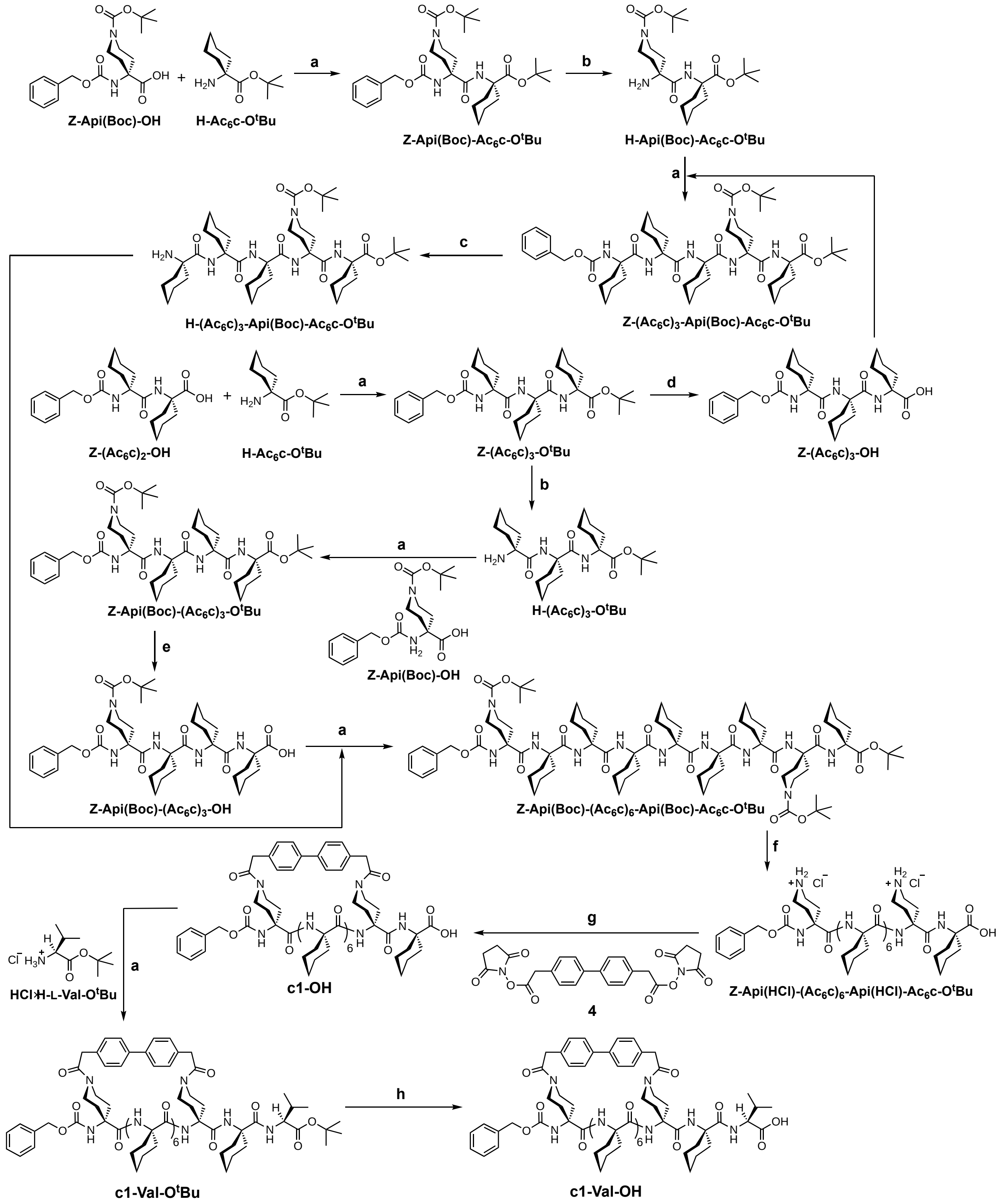

Supplementary Figure 1 | Synthetic route to c1-Val-O^{t}Bu and c1-Val-OH. Syntheses of the singly stapled peptides **c1-Val-O^{t}Bu** and **c1-Val-OH**. Reagents and conditions: (a) COMU, DIPEA, CH_2Cl_2, 0 °C to r.t.; (b) 10% Pd-C/H_2, CH_2Cl_2/MeOH, r.t.; (c) 10% Pd-C/H_2, MeOH; (d) CH_2Cl_2/TFA, r.t.; (e) (i) CH_2Cl_2/TFA, r.t.; (ii) Boc_2O, DIPEA, r.t.; (f) CH_2Cl_2/4N HCl in 1,4-dioxane, r.t.; (g) DIPEA, DMF, r.t.; (h) CH_2Cl_2/HCO_2H, r.t.

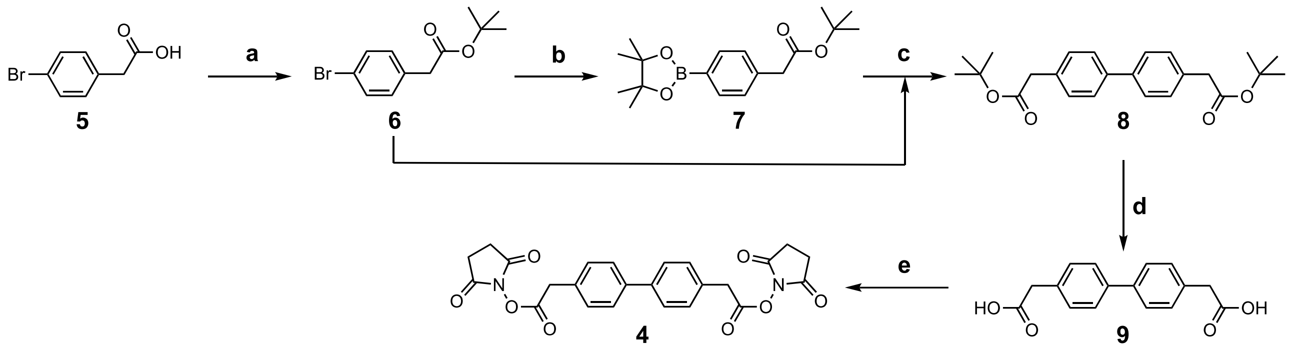

Supplementary Figure 2 | Synthetic route to 4. Synthesis of the cross-linking reagent **4**. Reagents and conditions: (a) isobutene, CH_2Cl_2, conc. H_2SO_4, r.t.; (b) Pd(dppf)Cl_2·CH_2Cl_2, Pin_2B_2, KOAc, 1,4-dioxane, 75 ºC; (c) K_3PO_4, $Pd(PPh_3)_4$, 1,4-dioxane, reflux; (d) CH_2Cl_2/TFA, r.t.; (e) HOSu, EDC·HCl, CH_2Cl_2, 0 ºC to r.t.

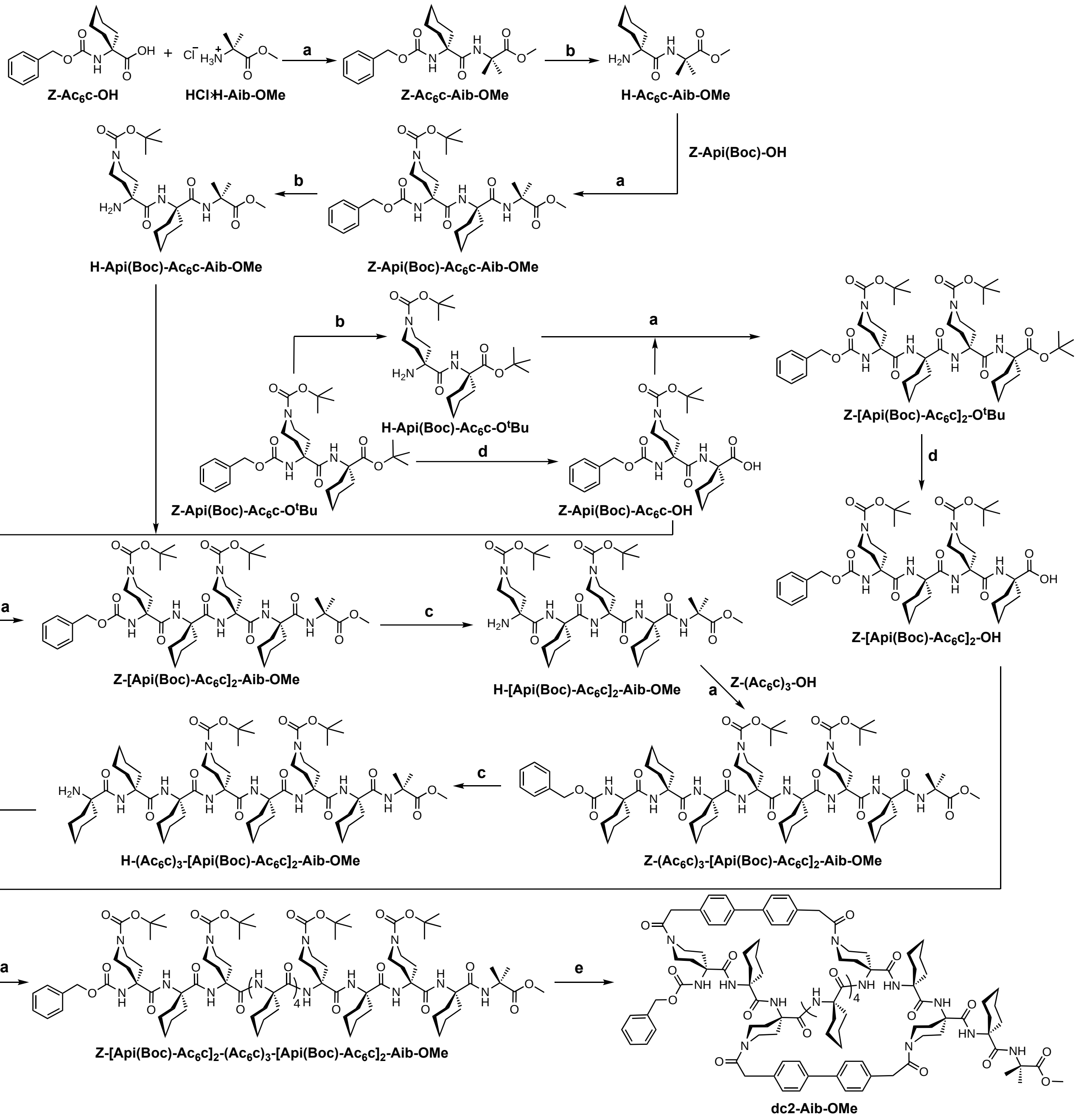

Supplementary Figure 3 | Synthetic route to dc2-Aib-OMe. Synthesis of the doubly stapled achiral peptide **dc2-Aib-OMe**. Reagents and conditions: (a) COMU, DIPEA, CH_2Cl_2, 0 °C to r.t.; (b) 10% Pd-C/H_2, MeOH, r.t.; (c) 10% Pd-C/H_2, CH_2Cl_2/MeOH, r.t.; (d) (i) CH_2Cl_2/TFA, r.t.; (ii) Boc_2O, DIPEA, r.t.; (e) (i) CH_2Cl_2/MeOH/4N HCl in 1,4-dioxane, r.t.; (ii) **4**, DIPEA, DMF, r.t.

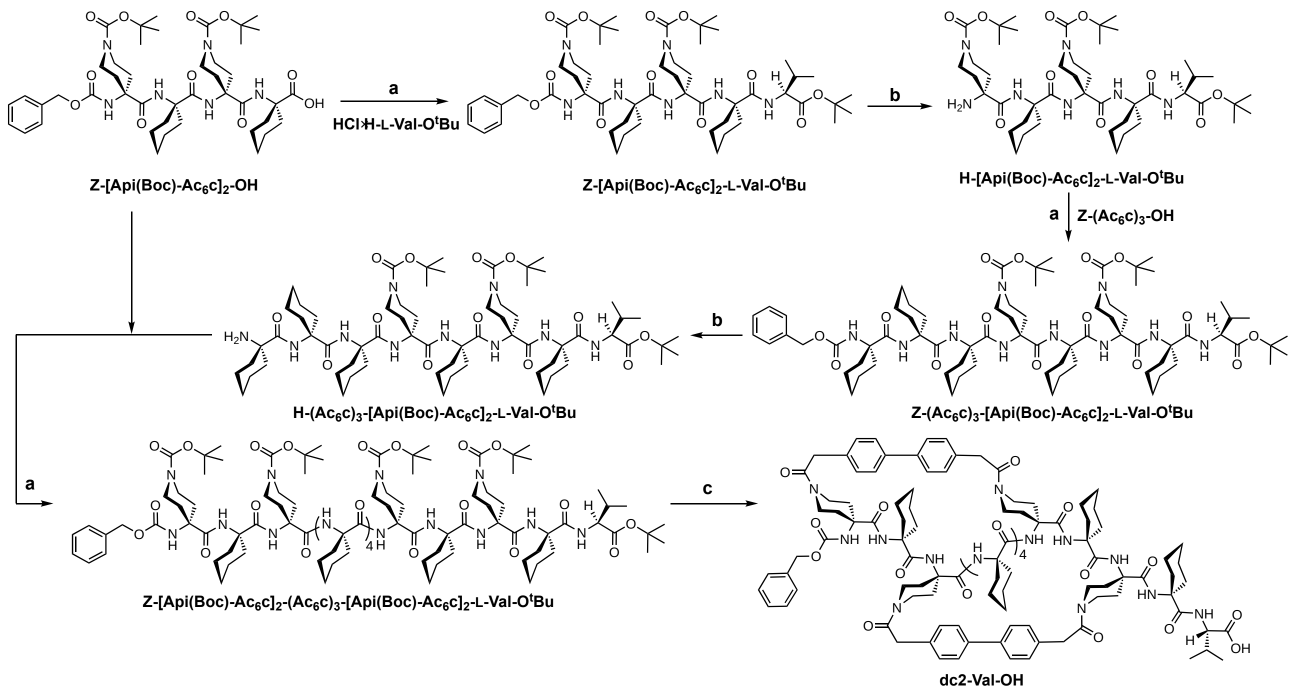

Supplementary Figure 4 | Synthetic route to dc2-Val-OH. Synthesis of the doubly stapled peptide **dc2-Val-OH**. Reagents and conditions: (a) COMU, DIPEA, CH_2Cl_2, 0 °C to r.t.; (b) 10% Pd-C/H_2, CH_2Cl_2/MeOH, r.t.; (c) (i) CH_2Cl_2/HCO_2H, r.t. then CH_2Cl_2/MeOH/4N HCl in 1,4-dioxane, r.t.; (ii) **4**, DIPEA, CH_2Cl_2/DMF, r.t.

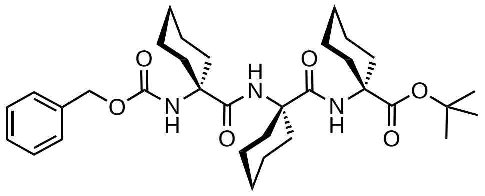

Z-(Ac$_6$c)$_3$-O^{t}Bu: To a solution of Z-(Ac$_6$c)$_2$-OH (7.01 g, 17.4 mmol) and H-Ac$_6$c-O^{t}Bu (3.47 g, 17.4 mmol), which had been obtained by treatment of Z-Ac$_6$c-O^{t}Bu with 10% Pd-C/H_2 in MeOH, in dry CH_2Cl_2 (20 mL) was added DIPEA (6.06 mL, 34.8 mmol) at room temperature. To this was added COMU (7.46 g, 17.4 mmol) at 0 ºC, and the mixture was stirred at 0 ºC for 1 h and further at room temperature for 20.5 h under N_2. The solvent was then evaporated to dryness under reduced pressure. The residue was dissolved in EtOAc, and the solution was washed with 1N aqueous HCl, 5% aqueous $NaHCO_3$, and brine, dried over $MgSO_4$, filtered, and concentrated under reduced pressure. The residue was purified by recrystallization from EtOAc/*n*-hexane (ca. 1/3, v/v), affording title compound Z-(Ac$_6$c)$_3$-O^{t}Bu as a white solid (7.42 g, 73%). ^{1}H NMR (400 MHz, $CDCl_3$, 25 ºC): δ 7.38–7.29 (m, 5H), 7.24 (s, 1H), 6.39 (s, 1H), 5.17 (s, 1H), 5.10 (s, 2H), 2.03–1.22 (m, partially overlapping with H_2O signal). ^{13}C NMR (100 MHz, $CDCl_3$, 25 ºC): δ 173.56, 173.48, 173.02, 155.35, 136.08, 128.76, 128.56, 128.23, 80.02, 67.35, 59.94, 58.97, 32.26, 31.98, 31.80, 28.01, 25.57, 25.23, 25.06, 21.53, 21.37. HRMS (ESI-TOF+): *m/z* calcd for (M+Na$^+$), 606.3514; found, 606.3546.

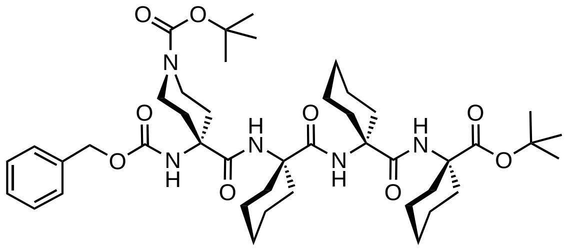

Z-Api(Boc)-(Ac$_6$c)$_3$-O^{t}Bu: To a solution of Z-Api(Boc)-OH (3.22 g, 8.50 mmol) and H-(Ac$_6$c)$_3$-O^{t}Bu (3.82 g, 8.50 mmol), which had been obtained by treatment of Z-(Ac$_6$c)$_3$-O^{t}Bu with 10% Pd-C/H_2 in MeOH/CH_2Cl_2 (2/1, v/v), in dry CH_2Cl_2 (12 mL) was added DIPEA (2.96 mL, 17.0 mmol) at room temperature. To this was added COMU (3.64 g, 8.50 mmol) at 0 ºC, and the mixture was stirred at 0 ºC for 1 h and further at room temperature for 16 h under N_2. The solution was diluted with EtOAc, and the mixture solution was washed with 1N aqueous HCl, 5% aqueous $NaHCO_3$, and brine, dried over $MgSO_4$, filtered, and concentrated under reduced pressure. The residue was purified by column chromatography (SiO_2, EtOAc/CH_2Cl_2 (4/6, v/v)), affording title compound Z-Api(Boc)-(Ac$_6$c)$_3$-O^{t}Bu as a slightly reddish solid (6.10 g, 89%). ^{1}H NMR (400 MHz, $CDCl_3$, 25 ºC): δ 7.39–7.31 (m, 5H), 7.19 (s, 1H), 6.65 (s, 1H), 6.53 (s, 1H), 5.27 (s, 1H), 5.12 (s, 2H), 3.88–3.85 (m, 2H), 3.14–3.08 (m, 2H), 2.25–2.04 (m, 6H), 1.91–1.52 (m, partially overlapping with H_2O signal), 1.45 (s,

9H), 1.41 (s, 9H), 1.29–1.15 (m, 8H). ^{13}C NMR (100 MHz, $CDCl_3$, 25 ºC): δ 173.91, 173.75, 172.82, 172.61, 155.72, 154.63, 80.06, 79.80, 67.16, 60.33, 59.84, 59.20, 58.33, 39.41, 32.32, 32.00, 31.73, 31.48, 28.45, 28.00, 25.75, 25.41. 25.11, 21.62, 21.55. HRMS (ESI-TOF+): *m/z* calcd for ($M+Na^+$), 832.4831; found, 832.4881.

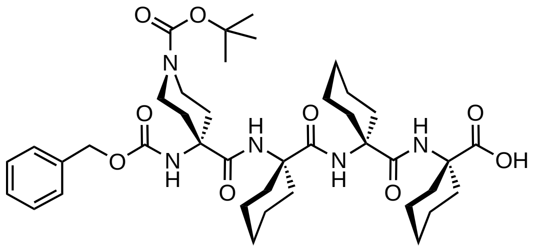

Z-Api(Boc)-(Ac_6c)$_3$-OH: Z-Api(Boc)-(Ac_6c)$_3$-O^{t}Bu (5.50 g, 6.79 mmol) was dissolved in dry CH_2Cl_2 (15 mL) and TFA (7.5 mL) at room temperature, and the mixture was stirred at room temperature for 1h. The solvent was then evaporated to dryness under reduced pressure. The residue was redissolved in a $CHCl_3$/MeOH mixture and evaporated to dryness under reduced pressure, and this procedure was repeated two times to remove excess TFA. The resulting slightly yellowish solid and Boc_2O (1.85 g, 8.49 mmol) were dissolved in dry DMF (10 mL). To this was added DIPEA (3.55 mL, 20.4 mmol) at room temperature, and the mixture was allowed to stand at room temperature for 12 h. The solvent was evaporated to dryness under reduced pressure, and the residue was dissolved in a $CHCl_3$/MeOH mixture (ca. 10/1, v/v) and washed with 1N aqueous HCl and brine, dried over $MgSO_4$, filtered, and concentrated under reduced pressure. The residue was purified by recrystallization from $CHCl_3$/MeOH/*n*-hexane (ca. 20/1/50, v/v/v), affording title compound Z-Api(Boc)-(Ac_6c)$_3$-OH as a white solid (4.65 g, 91%). ^{1}H NMR (400 MHz, $(CD_3)_2SO$, 25 ºC): δ 11.65 (s, 1H), 7.82 (s, 1H), 7.45–7.29 (m, 5H), 7.07 (s, 1H), 6.78 (s, 1H), 5.10 (s, 2H), 3.62–3.58 (m, 2H), 3.18 (bs, 2H), 2.07–1.07 + 1.38 (m+s, 43H). ^{13}C NMR (100 MHz, $(CD_3)_2SO$, 25 ºC): δ 175.45, 173.83, 173. 41, 173.15, 155.71, 153.91, 136.89, 128.37, 127.82, 127.09, 78.77, 65.48, 59.28, 58.99, 57.85, 57.85, 57.44, 31.85, 31.49, 31.34, 30.97, 25.26, 25.04, 24.95, 20.97, 20.82. HRMS (ESI-TOF+): *m/z* calcd for ($M+Na^+$), 776.4205; found, 776.4234.

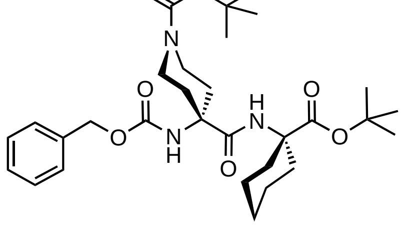

Z-Api(Boc)-Ac$_6$c-O^{t}Bu: To a solution of Z-Api(Boc)-OH (8.55 g, 22.6 mmol) and H-Ac$_6$c-O^{t}Bu (4.50 g, 22.6 mmol), which had been obtained by treatment of Z-Ac$_6$c-O^{t}Bu with 10% Pd-C/H$_2$ in MeOH/CH$_2$Cl$_2$ (2/1, v/v), in dry CH$_2$Cl$_2$ (35 mL) was added DIPEA (4.92 mL, 28.2 mmol) at room temperature. To this was added COMU (9.67 g, 22.6 mmol) at 0 ºC, and the mixture was stirred at 0 ºC for 1 h and further at room temperature for 11 h under N$_2$. The solution was then evaporated to dryness under reduced pressure. The residue was dissolved in EtOAc, and the mixture solution was washed with 1N aqueous HCl, 5% aqueous NaHCO$_3$, and brine, dried over MgSO$_4$, filtered, and concentrated under reduced pressure. The residue was recrystallized from EtOAc/*n*-hexane (ca. 1/5, v/v), affording title compound Z-Api(Boc)-Ac$_6$c-O^{t}Bu as a white solid (10.9 g, 87%). ^{1}H NMR (400 MHz, CDCl$_3$, 25 ºC): δ 7.12 (bs, 1H), 5.11 (s, 2H), 4.89 (s, 1H), 3.77–3.71 (m, 2H), 3.23–3.16 (m, 2H), 2.20–2.13 (m, 2H), 1.97 (s, 2H), 1.94 (s, 2H), 1.74–1.68 (m, 2H), 1.60–1.51 (m, partially overlapping with H$_2$O signal), 1.45 (s, 9H), 1.40 (s, 9H), 1.33–1.20 (m, 4H). ^{13}C NMR (100 MHz, CDCl$_3$, 25 ºC): δ 173.05, 171.53, 155.69, 154.70, 136.01, 128.68, 128.48, 128.24, 80.57, 79.84, 67.16, 58.95, 57.88, 39.43, 32.16, 32.07, 28.47, 27.95, 25.31, 21.48. HRMS (ESI-TOF+): *m/z* calcd for (M+Na$^+$), 582.3150; found, 582.3180.

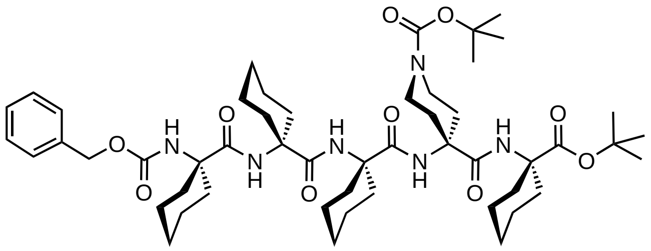

Z-(Ac$_6$c)$_3$-Api(Boc)-Ac$_6$c-O^{t}Bu: To a suspension of Z-(Ac$_6$c)$_3$-OH (3.00 g, 5.69 mmol) and H-Api(Boc)-Ac$_6$c-O^{t}Bu (2.42 g, 5.69 mmol), which had been obtained by treatment of Z-Api(Boc)-Ac$_6$c-O^{t}Bu with 10% Pd-C/H$_2$ in MeOH, in dry CH$_2$Cl$_2$ (11 mL) was added DIPEA (1.09 mL, 6.25 mmol) at room temperature. To this was then added COMU (2.44 g, 5.69 mmol) at 0 ºC, and the mixture was stirred at 0 ºC for 2 h and further at room temperature for 20.5 h under N$_2$. The solution was diluted with EtOAc, and the mixture was washed with 1N aqueous HCl, 5% aqueous NaHCO$_3$, and brine, dried over MgSO$_4$, filtered, and concentrated under reduced pressure. The residue was purified by column chromatography (NH$_2$-SiO$_2$, EtOAc/CH$_2$Cl$_2$ (1/1, v/v)) and further by recrystallization from EtOAc/*n*-hexane (ca. 1/5, v/v), affording title compound Z-(Ac$_6$c)$_3$-Api(Boc)-Ac$_6$c-O^{t}Bu as a white solid (4.23 g, 80%). ^{1}H NMR (400 MHz, CDCl$_3$, 25 ºC): δ 7.40-7.31 (m, 5H), 7.29 (s, 1H), 7.12 (s, 1H), 7.04 (s, 1H), 6.49 (s, 1H), 5.37 (s, 1H), 5.13 (s, 2H), 3.93 (d, J = 12.7 Hz, 2H), 2.86 (t, J = 12.8 Hz, 2H), 2.39 (bs, 2H), 2.07-2.00 (m, 6H), 1.86-1.53 (m, partially overlapping

with H_2O signal), 1.43 (s, 9H), 1.40 (s, 9H), 1.35-1.26 (m, 10H). ^{13}C NMR (100 MHz, $CDCl_3$, 25 ºC): δ 174.71, 173.98, 173.94, 173.91, 172.79, 155.77, 154.83, 136.19, 128.79, 128.52, 127.36, 79.81, 79.26, 67.13, 60.39, 59.76, 59.33, 58.98, 58.79, 39.59, 32.38, 31.52 (bs), 28.56, 27.99, 25.79, 25.39, 25.03, 24.94, 21.74, 21.65, 21.58, 21.41. HRMS (ESI-TOF+): *m/z* calcd for (M+Na^+), 957.5672; found, 957.5746.

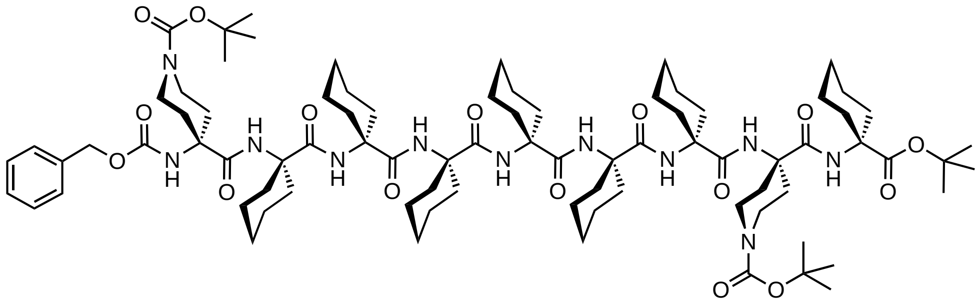

Z-Api(Boc)-(Ac$_6$c)$_6$-Api(Boc)-Ac$_6$c-O^{t}Bu: To a suspension of Z-Api(Boc)-(Ac$_6$c)$_3$-OH (2.30 g, 3.05 mmol) and H-(Ac$_6$c)$_3$-Api(Boc)-Ac$_6$c-O^{t}Bu (2.44 g, 3.05 mmol), which had been obtained by treatment of Z-(Ac$_6$c)$_3$-Api(Boc)-Ac$_6$c-O^{t}Bu with 10% Pd-C/H_2 in CH_2Cl_2/MeOH (1/4, v/v), in dry CH_2Cl_2 (15 mL) was added DIPEA (583 μL, 3.35 mmol) at room temperature. To this was then added COMU (1.30 g, 3.05 mmol) at 0 ºC, and the mixture was stirred at 0 ºC for 2 h and further at room temperature for 2 days under N_2. The solution was diluted with EtOAc, and the mixture was washed with 1N aqueous HCl, 5% aqueous $NaHCO_3$, and brine, dried over $MgSO_4$, filtered, and concentrated under reduced pressure. The residue was purified by column chromatography (SiO_2, EtOAc/CH_2Cl_2 (1/1, v/v)) and further by (NH_2-SiO_2, EtOAc/CH_2Cl_2 (1/1, v/v)), affording title compound Z-Api(Boc)-(Ac$_6$c)$_6$-Api(Boc)-Ac$_6$c-O^{t}Bu as a white solid (3.38 g, 72%). ^1H NMR (400 MHz, $CDCl_3$, 25 ºC): δ 7.42-7.32 (m + s, 5H + 1H), 7.26 (s, overlapping with the residual $CHCl_3$ signal), 7.22 (s, 1H), 7.21 (s, 1H), 7.19 (s, 1H), 7.14 (s, 1H), 7.01 (s, 1H), 6.54 (s, 1H), 5.46 (s, 1H), 5.17 (s, 1H), 3.92 (bs, 2H), 3.89 (bs, 2H), 3.13 (bs, 2H), 2.95 (bs, 2H), 2.05-1.58 (m, partially overlapping with H_2O signal), 1.45 + 1.43 + 1.41 (s + s + s, 9H × 3), 1.32-1.26 (m, 12H). ^{13}C NMR (100 MHz, $CDCl_3$, 25 ºC): δ 176.10, 176.03, 175.94, 175.63, 175.51, 174.38, 174.06, 173.63, 173.39, 156.15, 154.85, 154.57, 136.47, 128.72, 128.32, 127.12, 80.05, 79.99, 79.10, 66.89, 60.46, 59.53, 59.34, 59.32, 59.29, 59.22, 59.02, 58.68, 58.35, 39.60 (bs), 35.40 (bs), 32.65 (bs), 28.55, 28.46, 27.99, 25.73, 25.65, 25.52, 25.29, 25.06, 21.86, 21.68. HRMS (ESI-TOF+): *m/z* calcd for (M+Na^+), 1558.9511; found, 1558.9608.

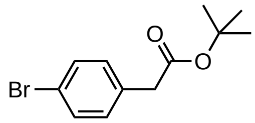

6 (*tert*-Butyl 4-bromophenylacetate): To a solution of 4-bromophenylacetic acid (**5**) (10.0 g, 46.5 mmol) in CH_2Cl_2 containing ca. 8% isobutene (100 mL) was added conc. H_2SO_4 (0.50 mL) at room temperature. The solution was stirred at room temperature for 15 h. The solvent was then evaporated to dryness under reduced pressure, and the residue was dissolved in Et_2O. The mixture was washed with sat. aqueous $NaHCO_3$ and brine, dried over $MgSO_4$, filtered, and concentrated under reduced pressure, affording title compound **6** (8.46 g, 67%) as a slightly yellowish oil, which was used for the next reaction without further purification. ^1H NMR (400 MHz, $CDCl_3$, 25 ºC): δ 7.45-7.42 (m, 2H), 7.15-7.13 (m, 2H), 3.47 (s, 2H), 1.43 (s, 9H). ^{13}C NMR (100 MHz, $CDCl_3$, 25 ºC): δ 170.51, 133.79, 131.67, 131.09, 121.01, 81.26, 42.16, 28.14. HRMS (ESI-TOF+): *m/z* calcd for $(M+Na^+)$, 293.0148; found, 293.0147.

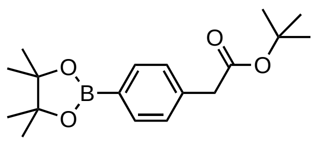

7: Into a two-neck round bottom flask containing *tert*-butyl 4-bromophenylacetate (**6**) (4.00 g, 14.8 mmol), $Pd(dppf)Cl_2 \cdot CH_2Cl_2$ (602 mg, 0.738 mmol), Pin_2B_2 (4.31 g, 17.0 mmol), and KOAc (4.34 g, 44.3 mmol) was added dry 1,4-dioxane (100 mL) under N_2. The reaction mixture was stirred at 75 ºC for 19 h under N_2. After cooling to room temperature, the solvent was evaporated to dryness under reduced pressure. The residue was suspended in *n*-hexane, and the insoluble part was removed by filtration. The filtrate was washed with water and brine, dried over $MgSO_4$, filtered, and concentrated under reduced pressure. Purification by column chromatography (SiO_2, EtOAc/*n*-hexane (1/7, v/v)) afforded title compound **7** (3.39 g, 72%) as a colorless oil. ^1H NMR (400 MHz, $CDCl_3$, 25 ºC): δ 7.77-7.75 (m, 2H), 7.29-7.27 (m, 2H), 3.53 (s, 2H), 1.42 (s, 9H), 1.34 (s, 12H). ^{13}C NMR (100 MHz, $CDCl_3$, 25 ºC): δ 170.74, 138.02, 135.06, 128.68, 83.84, 80.95, 43.09, 28.11, 24.97. HRMS (ESI-TOF+): *m/z* calcd for $(M+Na^+)$, 341.1895; found, 341.1912.

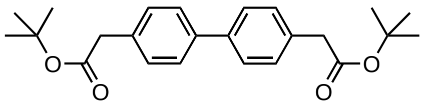

8: Into a two-neck round bottom flask containing **7** (3.00 g, 9.43 mmol), *tert*-butyl 4-bromophenylacetate (**6**) (2.81 g, 10.4 mmol), K_3PO_4 (5.00 g, 23.6 mmol), and $Pd(PPh_3)_4$ (1.09 mg, 0.943 mmol) was added dry 1,4-dioxane (7.5 mL) at room temperature under N_2. The flask was then

subjected to three vacuum/N_2 fill cycles. The reaction mixture was stirred at reflux for 24 h under N_2. After cooling to room temperature, the solution was diluted with EtOAc, and the mixture was washed with water and brine, dried over $MgSO_4$, filtered, and concentrated under reduced pressure. Purification by column chromatography (SiO_2, EtOAc/*n*-hexane (1/9, v/v)) afforded title compound **8** (1.02 g, 30%) as a white solid. ^{1}H NMR (400 MHz, $CDCl_3$, 25 ºC): δ 7.55-7.53 (m, 4H), 7.35-7.33 (m, 4H), 3.57 (s, 4H), 1.46 (s, 18H). ^{13}C NMR (100 MHz, $CDCl_3$, 25 ºC): δ 171.05, 139.57, 133.80, 129.73, 127.22, 81.02, 42.35, 28.18. HRMS (ESI-TOF+): *m/z* calcd for (M+Na^+), 405.2036; found, 405.2049.

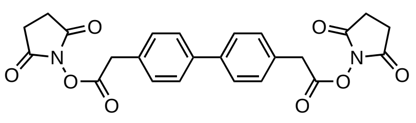

4: To a solution of **8** (1.00 g, 2.61 mmol) in dry CH_2Cl_2 (10 mL) was added TFA (5 mL) at room temperature. The mixture was allowed to stand at room temperature for 3.5 h, and the solvents were evaporated to dryness under reduced pressure. The residue was washed with $CHCl_3$/MeOH/Et_2O (ca. 5/2/20, v/v/v), affording biphenyl-4,4'-diacetic acid (**9**) (548 mg, 78%) as a white solid, which was used for the next reaction without further purification. To a suspension of **9** (538 mg, 1.99 mmol) and *N*-hydroxysuccinimide (504 mg, 4.38 mmol) in dry CH_2Cl_2 (16 mL) was added EDC·HCl (782 mg, 4.08 mmol) at 0 ºC. The reaction mixture was stirred at 0 ºC for 1 h and further at room temperature for 16.5 h under N_2. The solution was then diluted with CH_2Cl_2, and the mixture was washed with 1N aqueous HCl, 5% aqueous $NaHCO_3$, and brine, dried over $MgSO_4$, filtered, and concentrated under reduced pressure. Purification by recrystallization from CH_2Cl_2/*n*-hexane (ca. 1/3, v/v) afforded title compound **4** (886 mg, 96%) as a white solid. ^{1}H NMR (400 MHz, $(CD_3)_2SO$, 25 ºC): δ 7.70-7.67 (m, 4H), 7.46-7.43 (m, 4H), 4.17 (s, 4H), 2.82 (s, 8H). ^{13}C NMR (100 MHz, $(CD_3)_2SO$, 25 ºC): δ 170.16, 167.39, 138.73, 131.66, 129.99, 126.82, 36.25, 25.46. HRMS (ESI-TOF+): *m/z* calcd for (M+Na^+), 487.1112; found, 487.1122.

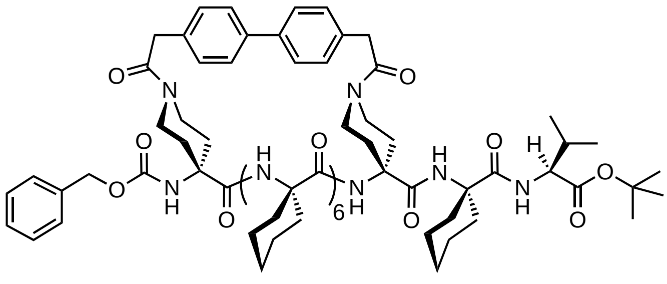

c1-Val-O^{t}Bu: To a solution of Z-Api(HCl)-$(Ac_6c)_6$-Api(HCl)-Ac_6c-OH (1.06 g, 0.783 mmol), which had been obtained by treatment of Z-Api(Boc)-$(Ac_6c)_6$-Api(Boc)-Ac_6c-O^{t}Bu with a mixture of dry

CH_2Cl_2 and 4N HCl in 1,4-dioxane (2/1, v/v), and **4** (400 mg, 0.861 mmol) in dry DMF (260 mL) was added DIPEA (410 μL, 2.35 mmol) at room temperature. The mixture was stirred at room temperature for 24 h under N_2, and the solvents were evaporated to dryness under reduced pressure. The residue was dissolved in a $CHCl_3$/MeOH mixture (ca. 9/1, v/v), and the solution was washed with 1N aqueous HCl, 5% aqueous $NaHCO_3$, and brine, dried over $MgSO_4$, filtered, and concentrated under reduced pressure. The residue was then washed with $CHCl_3$/MeOH/EtOAc (ca. 9/1/50, v/v), affording **c1-OH** (737 mg, 62%) as a white solid, which was used for the next reaction without further purification. To a suspension of **c1-OH** (500 mg, 0.327 mmol) and HCl·H-L-Val-O^{t}Bu (171 mg, 0.818 mmol) in dry CH_2Cl_2 (8 mL) containing DIPEA (205 μL, 1.18 mmol) was added COMU (147 mg, 0.343 mmol) at 0 ºC. The reaction mixture was stirred at 0 ºC for 2.5 h and further at room temperature for 5 h under N_2. The solution was diluted with $CHCl_3$, and the mixture was washed with 1N aqueous HCl, 5% aqueous $NaHCO_3$, and brine, dried over $MgSO_4$, filtered, and concentrated under reduced pressure. The residue was purified by column chromatography (NH_2-SiO_2, CH_2Cl_2/MeOH (95/5, v/v)) and further by size exclusion chromatography (SEC, JAIGEL-2HR ($CHCl_3$/MeOH (9/1, v/v) as eluent), affording title compound **c1-Val-O^{t}Bu** as a white solid (373 mg, 68%). ^{1}H NMR (400 MHz, CD_2Cl_2, 25 ºC): δ 7.57-7.22 (m, 15H), 7.19 (s, 1H), 7.06 + 6.99 (s + s, 1H), 6.92 (s, 1H), 6.51 + 6.50 (s + s, 1H), 6.36 (s, 1H), 6.17 + 6.14 (s + s, 1H), 5.22-5.12 (m, 2H), 4.50-4.47 (m, 1H), 4.40-4.37 (m, 1H), 4.01-3.94 (m, 1H), 3.90-3.66 (m, 4H), 3.61-3.41 (m, 3H), 3.22-3.17 (m, 2H), 2.75-2.72 (m, 1H), 2.60-2.42 (m, 3H), 2.30-0.84 (m, partially overlapping with H_2O signal), 0.80-0.74 (m, 1H), 0.64-0.62 (m, 1H). ^{13}C NMR (100 MHz, $CDCl_3$, 25 ºC): δ 177.22, 176.87, 176.58, 176.43, 175.94, 175.12, 174.80, 174.57, 174.45, 174.34, 174.15, 173.75, 173.47, 172.24, 171.78, 168.91, 168.52, 156.62, 156.49, 139.11, 138.24, 137.06, 137.02, 134.79, 134.23, 128.79, 128.75, 128.28, 128.16, 127.98, 127.46, 127.20, 126.61, 81.60, 80.90, 66.39 (bs), 61.45 (bs), 60.91, 60.29, 59.27, 59.07, 58.59, 58.52, 58.47, 58.41, 58.32, 58.14, 58.09, 57.94, 57.87, 57.76, 57.72, 42.14 (bs), 37.66 (bs), 37.16 (bs), 36.69 (bs), 35.99 (bs), 34.65 (bs), 33.83 (bs), 29.30, 28.17, 28.05, 25.68 (bs), 21.72 (bs), 20.39, 19.99, 19.38, 19.26. HRMS (ESI-TOF+): *m/z* calcd for (M+Na$^+$), 1691.9827; found, 1691.9893.

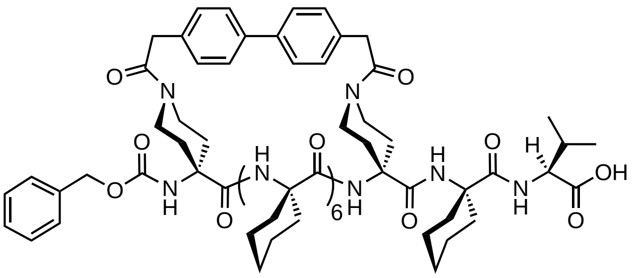

c1-Val-OH: c1-Val-O^{t}Bu (110 mg, 65.9 μmol) was dissolved in CH_2Cl_2 (0.5 mL) and HCO_2H (1.0 mL) at room temperature. The reaction mixture was allowed to stand at room temperature for 80 min, and the solvents were evaporated to dryness under reduced pressure. The residue was reprecipitated from CH_2Cl_2 to Et_2O, affording title compound **c1-Val-OH** (88.6 mg, 83%) as a white solid. ^{1}H NMR (400 MHz, CD_2Cl_2, 25 ºC): δ 10.32 (bs, 1H), 7.8-7.06 (m, 17H), 7.01 + 6.96 (s + s, 1H), 6.76 + 6.74 + 6.72 + 6.66 (s + s + s + s, 2H), 6.53 +6.51 (s, + s, 1H), 6.36 + 6.35 (s +s, 1H), 6.17 + 6.04 (s + s, 1H), 5.18 (q, J = 12.8 Hz, 2H), 4.52-4.46 (m, 2H), 4.17 (t, J = 8.0 Hz, 1H), 3.91-3.74 (m, 5H), 3.62-3.58 (m, 1H), 3.43-3.41 (m, 2H), 3.21-3.14 (m, 1H), 3.10-3.05 (m, 1H), 2.62-2.56 (m, 1H), 2.41-0.90 (m, partially overlapping with H_2O signal), 0.73-0.67 (m, 1H), 0.57-0.54 (m, 1H). ^{13}C NMR (100 MHz, $CDCl_3$, 25 ºC): δ 177.15, 176.61, 176.18, 176.11, 175.62, 175.40, 175.22, 175.11, 174.97, 174.92, 174.77, 174.72, 174.41, 174.37, 174.23, 173.44, 173.37, 172.74, 168.49, 168.47, 167.96, 167.93, 156.63, 138.91, 138.09, 138.07, 137.32, 135.32, 134.72, 134.69, 128.77, 128.39, 128.21, 128.17, 127.39, 127.36, 127.06, 126.99, 66.33, 60.39, 60.21, 59.72, 59.40, 59.16, 59.01, 58.47, 58.41, 58.29, 58.11, 57.92, 57.85, 57.75, 42.15, 41.99, 41.92, 41.75, 37.28, 36.77, 36.49, 36.01, 34.92, 34.77, 34.73, 34.61, 34.44, 34.13, 33.84, 33.73, 30.40, 29.93, 29.89, 29.63, 29.56, 29.38, 28.74, 28.53, 28.48, 28.39, 28.30, 28.02, 27.79, 28.02, 25.91, 25.80, 25.64, 25.43, 22.47, 22.28, 22.14, 22.06, 22.00, 21.92, 21.85, 21.75, 21.70, 21.65, 21.39, 19.61, 19.42, 19.34. HRMS (ESI-TOF+): *m/z* calcd for (M+Na$^+$), 1635.9201; found, 1635.9241.

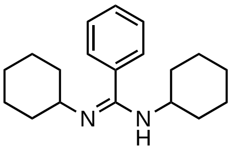

Amidine A3: To a solution of bromobenzene (0.50 mL, 4.78 mmol) in dry THF (7.5 mL) was added dropwise a solution of *n*-butyllithium (1.6 M in *n*-hexane, 3.3 mL, 5.30 mmol) at –78 ºC under N_2. After 1h, to this was added dropwise a solution of *N*,*N*'-dicyclohexylcarbodiimide (1.09 g, 5.26 mmol) in dry THF (5.0 mL) at –78 ºC under N_2. The reaction mixture was gradually warmed to room temperature and stirred at room temperature for 2.5 h under N_2. After cooling to 0 ºC, to this was added water (10 mL), and THF was then removed under reduced pressure. The aqueous solution was

extracted with Et_2O (2 × ca. 35 mL). The combined organic layer was washed with brine, dried over Na_2SO_4, filtered, and concentrated under reduced pressure. The residue was purified by column chromatography (NH_2-SiO_2, *n*-hexane/EtOAc (1/0 to 1/1, v/v)), affording title compound **A3** as a colorless oil (1.13 g, 83%). ^1H NMR (400 MHz, $CDCl_3$, [**A3**]/[AcOH] = 1/1.8, 25 ºC): δ 12.16 (bs, 2H), 7.62-7.53 (m, 3H), 7.27-7.24 (m, partially overlapping with the residual $CHCl_3$ signal), 2.68-2.60 (m, 2H), 2.05 (s, 5H), 1.70-1.64 (m, 8H), 1.53-1.43 (m, 6H), 1.16-1.1.05 (m, 2H), 0.97-0.85 (m, 4H). ^{13}C NMR (100 MHz, $CDCl_3$, [**A3**]/[AcOH] = 1/1.8, 25 ºC): δ 177.57, 164.33, 131.32, 129.64, 127.88, 126.68, 54.57, 33.23, 25.04, 24.58, 23.09. HRMS (ESI-TOF+): *m/z* calcd for ($M+H^+$), 285.2325; found, 285.2339.

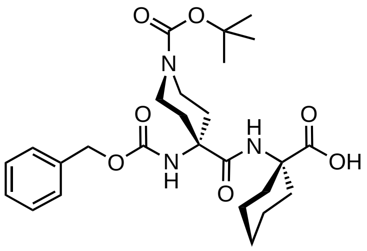

Z-Api(Boc)-Ac$_6$c-OH: To a solution of Z-Api(Boc)-Ac$_6$c-O^{t}Bu (6.00 g, 10.7 mmol) in dry CH_2Cl_2 (20 mL) was added TFA (10 mL) at room temperature. The mixture was allowed to stand at room temperature for 1.75 h, and the solvents were evaporated to dryness under reduced pressure. The residue was then suspended in dry DMF (25 mL), and to this was added Boc_2O (2.92 g, 13.4 mmol) and DIPEA (5.60 mL, 32.2 mmol) at room temperature. The reaction mixture was stirred at room temperature for 18 h, and the solvent was evaporated to dryness under reduced pressure. The residue was dissolved in a $CHCl_3$/MeOH (ca. 9/1, v/v) mixture, and the solution was washed with 1N aqueous HCl and brine, dried over $MgSO_4$, filtered, and concentrated under reduced pressure. Purification by recrystallization from $CHCl_3$/Et_2O/*n*-hexane (ca. 1/3/10, v/v/v) afforded title compound Z-Api(Boc)-Ac$_6$c-OH as a white solid (5.43 g, 94%). ^1H NMR (400 MHz, $(CD_3)_2SO$, 25 ºC): δ 7.37-7.31 (s + m, 6H), 7.18 (s, 1H), 5.04 (s, 2H), 3.65 (bs, 2H), 3.62 (bs, 2H), 3.05 (bs, 2H), 1.98-1.94 (m, 4H), 1.85-1.78 (m, 2H), 1.60-1.54 (m, 2H), 1.44-1.23 (m + s, 4H + 9H), 1.17-1.13 (m, 1H). ^{13}C NMR (100 MHz, $(CD_3)_2SO$, 25 ºC): δ 175.53, 172.84, 155.21, 153.90, 136.87, 128.36, 127.89, 127.74, 78.67, 65.38, 57.72, 57.21, 31.52, 31.11 (bs), 28.09, 24.99, 20.92, . HRMS (ESI-TOF+): *m/z* calcd for ($M+Na^+$), 526.2524; found, 526.2557.

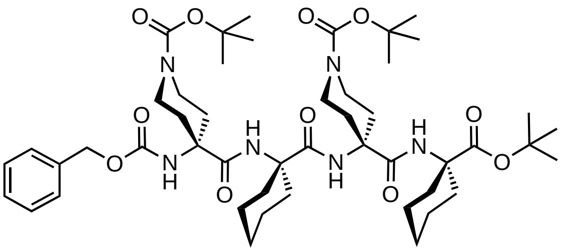

Z-[Api(Boc)-Ac$_6$c]$_2$-O^{t}Bu: To a solution of Z-Api(Boc)-Ac$_6$c-OH (2.16 g, 4.29 mmol) and H-Api(Boc)-Ac$_6$c-O^{t}Bu (1.83 g, 4.29 mmol), which had been obtained by treatment of Z-Api(Boc)-Ac$_6$c-O^{t}Bu with 10% Pd-C/H_2 in MeOH, in dry CH_2Cl_2 (8 mL) was added DIPEA (934 μL, 5.36 mmol) at room temperature. To this was added COMU (1.84 g, 4.29 mmol) at 0 ºC, and the mixture was stirred at 0 ºC for 1 h and further at room temperature for 15 h under N_2. The solution was then diluted with EtOAc, and the mixture solution was washed with 1N aqueous HCl, 5% aqueous $NaHCO_3$, and brine, dried over $MgSO_4$, filtered, and concentrated under reduced pressure. Purification by column chromatography (SiO_2, CH_2Cl_2/EtOAc, 3/2, v/v) afforded title compound Z-[Api(Boc)-Ac$_6$c]$_2$-O^{t}Bu as a white solid (2.20 g, 56%). ^{1}H NMR (400 MHz, $CDCl_3$, 25 ºC): δ 7.35-7.29 (m, 5H), 7.22 (s, 1H), 6.84 (s, 1H), 6.39 (s, 1H), 5.35 (s, 1H), 5.12 (s, 2H), 3.90 (t, J = 14 Hz), 3.14-3.08 (m, 2H), 2.81-2.74 (m, 2H), 2.19-2.02 (m, 8H), 1.89-1.75 (m, 8H), 1.96-1.51 (m, partially overlapping with H_2O signal), 1.453 (s, 9H), 1.451 (s, 9H), 1.41 (s, 9H), 1.28-1.13 (m, 6H). ^{13}C NMR (100 MHz, $CDCl_3$, 25 ºC): δ 173.74, 173.05, 172.49, 155.77, 154.73, 154.59, 136.15, 128.79, 128.50, 127.23, 80.14, 79.94, 79.52, 67.32, 59.31, 58.35, 39.72 (br), 38.81 (br), 32.19, 31.65 (br), 31.04 (br), 28.53, 28.44, 27.98, 25.68, 24.98, 21.59. HRMS (ESI-TOF+): m/z calcd for (M+Na$^+$), 933.5308; found, 933.5344.

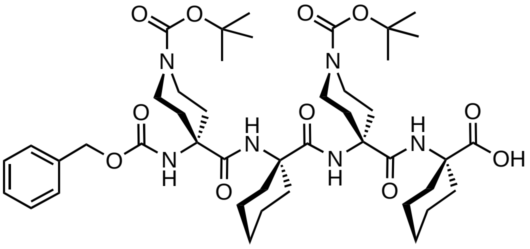

Z-[Api(Boc)-Ac$_6$c]$_2$-OH: To a solution of Z-[Api(Boc)-Ac$_6$c]$_2$-O^{t}Bu (2.00 g, 2.19 mmol) in dry CH_2Cl_2 (15 mL) was added TFA (7.5 mL) at room temperature. The reaction mixture was stirred at room temperature for 2.5 h, and the solvents were removed to dryness under reduced pressure. The residue was dissolved in dry DMF (7.0 mL). To this were added Boc_2O (1.20 g, 5.49 mmol) and DIPEA (1.91 mL, 11.0 mmol) at room temperature, and the mixture was stirred at room temperature for 11 h. The solvent was then removed to dryness under reduced pressure. The residue was dissolved in a $CHCl_3$/MeOH (9/1, v/v) mixture, the mixture solution was washed with 1N aqueous HCl and

brine, dried over $MgSO_4$, filtered, and concentrated under reduced pressure. Purification by recrystallization from $CHCl_3$/MeOH/*n*-hexane (ca. 9/1/50, v/v/v) afforded title compound Z-[Api(Boc)-Ac$_6$c]$_2$-OH as a white solid (1.69 g, 90%). ^{1}H NMR (400 MHz, $(CD_3)_2SO$, 25 ºC): δ 11.83 (s, 1H), 7.91 (s, 1H), 7.38-7.30 (m, 5H), 7.14 (s, 1H), 7.00 (s, 1H), 5.09 (s, 2H), 3.70-3.58 (m, 4H), 3.19 (bs, 2H), 2.72 (bs, 2H), 2.02-1.86 (m, 10H), 1.72-1.52 (m, 11H), 1.43-1.33 (m + s + s, 4H + 9H + 9H), 1.22-1.20 (m, 3H). ^{13}C NMR (100 MHz, $(CD_3)_2SO$, 25 ºC): δ 175.35, 173.84, 173.41, 172.38, 155.91, 153.89, 153.85, 136.78, 128.39, 127.85, 127.04, 78.78, 78.57, 65.67, 59.31, 57.99, 57.43, 57.21, 31.82, 31.53, 31.17, 30.46 (bs), 28.03, 28.01, 25.19, 24.90, 20.90. HRMS (ESI-TOF+): *m/z* calcd for (M+Na$^+$), 877.4682; found, 877.4722.

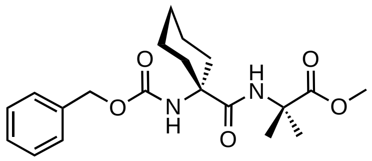

Z-Ac$_6$c-Aib-OMe: To a suspension of Z-Ac$_6$c-OH (10.83 g, 0.0372 mol) and HCl·H-Aib-OMe (6.00 g, 0.0391 mol) in dry CH_2Cl_2 (50 mL) was added DIPEA (19.8 mL, 0.113 mol) at room temperature. To this was added COMU (15.93 g, 0.0372 mol) at 0 ºC, and the mixture was stirred at 0 ºC for 1 h and further at room temperature for 14.5 h under N_2. The solvent was then evaporated to dryness under reduced pressure. The residue was dissolved in EtOAc, and the solution was washed with 1N aqueous HCl, 5% aqueous $NaHCO_3$, and brine, dried over $MgSO_4$, filtered, and concentrated under reduced pressure. The residue was purified by washing with Et_2O and further by column chromatography (SiO_2, EtOAc/CH_2Cl_2 (3/7, v/v)), affording title compound Z-Ac$_6$c-Aib-OMe as a white solid (8.96 g, 64%). ^{1}H NMR (400 MHz, $CDCl_3$, 25 ºC): δ 7.34–7.28 (m, 5H), 7.16 (s, 1H), 5.09 (s, 2H), 5.00 (s, 1H), 3.67 (s, 3H), 2.01–1.97 (m, 2H), 1.89–1.82 (m, 2H), 1.63–1.56 (m, 3H), 1.45 + 1.42–1.25 (s + m, 9H). ^{13}C NMR (100 MHz, $CDCl_3$, 25 ºC): δ 175.10, 173.55, 155.33, 136.3927, 128.60, 128.26, 128.12, 66.85, 59.58, 56.19, 52.48, 32.12, 25.20, 24.65, 21.40. HRMS (ESI-TOF+): *m/z* calcd for (M+Na$^+$), 399.1890; found, 399.1902.

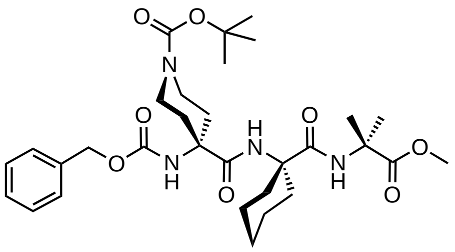

Z-Api(Boc)-Ac$_6$c-Aib-OMe: To a suspension of Z-Api(Boc)-OH (4.07 g, 10.8 mmol) and H-Ac$_6$c-Aib-OMe (2.87 g, 11.8 mmol), which had been obtained by treatment of Z-Ac$_6$c-Aib-OMe with 10% Pd-C/H_2 in MeOH, in dry CH_2Cl_2 (15 mL) was added DIPEA (3.75 mL, 21.5 mmol) at room temperature. To this was then added COMU (4.61 g, 10.8 mmol) at 0 ºC, and the mixture was stirred at 0 ºC for 1 h and further at room temperature for 20 h under N_2. The solution was diluted with EtOAc, and the mixture was washed with 1N aqueous HCl, 5% aqueous $NaHCO_3$, and brine, dried over $MgSO_4$, filtered, and concentrated under reduced pressure. The residue was purified by column chromatography (SiO_2, EtOAc/CH_2Cl_2 (3/7 to 5/5, v/v)), affording title compound Z-Api(Boc)-Ac$_6$c-Aib-OMe as a white solid (5.01 g, 77%). ^{1}H NMR (400 MHz, $CDCl_3$, 25 ºC): δ 7.40–7.32 (m, 5H), 7.24 (s, 1H), 6.33 (s, 1H), 5.18 (s, 1H), 5.12 (s, 2H), 3.89 (bd, J = 12.2 Hz, 2H), 3.69 (s, 3H), 3.10–3.04 (m, 2H), 2.17–2.10 (m, 2H), 1.98–1.80 (m, 6H), 1.61–1.55 (m, partially overlapping with H_2O signal), 1.48 (s, 6H), 1.45 (s, 9H), 1.29–1.17 (m, 3H). ^{13}C NMR (100 MHz, $CDCl_3$, 25 ºC): δ 175.21, 173.46, 172.08, 155.67, 154.59, 135.92, 128.77, 128.61, 128.19, 80.00, 67.34, 59.66, 58.38, 55.94, 52.25, 39.31, 31.94, 31.64, 28.44, 25.14, 24.80, 21.42. HRMS (ESI-TOF+): *m/z* calcd for (M+Na$^+$), 625.3208; found, 625.3245.

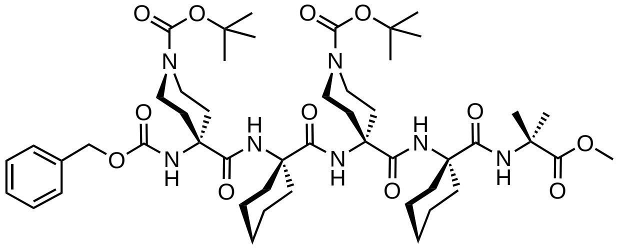

Z-[Api(Boc)-Ac$_6$c]$_2$-Aib-OMe: To a solution of Z-Api(Boc)-Ac$_6$c-OH (1.18 g, 2.35 mmol) and H-Api(Boc)-Ac$_6$c-Aib-OMe (1.10 g, 2.35 mmol), which had been obtained by treatment of Z-Api(Boc)-Ac$_6$c-Aib-OMe with 10% Pd-C/H_2 in MeOH, in dry CH_2Cl_2 (8 mL) was added DIPEA (511 μL, 2.93 mmol) at room temperature. To this was added COMU (1.01 g, 2.35 mmol) at 0 ºC, and the mixture was stirred at 0 ºC for 1 h and further at room temperature for 27 h under N_2. The solution was then diluted with EtOAc, and the mixture solution was washed with 1N aqueous HCl, 5% aqueous $NaHCO_3$, and brine, dried over $MgSO_4$, filtered, and concentrated under reduced pressure. The residue was washed with a EtOAc/*n*-hexane (ca. 1/2, v/v) mixture, affording title compound Z-

[Api(Boc)-Ac_6c]$_2$-Aib-OMe as a white solid (1.91 g, 85%). ^{1}H NMR (400 MHz, $CDCl_3$, 25 ºC): δ 7.35-7.29 (m, 5H), 7.25 (s, 1H), 7.13 (s, 1H), 6.84 (s, 1H), 6.42 (s, 1H), 5.54 (s, 1H), 5.15 (s, 2H), 3.94-3.83 (m, 4H), 3.67 (s, 3H), 3.15 (m, 2H), 2.79 (m, 2H), 2.31 (bs, 2H), 2.16-2.04 (m, 4H), 1.93-1.56 (m, partially overlapping with H_2O signal), 1.48 (s, 6H), 1.46 + 1.45 (s + s, 18H), 1.32-1.15 (m, 7H). ^{13}C NMR (100 MHz, $(CD_3)_2SO$, 25 ºC): δ 175.13, 174.34, 174.18, 173.66, 172.38, 156.01, 153.75, 153.68, 136.76, 128.31, 127.70, 126.65, 78.68, 78.56, 65.60, 58.96, 58.69, 57.31, 57.17, 54.73, 51.44, 31.72 (br), 27.98, 25.08, 24.81, 24.61, 21.08, 20.95. HRMS (ESI-TOF+): *m/z* calcd for ($M+Na^+$), 976.5366; found, 976.5426.

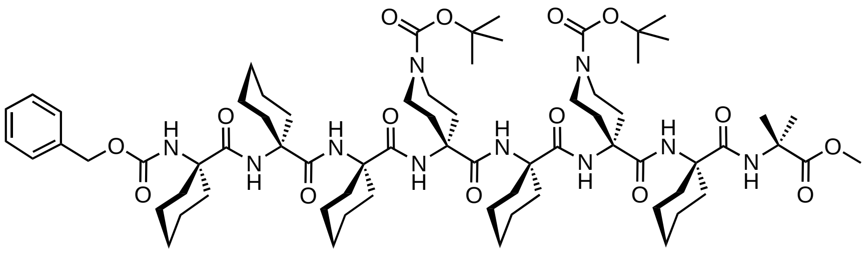

Z-(Ac_6c)$_3$-[Api(Boc)-Ac_6c]$_2$-Aib-OMe: To a suspension of Z-(Ac_6c)$_3$-OH (940 mg, 1.78 mmol), which had been obtained by treatment of Z-(Ac_6c)$_3$-O^{t}Bu with TFA/CH_2Cl_2 (1/2, v/v), and H-[Api(Boc)-Ac_6c]$_2$-Aib-OMe (1.46 g, 1.78 mmol), which had been obtained by treatment of Z-[Api(Boc)-Ac_6c]$_2$-Aib-OMe with 10% Pd-C/H_2 in MeOH/CH_2Cl_2 (3/1, v/v), in dry CH_2Cl_2 (8 mL) and DIPEA (388 μL, 2.23 mmol) was added COMU (763 mg, 1.78 mmol) at 0 ºC. The mixture was stirred at 0 ºC for 1 h and further at room temperature for 18 h under N_2. The solution was then diluted with EtOAc, and the mixture solution was washed with 1N aqueous HCl, 5% aqueous $NaHCO_3$, and brine, dried over $MgSO_4$, filtered, and concentrated under reduced pressure. Purification by recrystallization from CH_2Cl_2/EtOAc/*n*-hexane (ca. 1/10/30, v/v/v) afforded title compound Z-(Ac_6c)$_3$-[Api(Boc)-Ac_6c]$_2$-Aib-OMe as a white solid (2.18 g, 92%). ^{1}H NMR (400 MHz, $CDCl_3$, 25 ºC): δ 7.47 (s, 1H), 7.41-7.32 (m, 7H), 7.23 (s, 1H), 7.13 (s, 1H), 6.96 (s, 1H), 6.56 (s, 1H), 5.47 (s, 1H), 5.16 (s, 2H), 3.90 (s, 4H), 3.67 (s, 3H), 3.18-1.55 (m, partially overlapping with H_2O signal), 1.51 (s, 6H), 1.44 (s, 9H), 1.43 (s, 9H). ^{13}C NMR (100 MHz, $CDCl_3$, 25 ºC): δ 176.45, 176.34, 175.79, 174.82, 174.79, 174.71, 174.65, 173.43, 156.00, 154.78, 136.32, 128.76, 128.44, 127.12, 79.62, 79.29, 66.99, 60.16, 59.85, 59.77, 59.36, 58.83, 58.33, 57.70, 55.77, 51.97, 40.34, 39.29, 35.07, 34.31, 28.13 (br), 25.66, 25.49, 25.20, 24.97, 21.77 (br), 21.54 (br), 21.38. HRMS (ESI-TOF+): *m/z* calcd for ($M+Na^+$), 1351.7888; found, 1351.7953.

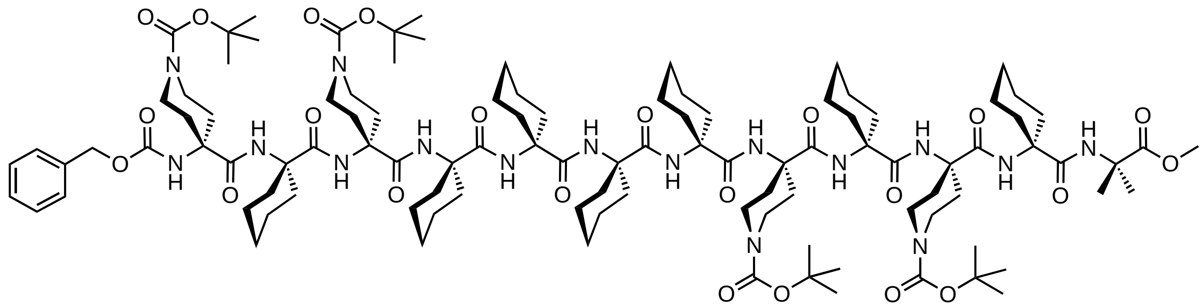

Z-[Api(Boc)-Ac$_6$c]$_2$-(Ac$_6$c)$_3$-[Api(Boc)-Ac$_6$c]$_2$-Aib-OMe: To a solution of Z-[Api(Boc)-Ac$_6$c]$_2$-OH (1.29 g, 1.50 mmol) and H-(Ac$_6$c)$_3$-[Api(Boc)-Ac$_6$c]$_2$-Aib-OMe (1.80 g, 1.50 mmol), which had been obtained by treatment of Z-(Ac$_6$c)$_3$-[Api(Boc)-Ac$_6$c]$_2$-Aib-OMe with 10% Pd-C/H_2 in MeOH/CH_2Cl_2 (3/1, v/v), in dry CH_2Cl_2 (8 mL) and DIPEA (327 μL, 1.88 mmol) was added COMU (644 mg, 1.50 mmol) at 0 ºC. The mixture was stirred at 0 ºC for 2 h and further at room temperature for 2 days under N_2. The solution was then diluted with CH_2Cl_2/MeOH (ca. 10/1, v/v), and the mixture solution was washed with 1N aqueous HCl, 5% aqueous $NaHCO_3$, and brine, dried over $MgSO_4$, filtered, and concentrated under reduced pressure. Purification by recrystallization from CH_2Cl_2/MeOH/EtOAc/*n*-hexane (ca. 1//0.1/5/10, v/v/v/v) afforded title compound Z-[Api(Boc)-Ac$_6$c]$_2$-(Ac$_6$c)$_3$-[Api(Boc)-Ac$_6$c]$_2$-Aib-OMe as a white solid (2.48 g, 81%). ^{1}H NMR (400 MHz, $CDCl_3$, 25 ºC): δ 7.49 (s, 1H), 7.35 (s, 1H), 7.39-7.30 (m, 7H), 7.19 (s, 1H), 7.14 (s + s + s, 3H), 7.03 (s, 1H), 7.02 (s, 1H), 6.54 (s, 1H), 5.85 (bs, 1H), 5.18 (bd, J = 45 Hz, 2H), 3.88 (bs, 8H), 3.67 (s, 3H), 3.37-1.57 (m, partially overlapping with H_2O signal), 1.52 (s, 6H), 1.48 + 1.46 + 1.430 + 1.427 (s + s + s + s, 9H × 4), 1.2 (bs, 15H). ^{13}C NMR (100 MHz, $CDCl_3$, 25 ºC): δ 176.77, 176.72, 176.10, 176.05, 175.97, 175.62, 175.40, 175.01, 174.91, 174.30, 174.24 174.01, 156.54, 154.83, 154.68, 154.59, 136.87, 128.80, 128.13, 126.53, 79.94, 79.86, 79.43, 79.29, 66.74, 60.06, 59.62, 59.10, 59.05, 58.91, 58.86, 58.38, 58.24, 57.52, 57.28, 55.94, 52.06, 39.72 (br), 39.27 (br), 35.29 (br), 34.80 (br), 34.07 (br), 29.72 (br), 29.29 (br), 28.71 (br), 28.53, 28.47, 25.50 (br), 25.08 (br), 24.45 (br), 21.65 (br). HRMS (ESI-TOF+): *m/z* calcd for (M+Na$^+$), 2054.2204; found, 2054.2277.

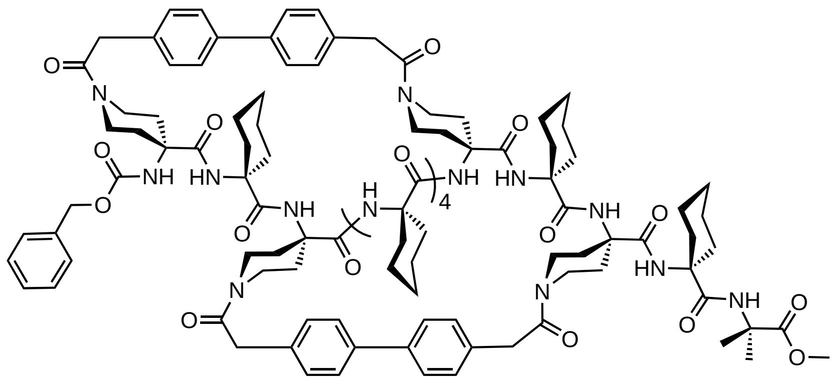

dc2-Aib-OMe: To a solution of Z-[Api(HCl)-Ac$_6$c]$_2$-(Ac$_6$c)$_3$-[Api(HCl)-Ac$_6$c]$_2$-Aib-OMe (100 mg, 56.2 μmol), which had been obtained by treatment of Z-[Api(Boc)-Ac$_6$c]$_2$-(Ac$_6$c)$_3$-[Api(Boc)-Ac$_6$c]$_2$-Aib-OMe with a mixture of CH_2Cl_2, MeOH, and 4N HCl in 1,4-dioxane (2/1/3, v/v/v), and **4** (55.0 mg, 118 μmol) in dry DMF (19 mL) was added DIPEA (59 μL, 338 μmol) at room temperature. The mixture was stirred at room temperature for 2 days under N_2, and the solvent was evaporated to dryness under reduced pressure. The residue was dissolved in a $CHCl_3$/MeOH mixture (ca. 9/1, v/v), and the solution was washed with 1N aqueous HCl, 5% aqueous $NaHCO_3$, and brine, dried over $MgSO_4$, filtered, and concentrated under reduced pressure. Purification by size exclusion chromatography (SEC, JAIGEL-2HR ($CHCl_3$/MeOH (9/1, v/v) as eluent) and further by reprecipitation from CH_2Cl_2 to EtOAc/*n*-hexane (1/1, v/v) afforded title compound **dc2-Aib-OMe** (68 mg, 58%) as a white solid. ^{1}H NMR (400 MHz, CD_2Cl_2/$(CD_3)_2SO$ = 475/25 (v/v), 25 ºC): δ 7.82 (s, 1H), 7.56-7.45 (m, 8H), 7.40 (s, 1H), 7.38-7.33 (m, 7H), 7.26-7.20 (m, 9H), 7.18 (s, 1H), 7.11 (s, 1H), 6.87 (s, 1H), 6.73 (s, 1H), 6.69 (s, 1H), 6.40 (s, 1H), 6.39 (s, 1H), 5.18 (q, *J* = 13.3 Hz, 2H), 4.48-4.32 (m, 4H), 3.94-3.68 (m, 8H), 3.63 (s, 3H), 3.58-3.54 (m, 1H), 3.42-3.19 (m, 8H), 2.63-0.73 (m, partially overlapping with H_2O signal), 0.58-0.52 (m, 1H), 0.27 (bs, 1H). ^{13}C NMR (100 MHz, CD_2Cl_2/$(CD_3)_2SO$ = 475/50 (v/v), 25 ºC): δ 175.94, 175.09, 174.87, 174.63, 174.60, 174.31, 174.24, 174.09, 174.03, 173.84, 173.46, 172.55, 167.85, 167.27, 167.17, 167.03, 156.15, 138.48, 137.93, 137.45, 137.25, 136.11, 134.52, 134.34, 133.78, 133.65, 127.97, 127.62, 127.57, 127.46, 127.37, 127.11, 127.11, 126.83, 126.42, 126.42, 126.27, 126.16, 125.93, 65.74, 59.02, 58.41, 58.10, 57.39, 57.28, 57.21, 57.13, 56.98, 56.38, 56.19, 54.77, 50.97, 41.78, 41.63, 41.52, 41.43, 40.96, 40.91, 40.55, 36.62, 36.40, 36.10, 35.17, 34.22, 34.01, 33.89, 33.74, 33.24, 33.13, 29.79, 28.42, 27.88, 27.67, 27.51, 27.05, 26.96, 26.89, 26.60, 25.11, 25.06, 24.91, 24.82, 24.72, 24.57, 24.34, 23.33, 21.73, 21.60, 21.53, 21.27, 21.09, 21.04, 21.00, 20.92, 20.85, 20.79, 20.67, 20.50. HRMS (ESI-TOF+): *m/z* calcd for (M+Na$^+$), 2122.1468; found, 2122.1426.

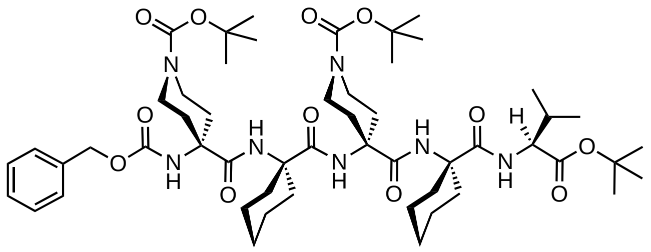

Z-[Api(Boc)-Ac$_6$c]$_2$-L-Val-O^{t}Bu: To a solution of Z-[Api(Boc)-Ac$_6$c]$_2$-OH (1.00 g, 1.17 mmol) and HCl·H-L-Val-O^{t}Bu (490 mg, 2.34 mmol) in dry CH_2Cl_2 (7 mL) and DIPEA (713 μL, 4.09 mmol) was added COMU (506 mg, 1.18 mmol) at 0 ºC. The mixture was stirred at 0 ºC for 1 h and further at room temperature for 12 h under N_2. The solvent was evaporated to dryness under reduced pressure. The residue was dissolved in EtOAc, and the solution was washed with 1N aqueous HCl, 5% aqueous $NaHCO_3$, and brine, dried over $MgSO_4$, filtered, and concentrated under reduced pressure. Purification by column chromatography (NH_2-SiO_2, CH_2Cl_2/EtOAc/MeOH, 1/1/0.1, v/v/v) and further by recrystallization from EtOAc/*n*-hexane (ca. 1/3, v/v) afforded title compound Z-[Api(Boc)-Ac$_6$c]$_2$-L-Val-O^{t}Bu as a white solid (969 mg, 82%). ^{1}H NMR (400 MHz, $CDCl_3$, 25 ºC): δ 7.37-7.28 (m, 5H), 7.21 (d, J = 8.1 Hz, 1H), 7.01 (s, 1H), 6.95 (s, 1H), 6.52 (s, 1H), 5.63 (s, 1H), 5.14 (q, J = 12.7 Hz, 2H), 4.18 (q, J = 7.2 Hz, 1H), 4.00 (bs, 1H), 3.84 (bs, 3H), 3.18-3.09 (m, 2H), 2.83 (bs, 1H), 2.67 (t, J = 12.3 Hz, 1H), 2.43 (bs, 1H), 2.28 (bs, 1H), 2.20-1.57 (m, partially overlapping with H_2O signal), 1.45 (s, 18H), 1.42 (s, 9H), 1.29-1.19 (m, 6H), 0.97-0.94 (m, 6H). ^{13}C NMR (100 MHz, $CDCl_3$, 25 ºC): δ 175.52, 174.38, 173.26, 172.85, 171.25, 156.19, 154.67, 154.51, 136.56, 128.84, 128.29, 126.72, 80.74, 80.07, 66.97, 60.55, 60.31, 59.19, 58.33, 39.83 (bs), 39.12 (bs), 32.55 (bs), 31.47 (bs), 30.50 (bs), 29.93, 28.58, 28.51, 28.08, 25.64, 25.05, 21.81, 21.63, 21.49, 19.33, 19.28. HRMS (ESI-TOF+): *m/z* calcd for (M+Na^+), 1032.5992; found, 1032.6036.

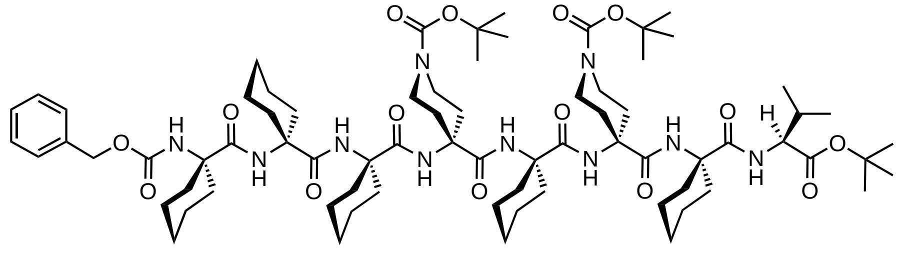

Z-(Ac$_6$c)$_3$-[Api(Boc)-Ac$_6$c]$_2$-L-Val-O^{t}Bu: To a suspension of H-[Api(Boc)-Ac$_6$c]$_2$-L-Val-O^{t}Bu (798 mg, 0.911 mmol), which had been obtained by treatment of Z-[Api(Boc)-Ac$_6$c]$_2$-L-Val-O^{t}Bu with 10% Pd-C/H_2 in CH_2Cl_2/MeOH (1/3, v/v), and Z-(Ac$_6$c)$_3$-OH (480 mg, 0.911 mmol) in dry CH_2Cl_2 (5 mL) and DIPEA (198 μL, 1.14 mmol) was added COMU (390 mg, 0.911 mmol) at 0 ºC. The mixture was stirred at 0 ºC for 1 h and further at room temperature for 11.5 h under N_2. The solution

was diluted with EtOAc, and the mixture was washed with 1N aqueous HCl, 5% aqueous $NaHCO_3$, and brine, dried over $MgSO_4$, filtered, and concentrated under reduced pressure. Purification by column chromatography (SiO_2, EtOAc/*n*-hexane (1/1, v/v) afforded title compound Z-(Ac_6c)$_3$-[Api(Boc)-Ac_6c]$_2$-L-Val-O^{t}Bu as a white solid (969 mg, 82%). ^{1}H NMR (400 MHz, $CDCl_3$, 25 °C): δ 7.46 (s, 1H), 7.41-7.31 (m, 7H), 7.19 (s, 1H), 7.12 (s, 1H), 7.07 (bs, 1H), 6.57 (s, 1H), 5.49 (s, 1H), 5.16 (bs, 2H), 4.18 (t, J = 7.3 Hz, 1H), 3.89 (bs, 4H), 3.12-1.57 (m, partially overlapping with H_2O signal), 1.44 (s, 9H), 1.43 (s, 18H), 1.34-1.24 (m, 11H), 0.98-0.96 (m, 6H).. ^{13}C NMR (100 MHz, $CDCl_3$, 25 °C): δ 176.37, 175.89, 175.47, 174.82, 174.68, 174.47, 173.34, 171.13, 156.01, 154.86, 154.76, 136.42, 128.79, 128.43, 127.09, 80.51 (bs), 79.58, 79.24, 66.96, 60.64, 59.96, 59.87, 59.40, 58.89, 58.64, 57.73, 40.17 (bs), 39.21 (bs), 34.94 (bs), 30.00, 28.56, 28.53, 28.09, 25.73, 25.52, 25.25, 25.02, 21.63, 21.42, 19.34, 19.24. HRMS (ESI-TOF+): *m/z* calcd for (M+Na$^+$), 1407.8514; found, 1407.8571.

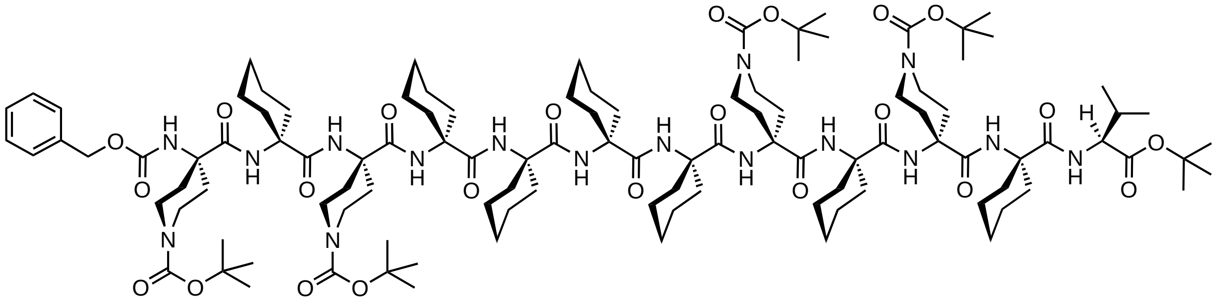

Z-[Api(Boc)-Ac_6c]$_2$-(Ac_6c)$_3$-[Api(Boc)-Ac_6c]$_2$-L-Val-O^{t}Bu: To a solution of H-(Ac_6c)$_3$-[Api(Boc)-Ac_6c]$_2$-L-Val-O^{t}Bu (880 mg, 0.703 mmol), which had been obtained by treatment of Z-(Ac_6c)$_3$-[Api(Boc)-Ac_6c]$_2$-L-Val-O^{t}Bu with 10% Pd-C/H_2 in CH_2Cl_2/MeOH (1/3, v/v), and Z-[Api(Boc)-Ac_6c]$_2$-OH (601 mg, 0.703 mmol) in dry CH_2Cl_2 (6 mL) and DIPEA (153 μL, 0.879 mmol) was added COMU (304 mg, 0.710 mmol) at 0 °C. The mixture was stirred at 0 °C for 1 h and further at room temperature for 17 h under N_2. The solution was diluted with EtOAc, and the solution was washed with 1N aqueous HCl, 5% aqueous $NaHCO_3$, and brine, dried over $MgSO_4$, filtered, and concentrated under reduced pressure. Purification by column chromatography (SiO_2, CH_2Cl_2/EtOAc (1/3, v/v)) afforded title compound Z-[Api(Boc)-Ac_6c]$_2$-(Ac_6c)$_3$-[Api(Boc)-Ac_6c]$_2$-L-Val-O^{t}Bu as a white solid (992 mg, 82%). ^{1}H NMR (400 MHz, $CDCl_3$, 25 °C): δ 7.51 (s, 1H), 7.38-7.30 (m, 8H), 7.21-7.04 (m, 6H), 6.72 (s, 1H), 6.27 (bs, 1H), 5.22-5.13 (m, 2H), 4.13 (bs, 1H), 3.87 (bs, 8H), 3.39-1.57 (m, partially overlapping with H_2O signal), 1.48 + 1.46 + 1.43 (s + s + s, 45H), 1.24 (bs, 13H), 1.02 (bs, 1H), 0.97 (d, J = 6.5 Hz, 6H). ^{13}C NMR (100 MHz, $CDCl_3$, 25 °C): δ 176.88, 176.35, 176.11, 175.70, 174.96 (bs), 174.47 (bs), 174.31, 173.66 (bs), 171.53, 156.59, 154.94, 154.73, 154.52, 136.98, 128.83,

128.07, 126.31, 81.20 (bs), 80.68, 79.91, 79.81, 79.38, 79.22, 66.61, 61.37 (bs), 60.67 (bs), 60.35 (bs), 59.81, 59.13, 58.99, 58.48 (bs), 58.32, 57.63, 57.32, 40.26 (bs), 39.31 (bs), 35.22 (bs), 34.06 (bs), 29.50, 28.57, 28.52, 28.07, 25.82 (bs), 25.56 (bs), 25.14 (bs), 22.03 (bs), 21.69 (bs), 19.88, 19.36. HRMS (ESI-TOF+): *m/z* calcd for (M+Na^+), 2110.2830; found, 2110.2894.

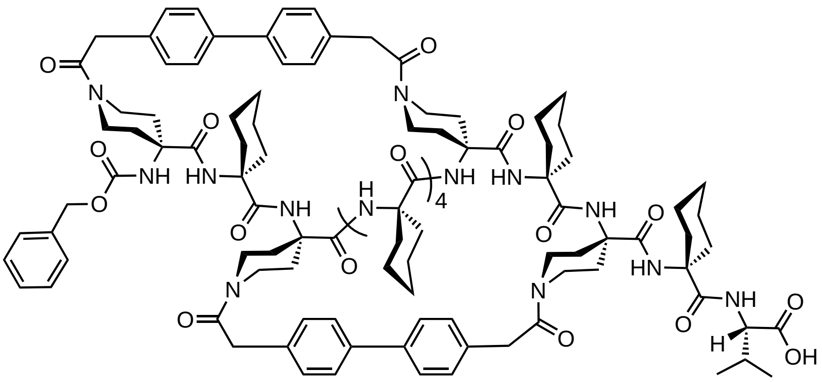

dc2-Val-OH: To a suspension of Z-[Api(HCl)-$Ac_6c]_2$-$(Ac_6c)_3$-[Api(HCl)-$Ac_6c]_2$-L-Val-OH (397 mg, 0.223 mmol), which had been obtained by treatment of Z-[Api(Boc)-$Ac_6c]_2$-$(Ac_6c)_3$-[Api(Boc)-$Ac_6c]_2$-L-Val-O^{t}Bu with dry CH_2Cl_2/HCO_2H (1/2, v/v) and then treatment with a mixture of CH_2Cl_2, MeOH, and 4N HCl in 1,4-dioxane (3/1/1, v/v/v), in dry DMF (50 mL), dry CH_2Cl_2 (14 mL), and DIPEA (195 μL, 1.16 mmol) was added a solution of **4** (218 mg, 0.469 mmol) in dry DMF (10 mL) at room temperature. The mixture was stirred at room temperature for 2 days under N_2, and the solvent was evaporated to dryness under reduced pressure. The residue was dissolved in a $CHCl_3$/MeOH mixture (ca. 9/1, v/v), and the solution was washed with 1N aqueous HCl, 5% aqueous $NaHCO_3$, and brine, dried over $MgSO_4$, filtered, and concentrated under reduced pressure (<50 ºC). Purification by column chromatography (NH_2-SiO_2, $CHCl_3$/MeOH (8/2, v/v) to $CHCl_3$/MeOH/AcOH (7/2/1, v/v/v)) and further by size exclusion chromatography (SEC, JAIGEL-2HR ($CHCl_3$/MeOH (9/1, v/v) as eluent) afforded title compound **dc2-Val-OH** (100 mg, 21%) as a white solid. ^{1}H NMR (400 MHz, CD_2Cl_2/$(CD_3)_2SO$ (19/1, v/v), 25 ºC) (for as-obtained **dc2-Val-OH**): δ 7.78 + 7.77 (s + s, 1H), 7.58-6.96 (m, 28H), 6.73 (s, 1H), 6.71 + 6.68 (s + s, 1H), 6.39-6.38 (m, 2H), 5.27-5.11 (m, 2H), 4.48-4.32 (m, 4H), 4.18 + 3.98 (t + t, J = 8.0 and 7.6 Hz, respectively, 1H), 3.90-3.63 (m, 8H), 3.58-3.55 (m, 1H), 3.42-3.19 (m, 7H), 2.66-0.72 (m, partially overlapping with the residual $(CD_3)(CD_2H)SO$ and H_2O signals), 0.58-0.52 (m, 1H), 0.27 (bs, 1H). ^{13}C NMR (100 MHz, CD_2Cl_2/$(CD_3)_2SO$ (9/1, v/v), 25 ºC) (for annealed **dc2-Val-OH**): δ 177.42, 176.14, 175.67, 175.40, 175.31, 175.02, 174.96, 174.91, 174.52, 172.94, 168.24, 168.20, 168.05, 157.17, 139.44, 138.89, 138.46, 138.25, 137.17, 135.50, 135.27, 134.81, 134.67, 128.94, 128.61, 128.56, 128.46, 128.29, 128.12, 127.84, 127.40, 127.26, 127.13, 126.87, 66.66, 60.45, 60.02, 59.31, 59.11, 58.32, 58.27, 58.22, 58.12, 57.99, 57.37, 57.14,

42.78, 42.72, 42.65, 42.53, 42.25, 41.92, 41.89, 41.53, 37.55, 37.36, 37.11, 36.13, 35.20, 34.85, 34.78, 34.49, 34.46, 34.29, 34.20, 34.14, 33.89, 32.22, 30.76, 29.97, 29.81, 29.65, 29.60, 29.50, 29.43, 28.85, 28.70, 28.51, 28.38, 28.27, 28.13, 28.01, 27.60, 25.99, 25.92, 25.73, 25.54, 25.34, 22.99, 22.72, 22.58, 22.51, 22.24, 22.02, 21.91, 21.85, 21.79, 21.63, 21.49, 19.74, 19.43. HRMS (ESI-TOF+): *m/z* calcd for ($M+Na^+$), 2122.1468; found, 2122.1512.

Supplementary Data

3. Molecular Design Strategy for Singly and Doubly Crosslinked (Stapled) α-Helical Peptides

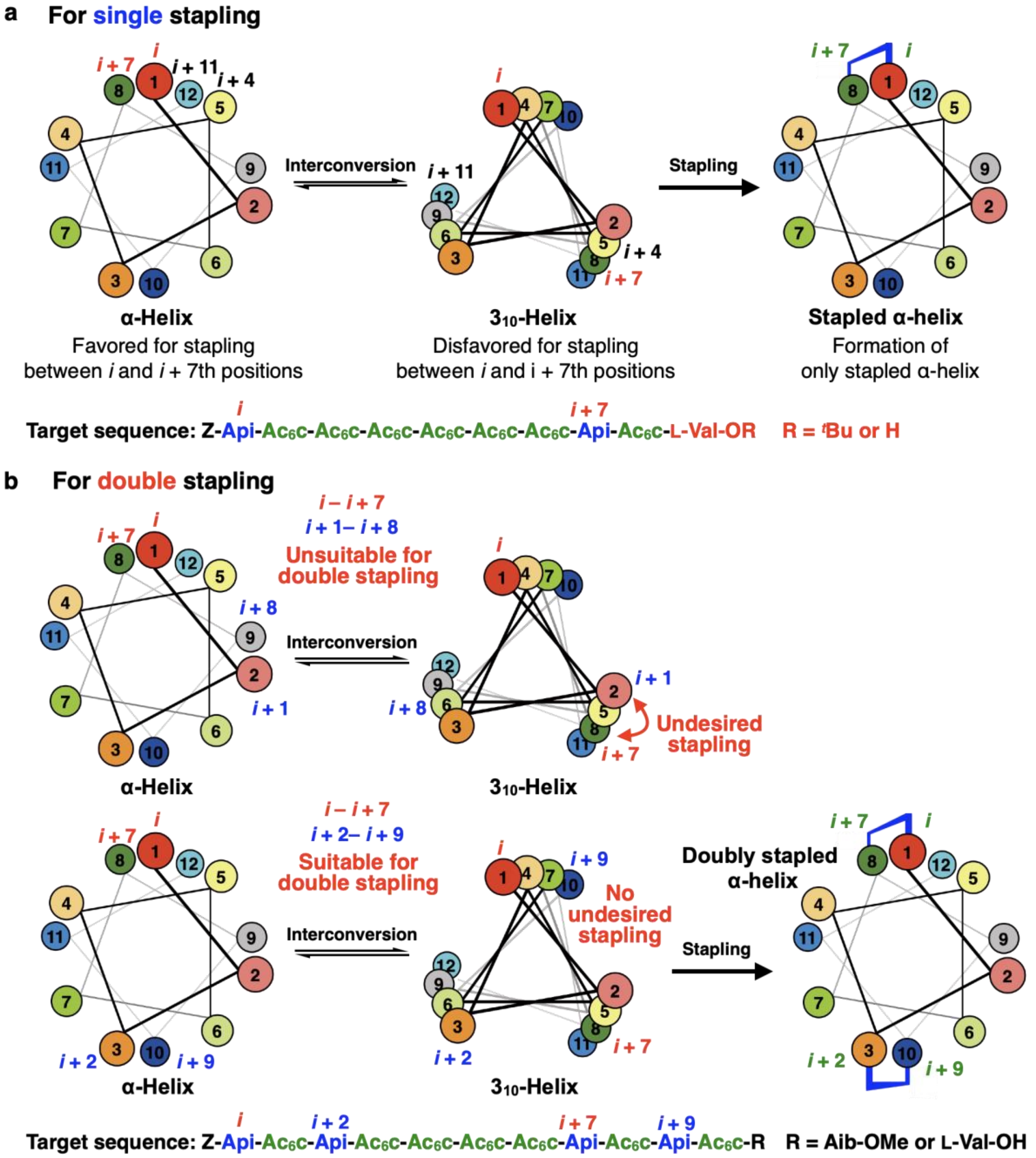

Supplementary Figure 5 | Molecular design strategy of stapled peptides. Schematic representations of interconversion between α- and 3_{10}-helical conformations depicted as helical wheel diagrams and subsequent single (**a**) and double (**b**) staple-mediated α-helix induction in oligopeptides with appropriate amino acid sequences. For (**b**), double stapling between i and i + 7 positions and between i + 1 and i + 8 positions in dynamic helical peptides may lead to undesired stapling between i + 1 and i + 7 positions, which are close to each other in the 3_{10}-helix. In contrast, such undesired stapling may not occur for double stapling between i and i + 7 positions and between i + 2 and i + 9 positions.

4. ESI-TOF Mass Spectra and HPLC Chromatograms of c1-Val-O^{t}Bu, c1-Val-OH, dc2-Val-OH and dc2-Aib-OMe

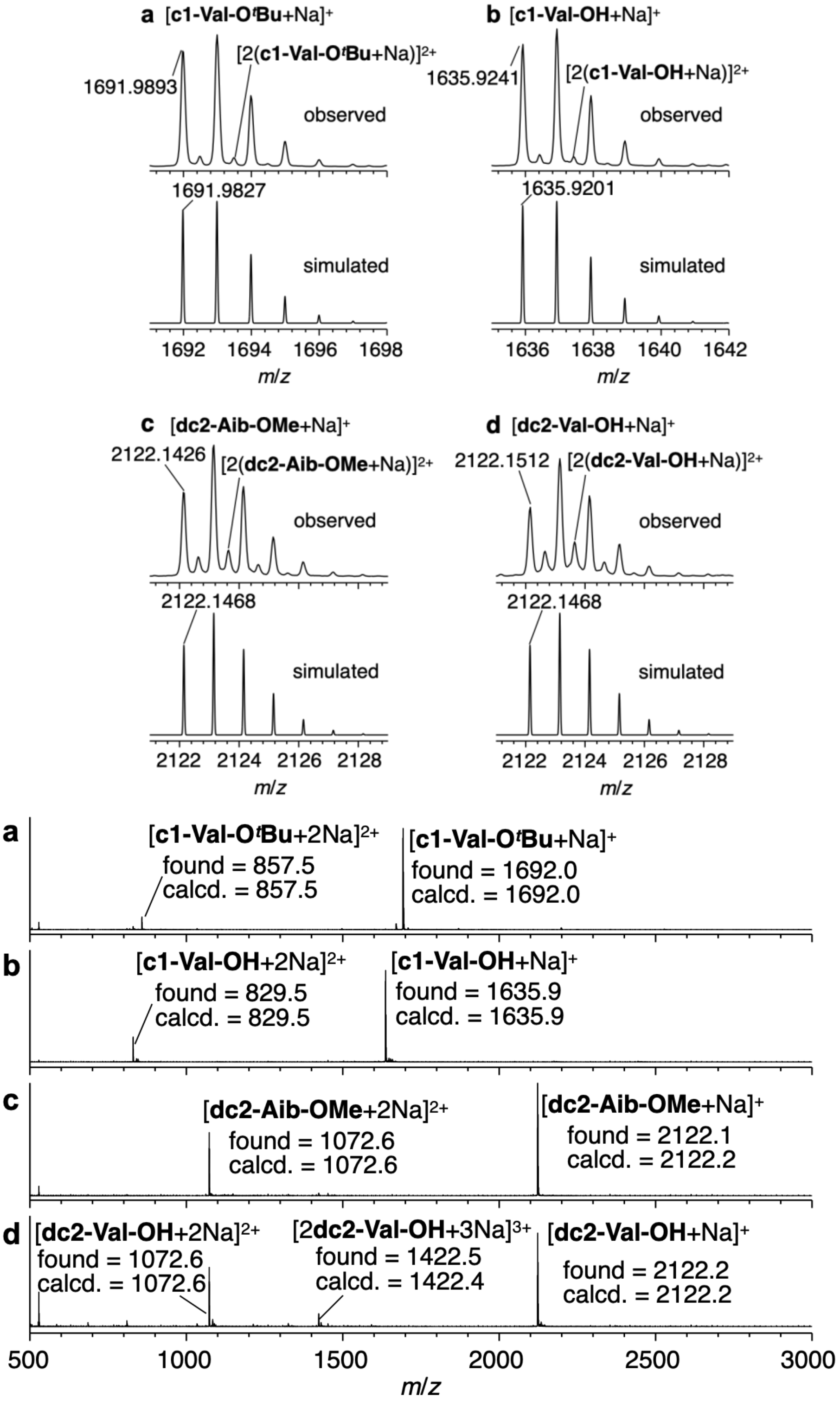

Supplementary Figure 6 | ESI-TOF mass spectra of the singly and doubly stapled peptides. Experimental (top) and simulated (bottom) electrospray ionization time-of-flight (ESI-TOF) mass spectra of **c1-Val-O^{t}Bu** (**a**), **c1-Val-OH** (**b**), **dc2-Aib-OMe** (**c**) and **dc2-Val-OH** (**d**). Full spectra are also shown.

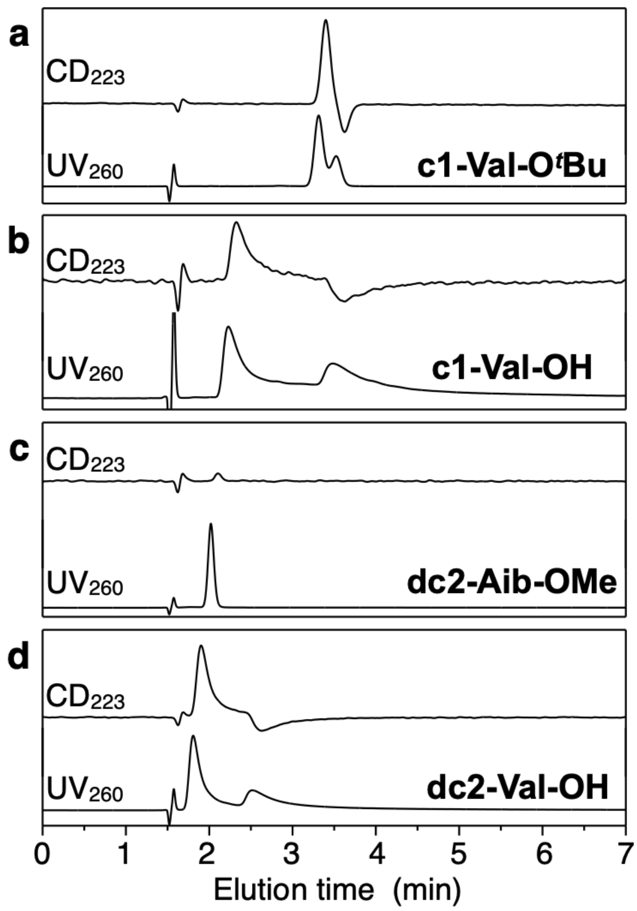

Supplementary Figure 7 | HPLC chromatograms of the singly and doubly stapled peptides. CD (λ = 223 nm) and UV (λ = 260 nm) detected HPLC chromatograms of **c1-Val-O*t*Bu** (**a**), **c1-Val-OH** (**b**), **dc2-Aib-OMe** (**c**) and **dc2-Val-OH** (**d**). HPLC conditions: column, COSMOSIL 5C$_{18}$-MS-II (Nacalai Tesque, 0.46 (i.d.) × 25 cm); eluent, MeOH; flow rate, 2.0 mL/min; column temperature, 25 ºC.

5. Theoretical Studies on the Structures of Stapled Peptides c1-Val-OH and **dc2-Aib-OMe**

The molecular modeling was performed on a Windows 11 PC with the ArgusLab software[6]. The initial structures of **c1-Val-OH** and **dc2-Aib-OMe** were constructed according to the following procedures: the peptide backbones were set to be a standard (*P*)-α-helix ((ϕ, ψ, ω) = (–57°, –47°, 180°))[7,8] or a standard (*P*)-3_{10}-helix ((ϕ, ψ, ω) = (–60°, –30°, 180°))[9], except for the C-terminal Aib residue of **dc2-Aib-OMe** that took the opposite handedness ((ϕ, ψ, ω) = (+57°, +47°, 180°)) for (*P*)-α-helix and ((ϕ, ψ, ω) = (+60°, +30°, 180°)) for (*P*)-3_{10}-helix, respectively, because the helix inversion at the C-terminus is often found in the analogous peptides[10]. All the amide NH groups of the Api residues were placed at the axial position of the piperidine ring in the initial structures of the α-helical **c1-Val-OH** and **dc2-Aib-OMe**. The amide NH groups of the Api(1) residue for the 3_{10}-helical **c1-Val-OH** and of the Api(1) and Api(3) residues for the 3_{10}-helical **dc2-Aib-OMe** were placed at the equatorial position of the piperidine ring. These treatments were necessary to construct the initial stapled 3_{10}-helical structures. The biphenyl-based crosslinker moieties of these initial structures were selectively geometry-optimized by molecular mechanics calculations (Universal force field (UFF) in ArgusLab software). The resulting structures were then fully optimized by the semi-empirical molecular orbital (MO) calculations (PM6 method[11] in MOPAC2016[12]) and the geometries were further refined by density functional theory (DFT) calculations using the dispersion corrected B3LYP (B3LYP-D3)[13] functional in *Gaussian* 16 software (Gaussian, Inc., Pittsburgh, PA)[14].

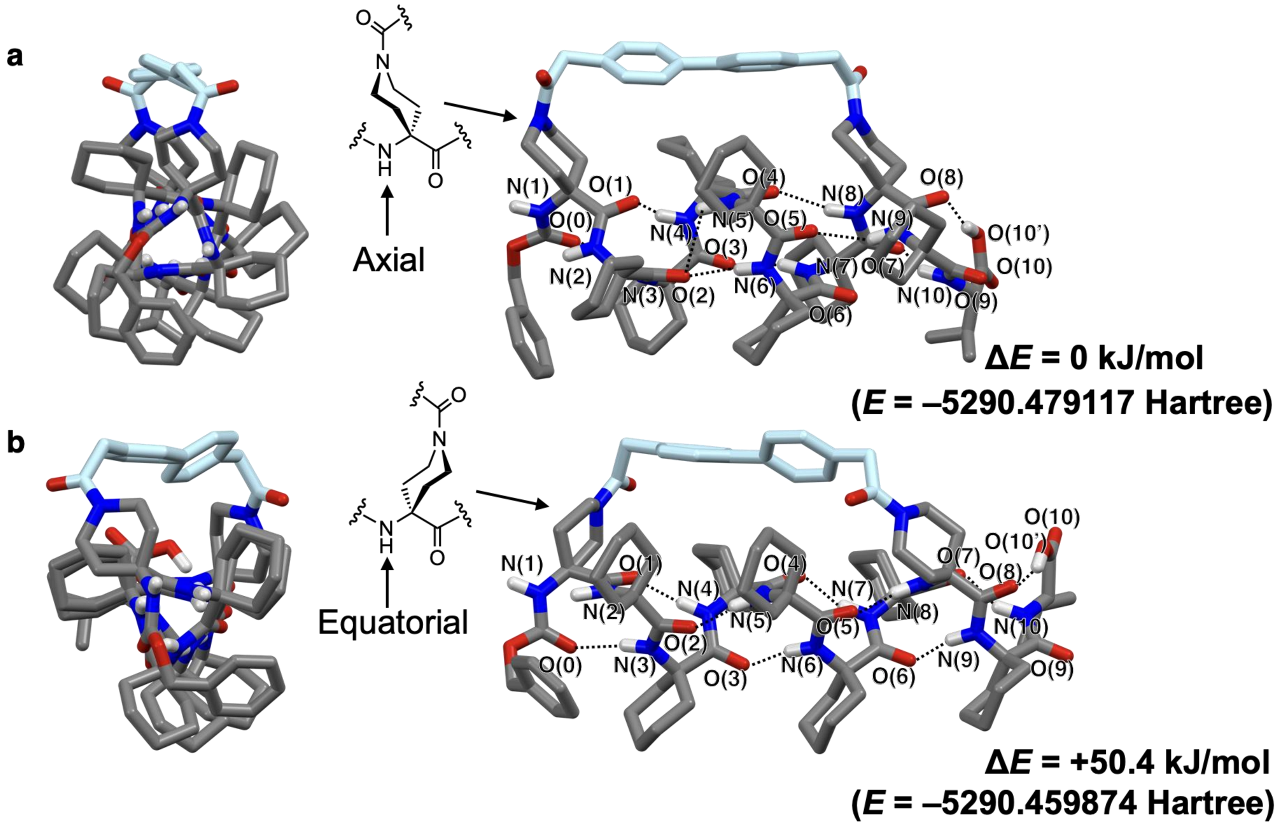

Supplementary Figure 8 | DFT calculated structures of c1-Val-OH. The energy-minimized structures of (*P*)-α-helical **c1-Val-OH** (**a**) and (*P*)-3_{10}-helical **c1-Val-OH** (**b**) obtained by DFT calculations (top (left) and side (right) views). All of the hydrogen atoms except for the amide and C-terminal carboxy protons are omitted for clarity. The energy difference (Δ*E*) values are also shown. The dihedral angles (ϕ, ψ and ω) and hydrogen-bonding parameters of the α- and 3_{10}-helical **c1-Val-OH** are summarized in Supplementary Tables 1–4. The structure shown in (**a**) is identical to that shown in Fig. 3a.

Supplementary Table 1. Conformational angles (º) of (*P*)-α-helical **c1-Val-OH** obtained by DFT calculation

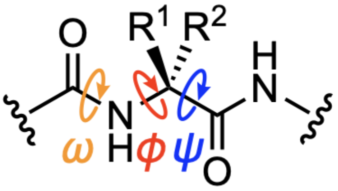

Residue (No.)	ϕ	ψ	ω
Api (1)	–57	–33	–170
Ac6c (2)	–49	–44	–178
Api (3)	–53	–51	–172
Ac6c (4)	–56	–51	–173
Ac6c (5)	–57	–51	–175
Ac6c (6)	–55	–51	–176
Ac6c (7)	–54	–50	–175
Api (8)	–58	–43	–171
Ac6c (9)	–57	–23	–177
L-Val (10)	–123	33	175
|Average| (1)-(9)	55	44	174

Supplementary Table 2. Parameters of the hydrogen bonds (Å, º) of (*P*)-α-helical **c1-Val-OH** obtained by DFT calculation

Intramolecular H-Bond Donor	Intramolecular H-Bond Acceptor	N···O	H···O	N-H···O	C-O···H
N(1)	–				
N(2)	–				
N(3)	O(0)	2.976	2.014	157	127
N(4)	O(1)	3.192	2.362	138	113
N(5)	O(2)	3.202	2.565	121	100
N(6)	O(2)	3.201	2.215	163	157
N(7)	O(3)	3.111	2.119	165	156
N(8)	O(4)	3.157	2.180	161	154
N(9)	O(5)	3.138	2.160	161	158
N(10)	O(7)	3.184	2.362	138	99

Supplementary Table 3. Conformational angles (º) of (*P*)-3_{10}-helical **c1-Val-OH** obtained by DFT calculation

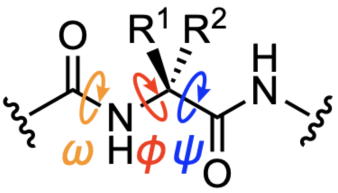

Residue (No.)	ϕ	ψ	ω
Api (1)	–55	–37	–169.50
Ac_6c (2)	–62	–11	–167.06
Api (3)	–48	–34	168.53
Ac_6c (4)	–59	–18	–176.95
Ac_6c (5)	–53	–29	176.53
Ac_6c (6)	–53	–30	–178.73
Ac_6c (7)	–53	–27	–179.17
Api (8)	–58	–29	–176.64
Ac_6c (9)	–56	–21	176.19
L-Val (10)	–122	29	179.72
|Average| (1)-(9)	55	26	174

Supplementary Table 4. Parameters of the hydrogen bonds (Å, º) of (*P*)-3_{10}-helical **c1-Val-OH** obtained by DFT calculation

Intramolecular H-Bond Donor	Intramolecular H-Bond Acceptor	N···O	H···O	N-H···O	C-O···H
N(1)	–				
N(2)	–				
N(3)	O(0)	3.210	2.201	173	133
N(4)	O(1)	3.033	2.019	175	137
N(5)	O(2)	2.950	1.945	169	123
N(6)	O(3)	3.068	2.057	172	131
N(7)	O(4)	2.989	1.988	167	126
N(8)	O(5)	2.963	1.953	171	129
N(9)	O(6)	3.072	2.078	165	127
N(10)	O(7)	2.831	1.868	157	119

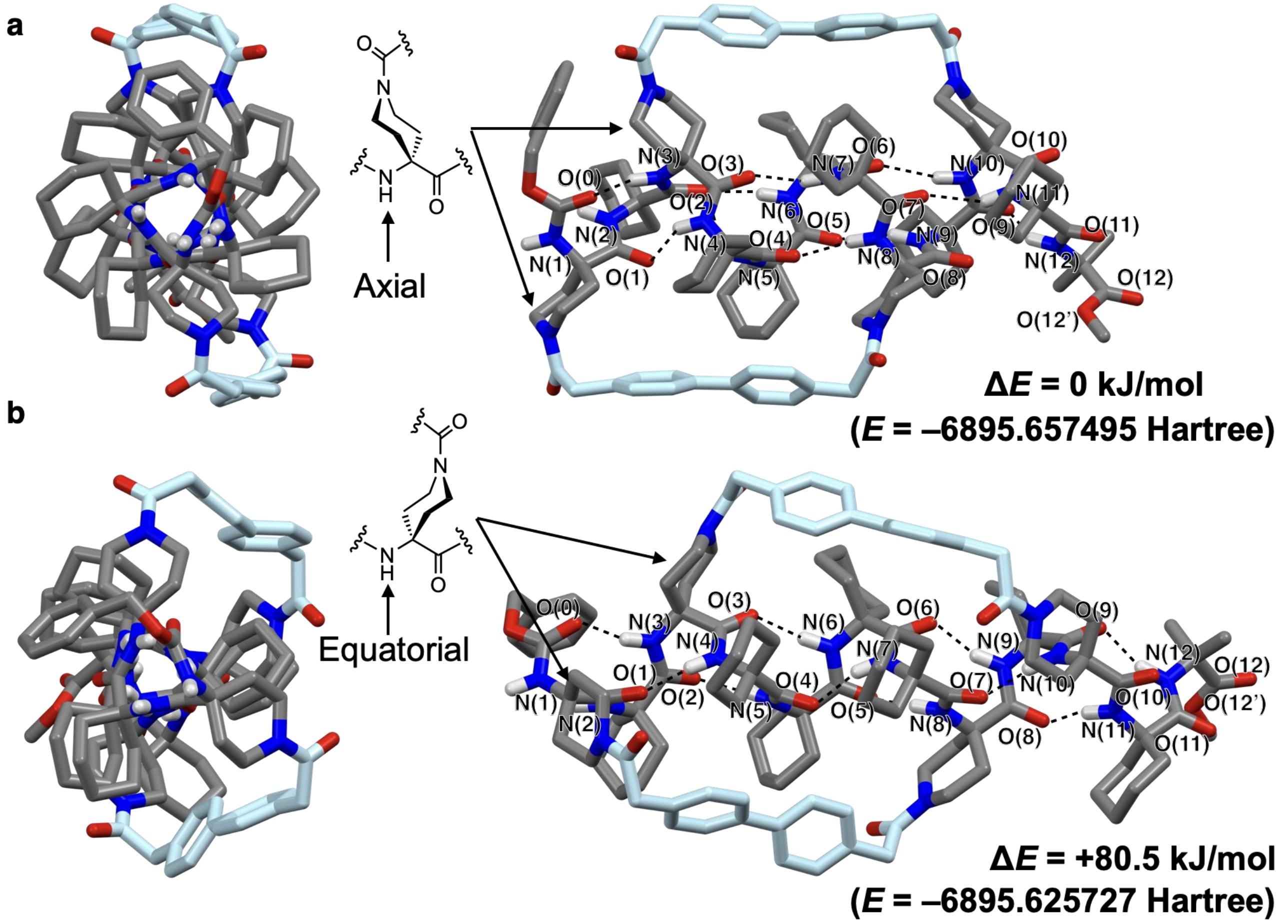

Supplementary Figure 9 | DFT calculated structures of dc2-Aib-OMe. The energy-minimized structures of (P)-α-helical **dc2-Aib-OMe** (**a**) and (P)-3_{10}-helical **dc2-Aib-OMe** (**b**) obtained by DFT calculations (top (left panel) and side (right panel) views). All of the hydrogen atoms except for the amide protons are omitted for clarity. The energy difference (ΔE) values are also shown. The dihedral angles (ϕ, ψ and ω) and hydrogen-bonding parameters of the α- and 3_{10}-helical **dc2-Aib-OMe** are summarized in Supplementary Tables 5–8.

Supplementary Table 5. Conformational angles (°) of (*P*)-α-helical **dc2-Aib-OMe** obtained by DFT calculation

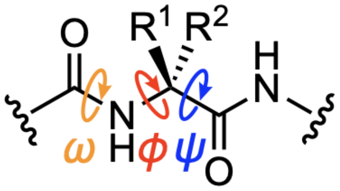

Residue (No.)	ϕ	ψ	ω
Api (1)	–58	–34	–170
Ac6c (2)	–50	–41	–177
Api (3)	–57	–47	–169
Ac6c (4)	–54	–50	–175
Ac6c (5)	–57	–52	–176
Ac6c (6)	–53	–53	–177
Ac6c (7)	–54	–50	–174
Api (8)	–56	–50	–175
Ac6c (9)	–56	–45	–174
Api (10)	–59	–45	–174
Ac6c (11)	–62	–32	–177
Aib (12)	50	42	176
|Average| (1)-(11)	55	45	175

Supplementary Table 6. Parameters of the hydrogen bonds (Å, °) of (*P*)-α-helical **dc2-Aib-OMe** obtained by DFT calculation

Intramolecular H-bond Donor	Intramolecular H-bond Acceptor	N···O	H···O	N-H···O	C-O···H
N(1)	–				
N(2)	–				
N(3)	O(0)	2.976	2.014	157	127
N(4)	O(1)	3.192	2.362	138	113
N(5)	O(2)	3.202	2.565	121	100
N(6)	O(2)	3.201	2.215	163	157
N(7)	O(3)	3.111	2.119	165	156
N(8)	O(4)	3.157	2.180	161	154
N(9)	O(5)	3.138	2.160	161	158
N(10)	O(6)	3.218	2.267	156	155
N(11)	O(7)	3.333	2.412	151	152
N(12)	O(9)	3.184	2.362	138	99

Supplementary Table 7. Conformational angles (º) of (*P*)-3_{10}-helical **dc2-Aib-OMe** obtained by DFT calculation

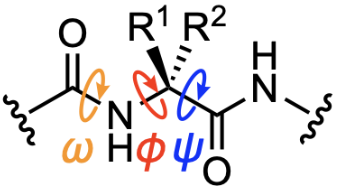

Residue (No.)	ϕ	ψ	ω
Api (1)	–56	–39	–172.33
Ac_6c (2)	–62	–7.1	–172.40
Api (3)	–43	–43	163.71
Ac_6c (4)	–62	–7.9	–170.10
Ac_6c (5)	–50	–31	168.01
Ac_6c (6)	–55	–27	–178.68
Ac_6c (7)	–53	–31	179.98
Api (8)	–55	–31	174.00
Ac_6c (9)	–57	–23	–178.22
Api (10)	–56	–33	–179.06
Ac_6c (11)	–62	–25	177.94
Aib (12)	49	42	–179.88
|Average| (1)-(11)	56	27	175

Supplementary Table 8. Parameters of the hydrogen bonds (Å, º) of (*P*)-3_{10}-helical **dc2-Aib-OMe** obtained by DFT calculation

Intramolecular H-bond Donor	Intramolecular H-bond Acceptor	N···O	H···O	N-H···O	C-O···H
N(1)	–				
N(2)	–				
N(3)	O(0)	3.011	2.007	129	129
N(4)	O(1)	2.995	1.980	174	135
N(5)	O(2)	3.062	2.054	171	122
N(6)	O(3)	3.032	2.016	175	136
N(7)	O(4)	2.922	1.927	165	121
N(8)	O(5)	2.994	1.985	170	129
N(9)	O(6)	3.071	2.078	164	125
N(10)	O(7)	2.989	1.981	170	127
N(11)	O(8)	3.117	2.127	164	125
N(12)	O(9)	3.000	2.011	164	116

6. X-ray Crystallographic Analysis of dc2-Aib-OH

Crystals of **dc2-Aib-OH** suitable for single-crystal X-ray diffraction were obtained by slow evaporation of a $CDCl_3/(CD_3)_2SO$ (~20/1, v/v) mixed solution of **dc2-Aib-OMe** via unexpected hydrolysis of the methyl ester group of the C-terminal Aib residue at room temperature. A colorless single crystal with dimensions 0.63 × 0.51 × 0.26 mm^3 was selected for intensity measurements. The X-ray diffraction data for **dc2-Aib-OH** were collected on a Bruker Venture D8 diffractometer with Cu-Kα radiation (λ = 1.54178 Å) at 100 K. The data were corrected for Lorentz and polarization factors and for absorption by semiempirical methods based on symmetry-equivalent and repeated reflections. The structure was solved by direct methods (SHELXD[15]) and refined by full-matrix least squares on F^2 using SHELXL 2014[16]. All non-hydrogen atoms were refined anisotropically. All hydrogen atoms were calculated geometrically and refined using the riding models. The crystal data are summarized in Supplementary Table 9.

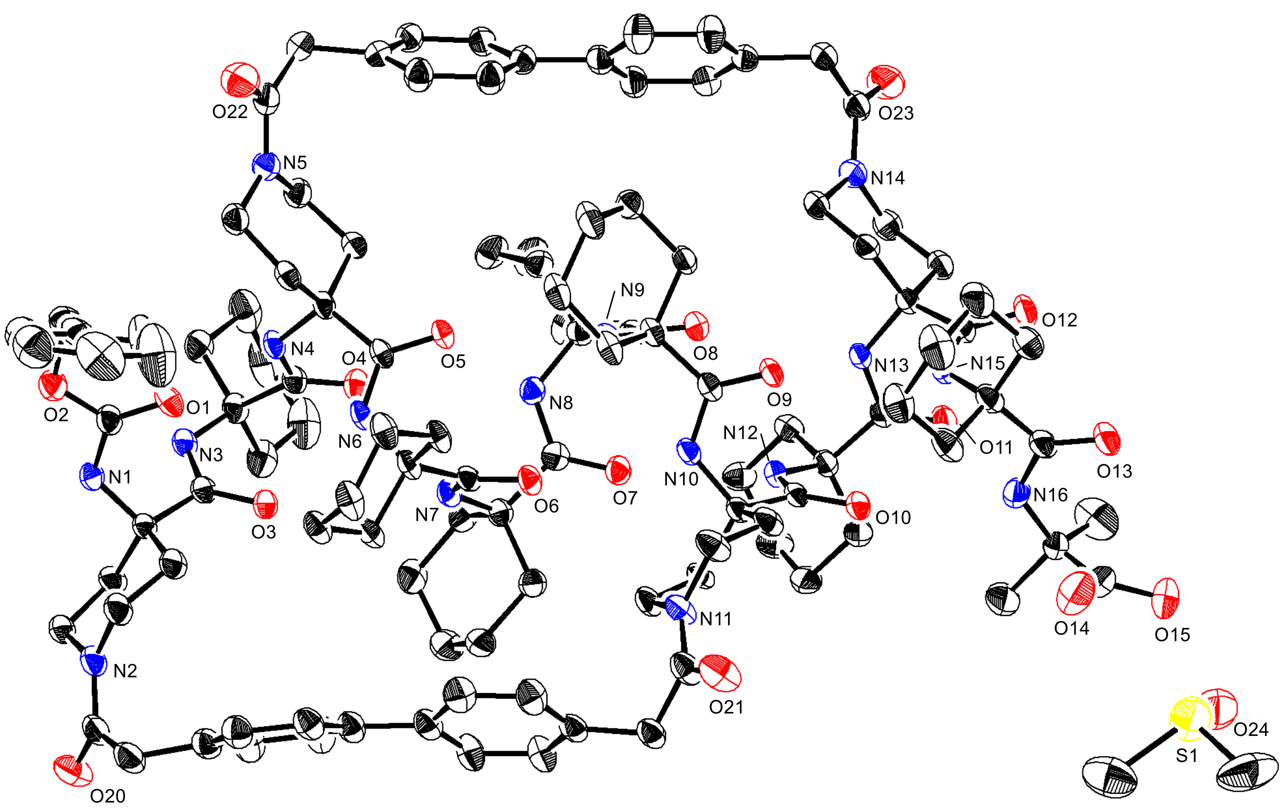

Supplementary Figure 10 | X-ray crystal structure of dc2-Aib-OH. ORTEP drawing of the crystal structure of the (*P*)-α-helical **dc2-Aib-OH** with thermal ellipsoids at 50% probability. Hydrogen atoms, solvent molecules except for the hydrogen-bonded DMSO, and minor parts of the disordered atoms are omitted for clarity.

Supplementary Table 9. Crystallographic data for **dc2-Aib-OH**•10$CHCl_3$•0.75Me_2SO (CCDC-2261608)

Formula	$C_{128.5}H_{166.5}Cl_{30}N_{16}O_{19.75}S_{0.75}$ (3338.81)
Crystal system	monoclinic
Space group	$P2_1/c$
a (Å)	18.7957(5)
b (Å)	32.3823(9)
c (Å)	27.1257(8)
α (deg)	90
β (deg)	101.4717(12)
γ (deg)	90
V (Å^3)	16180.2(8)
Z	4
D_{calcd} (g cm^{-3})	1.371
Collected reflections	324856
Unique reflections	29698
R_{int}	0.0937
$2\theta_{max}$ (deg)	137.358
F_{000}	6918
μ(CuKα) (mm^{-1})	5.225
Limiting indices	$-22 \leq h \leq 22$<br>$-39 \leq k \leq 39$<br>$-32 \leq l \leq 32$
Restraints/parameters	4349/2785
Goodness of fit (F^2)	1.091
$R1$ ($I>2\sigma(I)$)	0.1024
$wR2$ ($I>2\sigma(I)$)	0.2739
$R1$ (all data)	0.1068
$wR2$ (all data)	0.2773

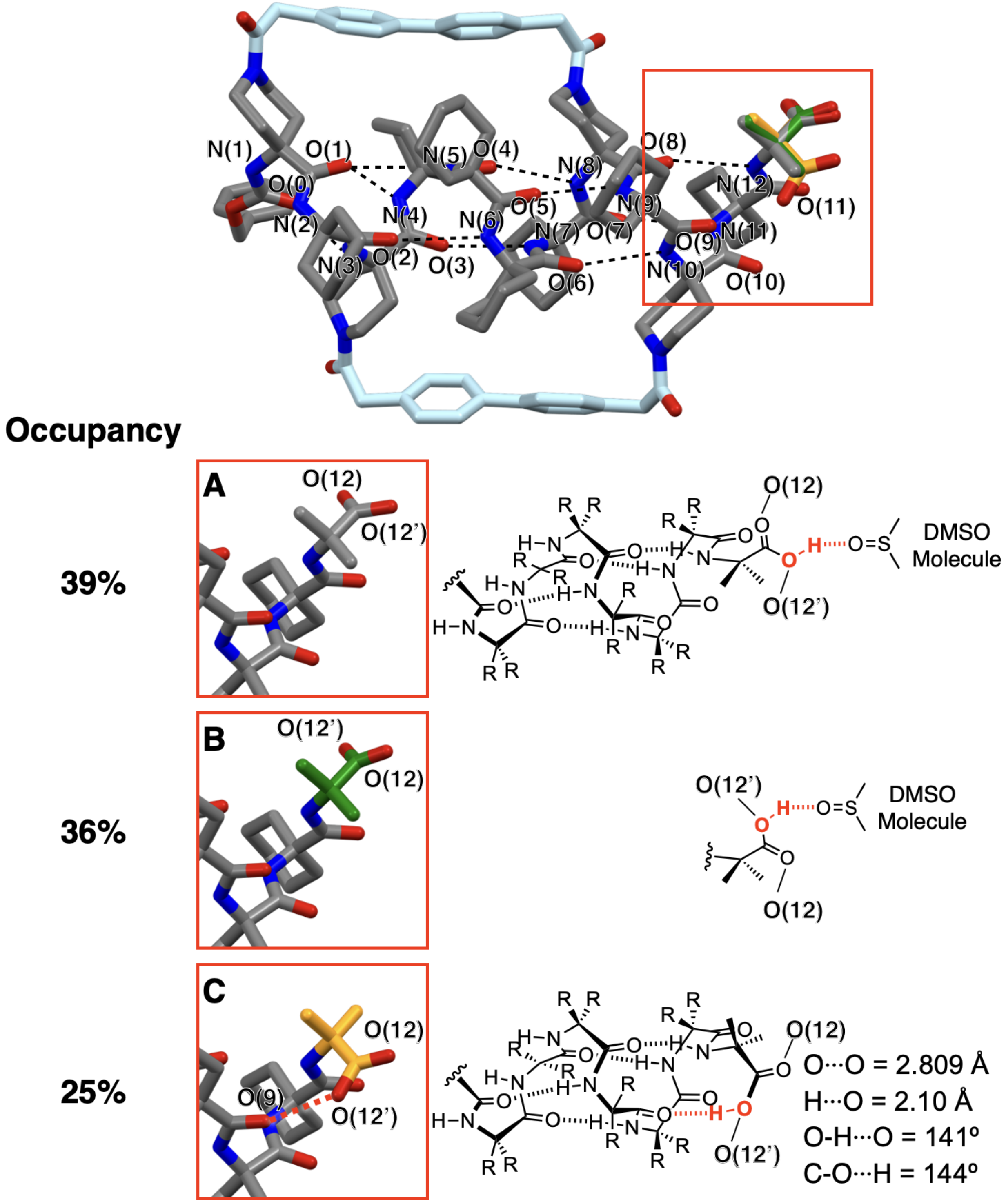

Supplementary Figure 11 | X-ray crystal structure of dc2-Aib-OH with disordered components. The X-ray crystal structure of (*P*)-α-helical **dc2-Aib-OH** with the three superimposed disordered components (**A** (gray), **B** (green) and **C** (orange) with occupancies of 39, 36 and 25%, respectively) at the C-terminal Aib residue. The disordered components are highlighted with red square boxes. Only the (*P*)-helix is shown. All of the hydrogen atoms and solvent molecules are omitted for clarity. The dihedral angles (ϕ, ψ and ω) and hydrogen-bonding parameters of **dc2-Aib-OH** are summarized in Supplementary Tables 10 and 11.

Supplementary Table 10. Conformational angles (°) of **dc2-Aib-OH** for the (*P*) conformation

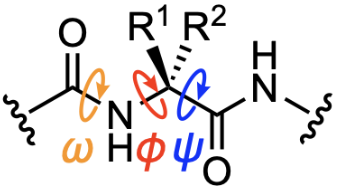

Residue (No.)	*ϕ*	*ψ*	*ω*
Api (1)	–54	–44	–171
Ac6c (2)	–49	–48	–175
Api (3)	–58	–44	–170
Ac6c (4)	–54	–48	–175
Ac6c (5)	–56	–52	–175
Ac6c (6)	–54	–50	–176
Ac6c (7)	–56	–50	–174
Api (8)	–58	–48	–174
Ac6c (9)	–55	–45	–175
Api (10)	–59	–47	–175
Ac6c (11)	–61	–54	–177
Aib (12)[a]	+55 (disorder-A)<br>+55 (disorder-B)<br>–75 (disorder-C)	–141 (disorder-A)<br>+27 (disorder-B)<br>–33 (disorder-C)	–177 (disorder-A)<br>–173 (disorder-B)<br>–164 (disorder-C)
|Average| (1)-(11)	56	48	174

[a] The disordered C-terminal Aib residue adopts three different conformations with occupancies of 39% for A, 36% for B and 25% for C. For these structures, see Supplementary Figure 11.

Supplementary Table 11. Parameters of the hydrogen bonds (Å, º) of **dc2-Aib-OH**

Intramolecular H-bond Donor	Intramolecular H-bond Acceptor	N···O	H···O	N-H···O	C-O···H
N(1)	–				
N(2)	–				
N(3)	O(0)	2.980	2.30	134	120
N(4)	O(1)	3.083	2.49	125	111
N(5)	O(1)	3.572	2.73	161	158
N(6)	O(2)	3.420	2.56	166	158
N(7)	O(3)	3.188	2.32	167	157
N(8)	O(4)	3.130	2.27	167	158
N(9)	O(5)	3.104	2.24	168	161
N(10)	O(6)	3.229	2.39	160	156
N(11)	O(7)	3.332	2.48	163	157
N(12)	O(8)	3.102	2.22	174	164

7. Conformational Analysis of c1-Val-OH by ^1H-^1H NOESY and VT-NMR Spectroscopy

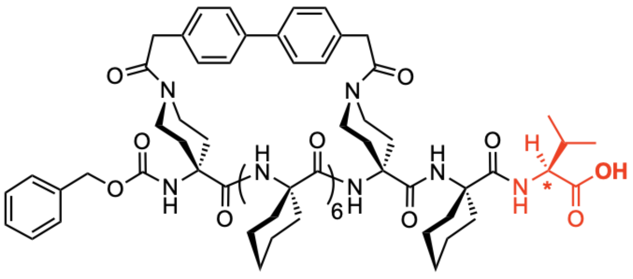

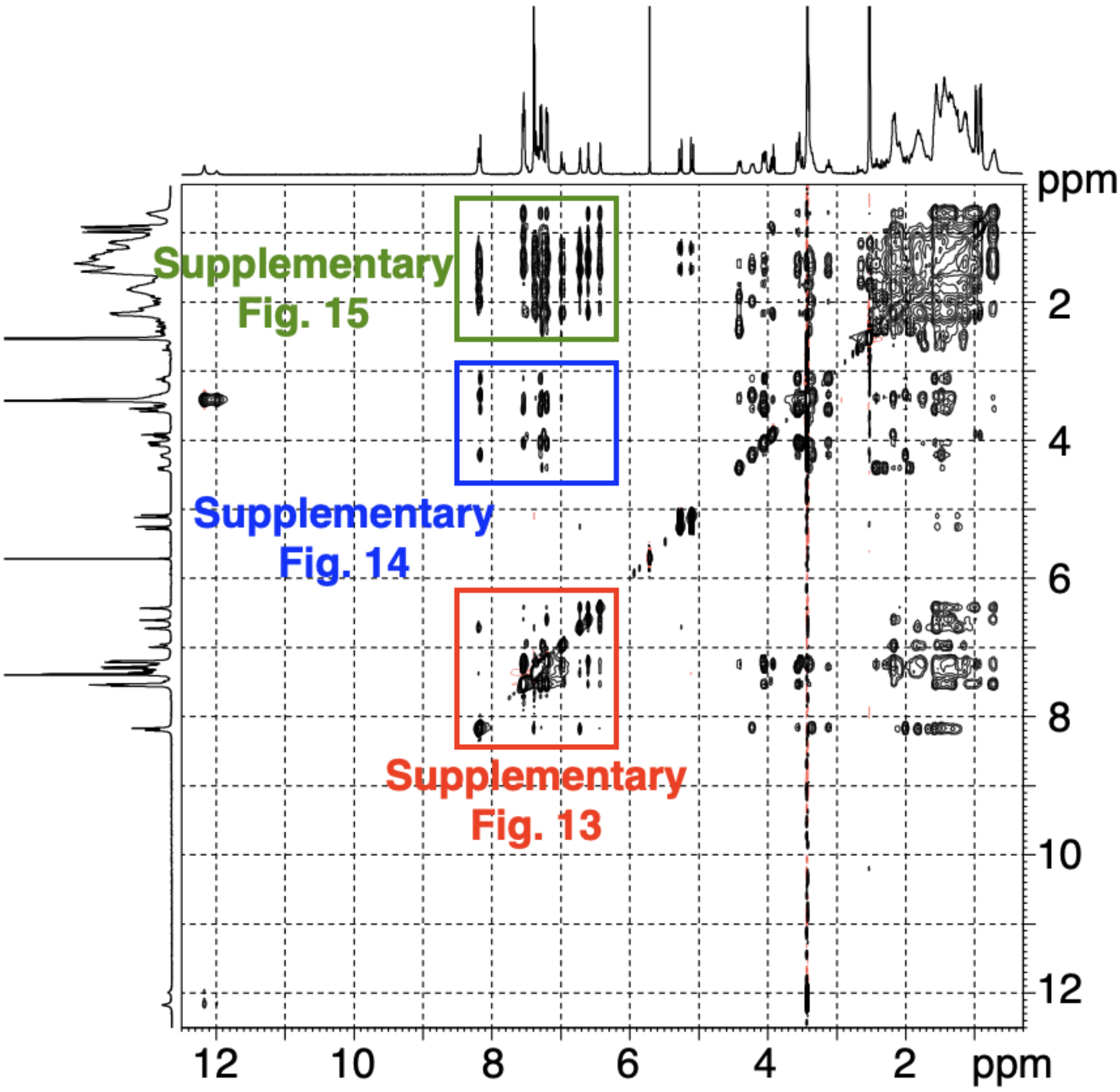

Supplementary Figure 12 | NOESY spectrum of c1-Val-OH in $(CD_3)_2SO/CD_2Cl_2$ (4/1, v/v). ^1H-^1H NOESY (400 MHz, $(CD_3)_2SO/CD_2Cl_2$ (4/1, v/v), 3.0 mM) spectrum of **c1-Val-OH** at 278 K: mixing time = 500 msec.

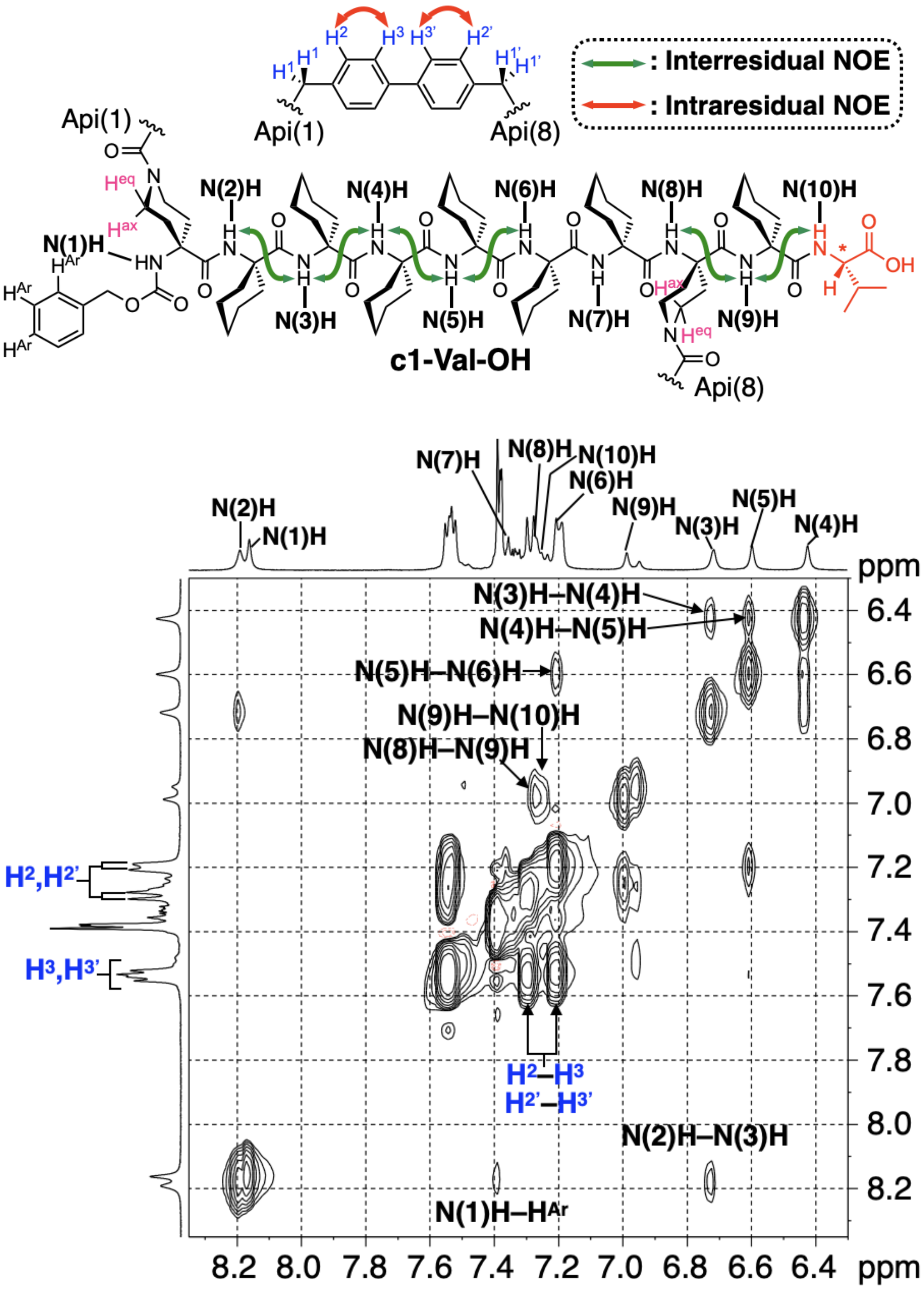

Supplementary Figure 13 | NOESY spectrum of c1-Val-OH in $(CD_3)_2SO/CD_2Cl_2$ (4/1, v/v). Partial ^1H-^1H NOESY (400 MHz, $(CD_3)_2SO/CD_2Cl_2$ (4/1, v/v), 3.0 mM) spectrum of **c1-Val-OH** at 278 K: mixing time = 500 msec.

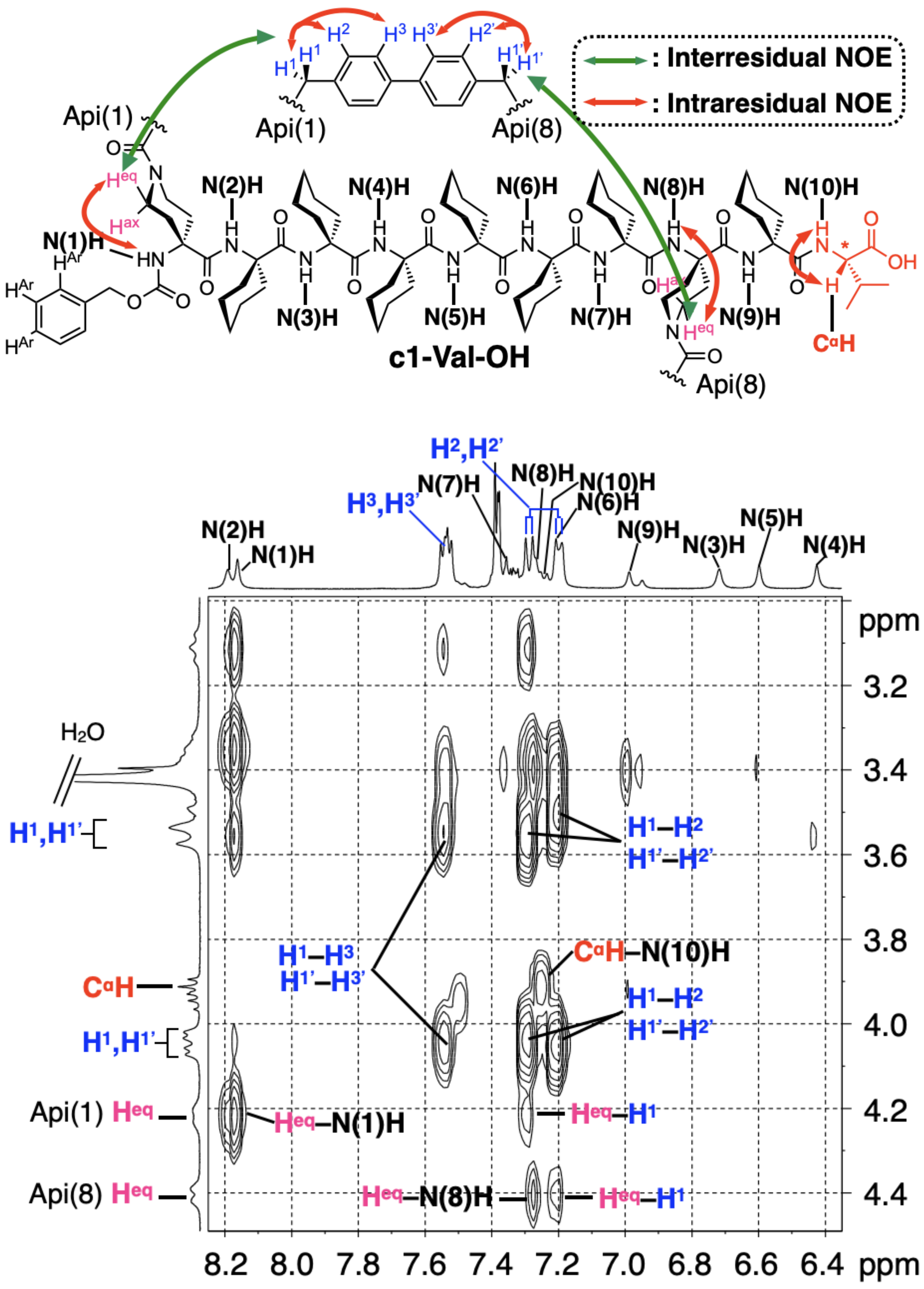

Supplementary Figure 14 | NOESY spectrum of c1-Val-OH in $(CD_3)_2SO/CD_2Cl_2$ (4/1, v/v). Partial ^1H-^1H NOESY (400 MHz, $(CD_3)_2SO/CD_2Cl_2$ (4/1, v/v), 3.0 mM) spectrum of **c1-Val-OH** at 278 K: mixing time = 500 msec.

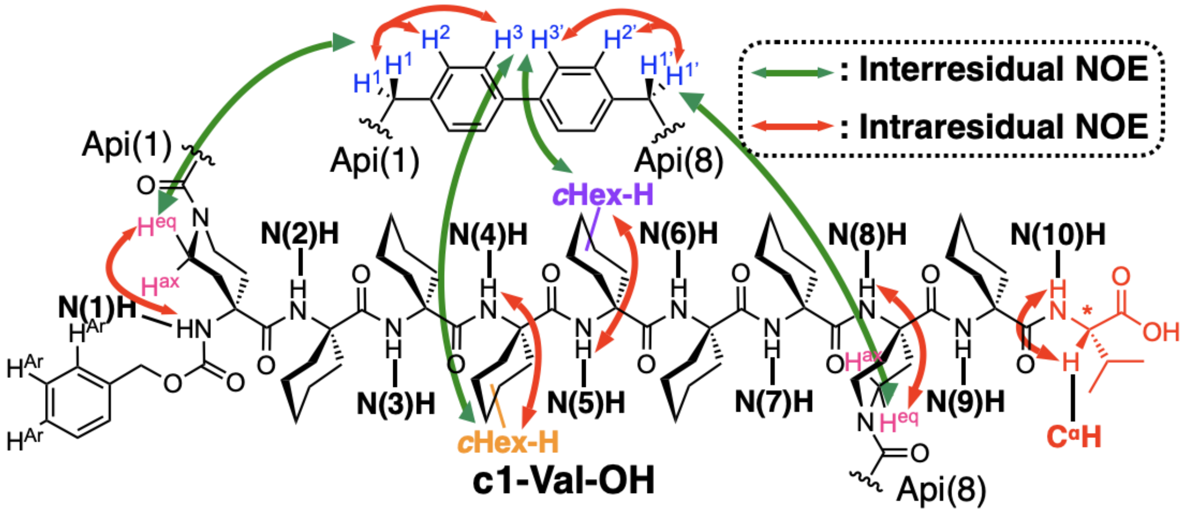

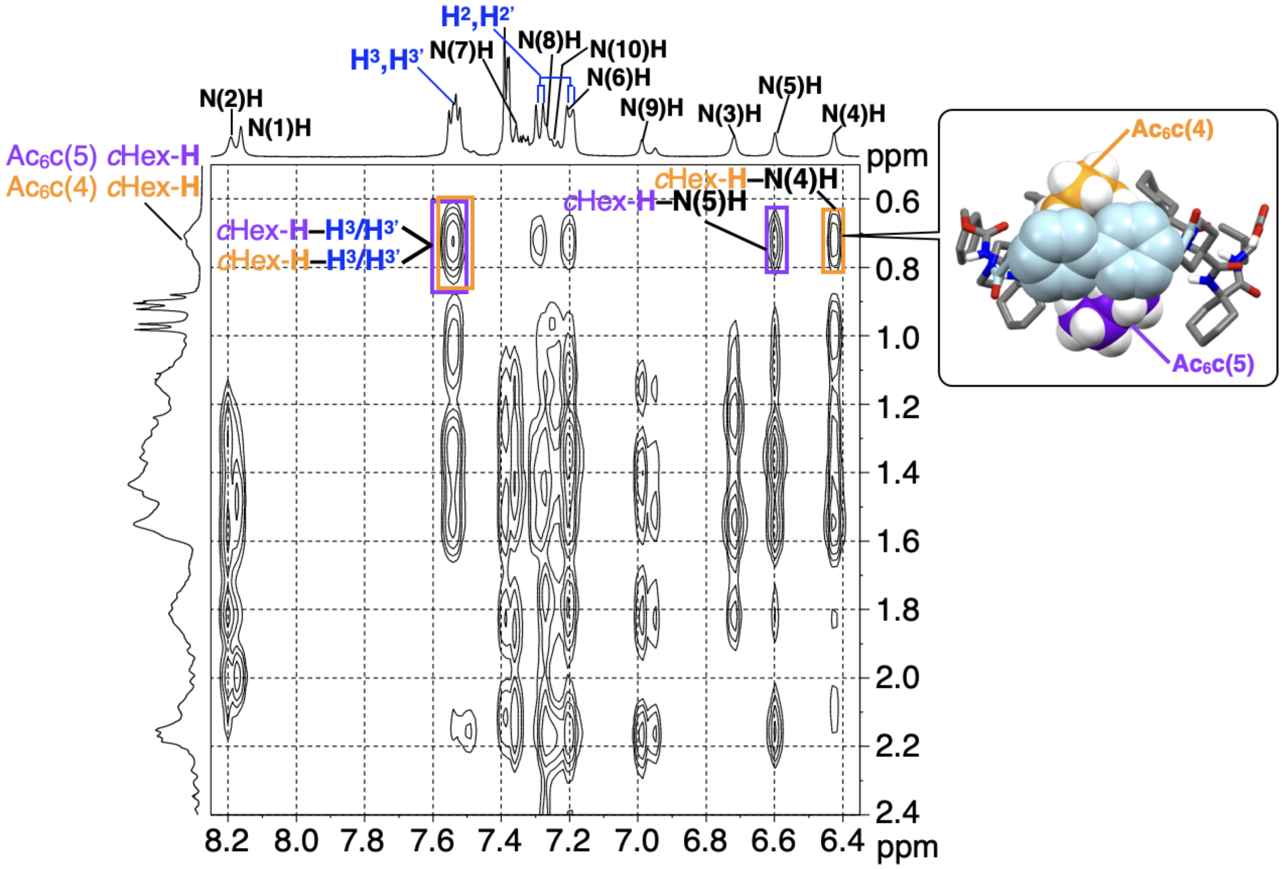

Supplementary Figure 15 | NOESY spectrum of c1-Val-OH in $(CD_3)_2SO/CD_2Cl_2$ (4/1, v/v). Partial ^{1}H-^{1}H NOESY (400 MHz, $(CD_3)_2SO/CD_2Cl_2$ (4/1, v/v), 3.0 mM) spectrum of **c1-Val-OH** at 278 K: mixing time = 500 msec. DFT-calculated structure of (*P*)-**c1-Val-OH** is also shown. The cyclohexyl side chains of the $Ac_6c(4)$ and $Ac_6c(5)$ residues and the biphenyl moiety are highlighted as a space-filling model.

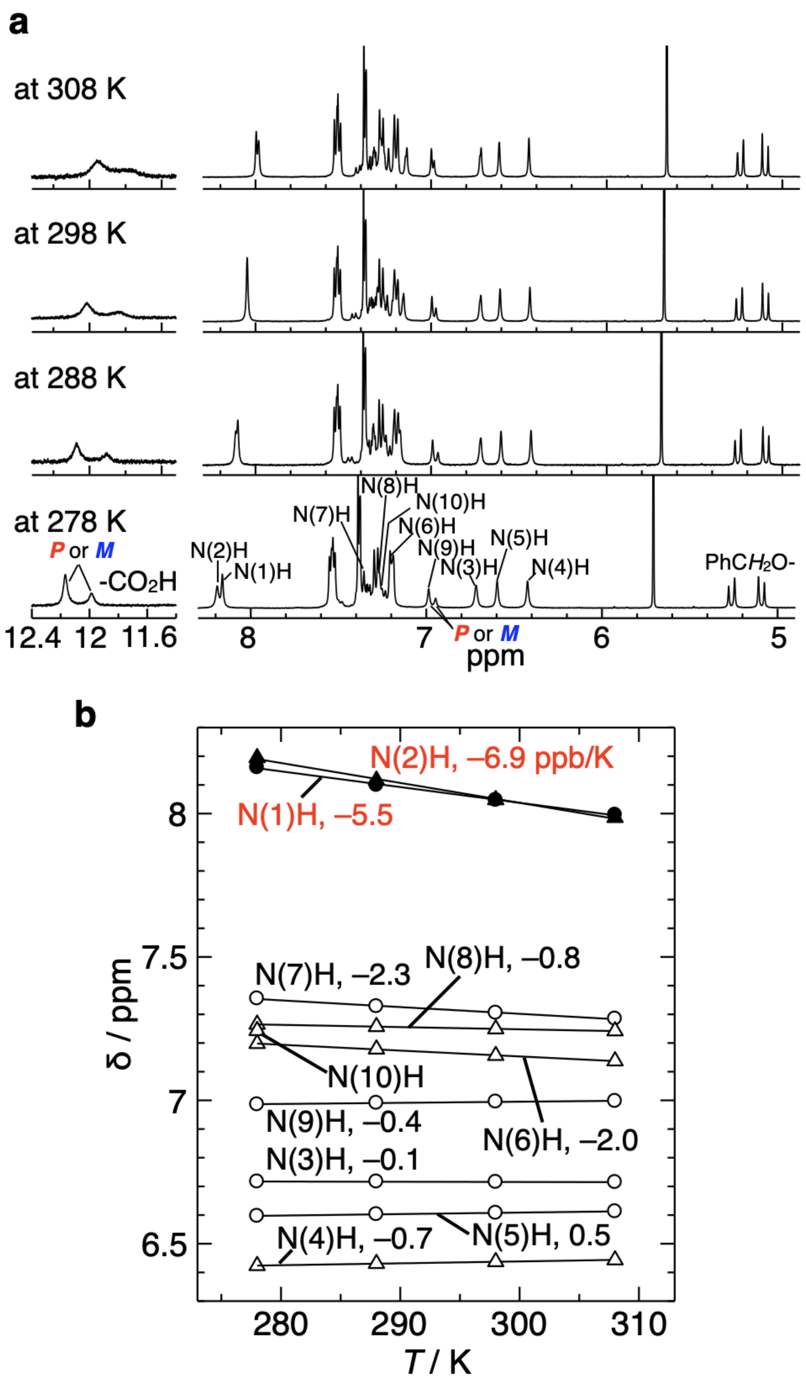

Supplementary Figure 16 | VT-^{1}H NMR spectra of c1-Val-OH in $(CD_3)_2SO/CD_2Cl_2$ (4/1, v/v). a, Temperature-dependent ^{1}H NMR (400 MHz, $(CD_3)_2SO/CD_2Cl_2$ (4/1, v/v), 3.0 mM) spectral changes of **c1-Val-OH**. **b**, Temperature dependence on the NH chemical shifts of **c1-Val-OH** in $(CD_3)_2SO/CD_2Cl_2$ (4/1, v/v) in 400 MHz ^{1}H NMR spectra. The number in parentheses indicates the residue numbers of the amide NH protons from the N-terminus. For the NH signal assignment, see Supplementary Figures 12–15. Temperature coefficients (ppb/K) of each amide NH proton signal, except for the N(10)H, are also shown. Temperature coefficients (ppb/K) of the N(10)H proton signal could not be determined due to signal overlap.

8. Conformational Analysis of dc2-Aib-OMe by ^{1}H-^{1}H NOESY and VT-NMR

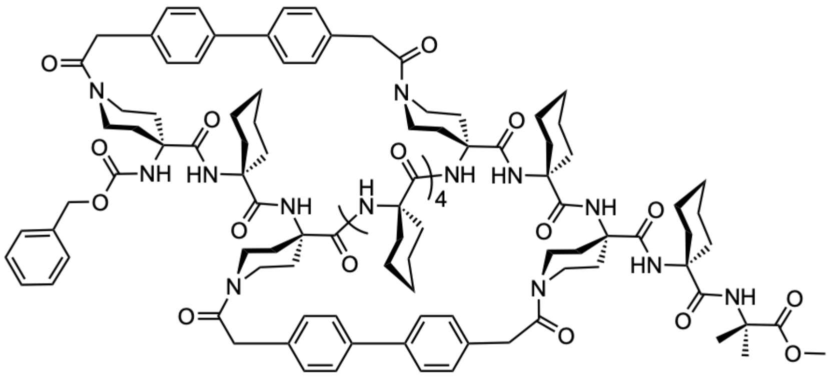

dc2-Aib-OMe

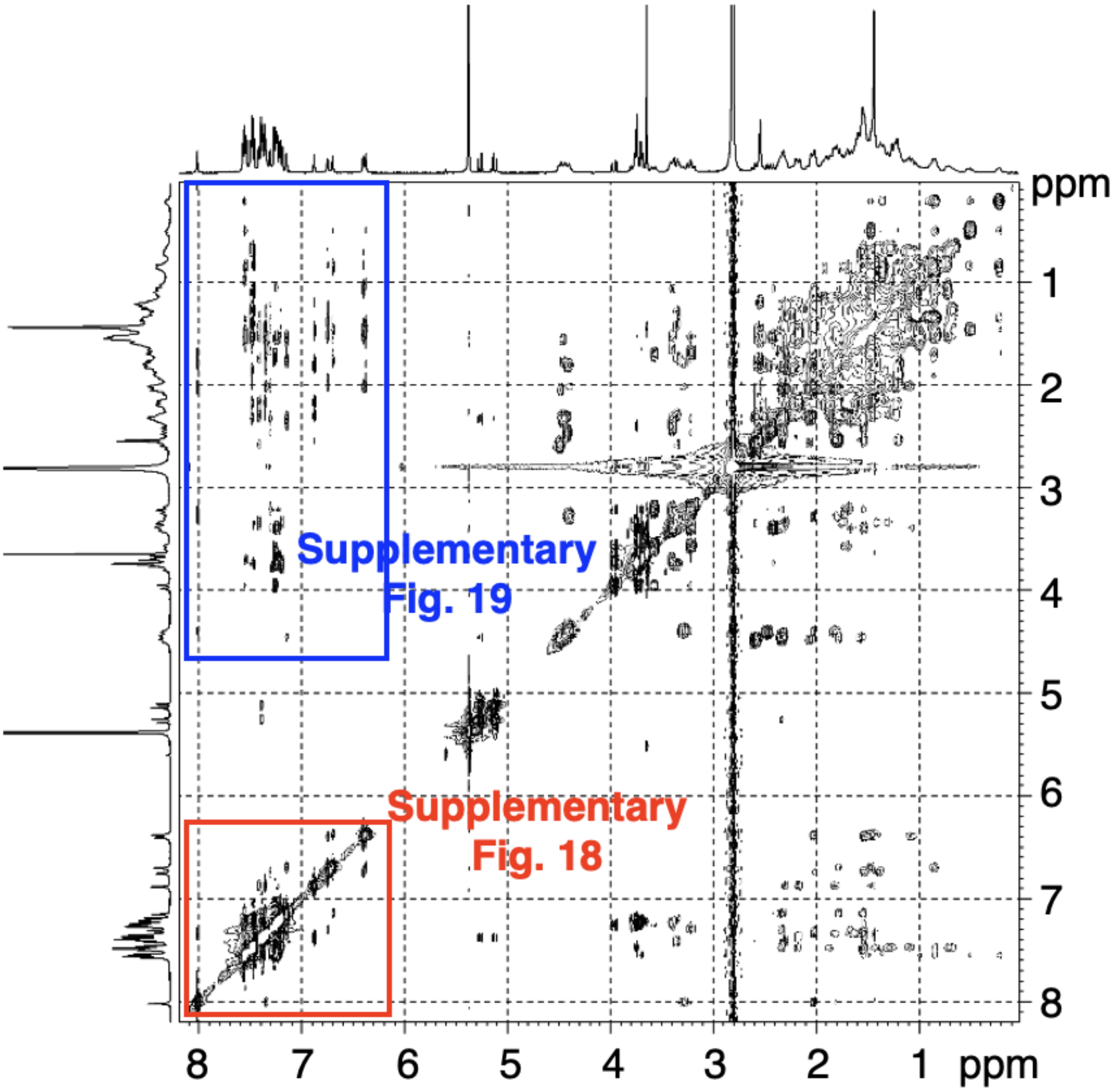

Supplementary Figure 17 | NOESY spectrum of dc2-Aib-OMe in $CD_2Cl_2/(CD_3)_2SO$ (95/5, v/v). ^{1}H-^{1}H NOESY (400 MHz, $CD_2Cl_2/(CD_3)_2SO$ (95/5, v/v), 3.0 mM) spectrum of **dc2-Aib-OMe** at 268 K: mixing time = 500 msec.

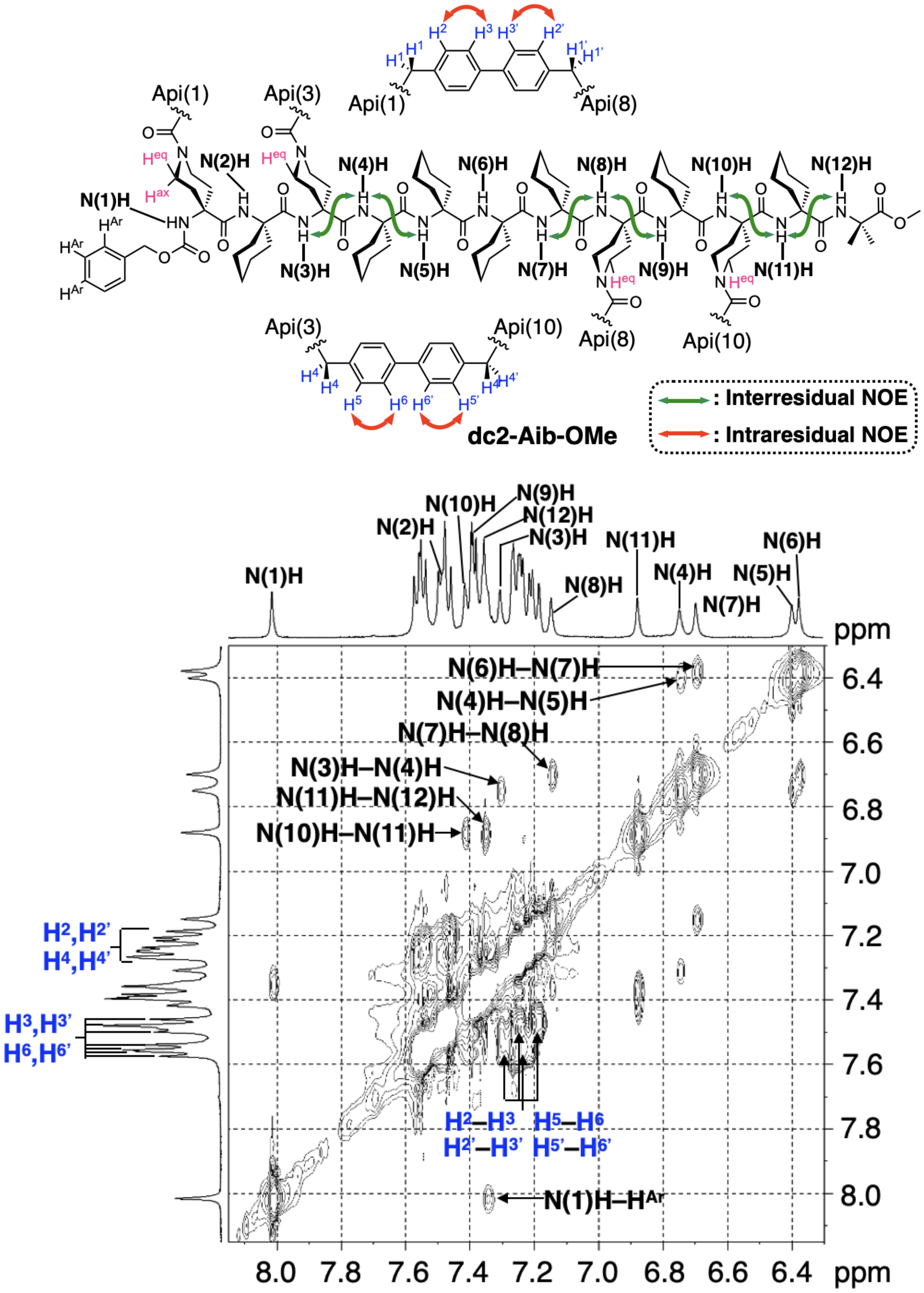

Supplementary Figure 18 | NOESY spectrum of dc2-Aib-OMe in $CD_2Cl_2/(CD_3)_2SO$ (95/5, v/v). Partial ^1H-^1H NOESY (400 MHz, $CD_2Cl_2/(CD_3)_2SO$ (95/5, v/v), 3.0 mM) spectrum of **dc2-Aib-OMe** at 268 K: mixing time = 500 msec.

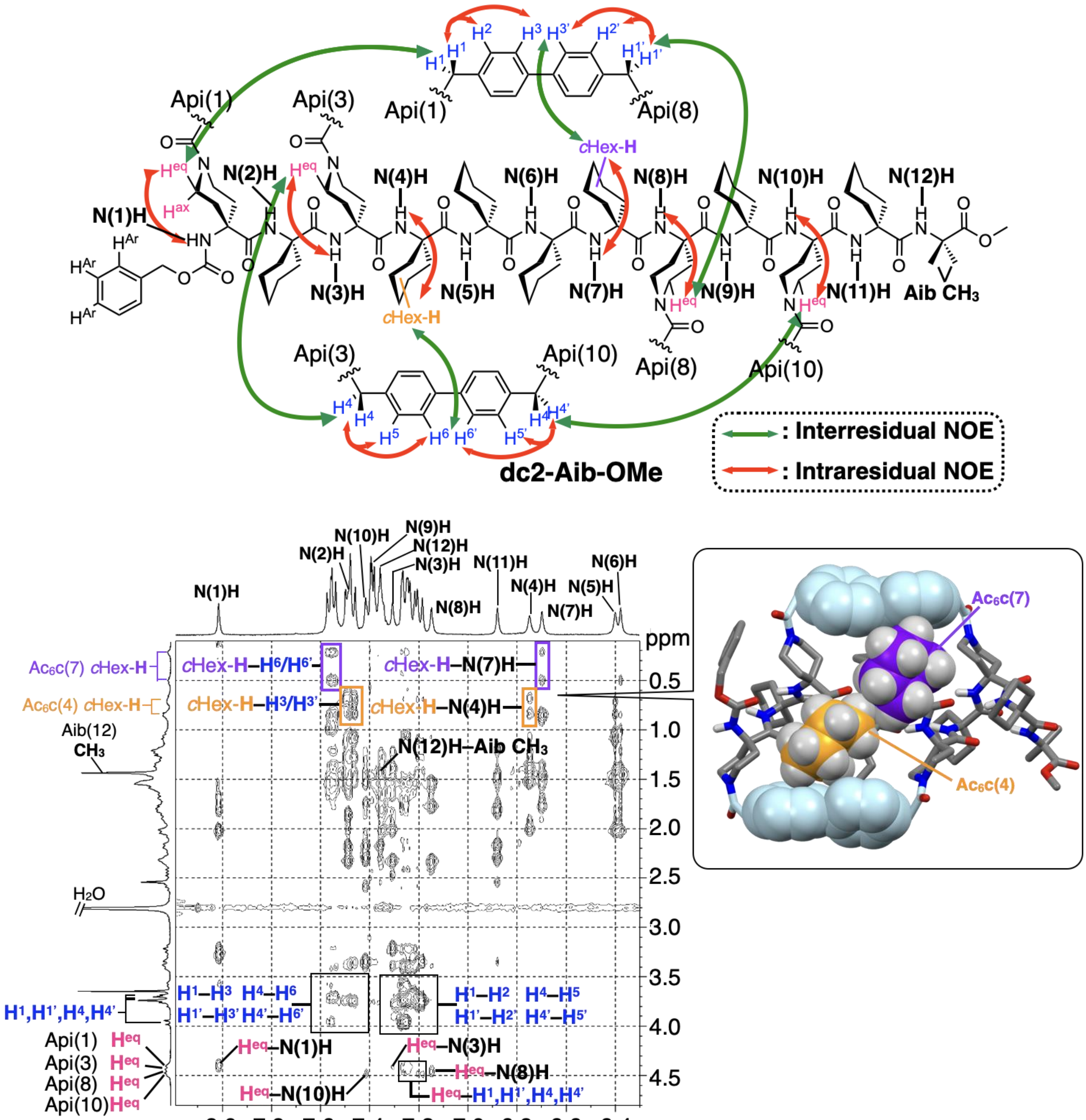

Supplementary Figure 19 | NOESY spectrum of dc2-Aib-OMe in $CD_2Cl_2/(CD_3)_2SO$ (95/5, v/v). Partial ^1H-^1H NOESY (400 MHz, $CD_2Cl_2/(CD_3)_2SO$ (95/5, v/v), 3.0 mM) spectrum of **dc2-Aib-OMe** at 268 K: mixing time = 500 msec. DFT-calculated structure of (*P*)-**dc2-Aib-OMe** is also shown. The cyclohexyl side chains of the $Ac_6c(4)$ and $Ac_6c(7)$ residues and the two biphenyl moieties are highlighted as a space-filling model.

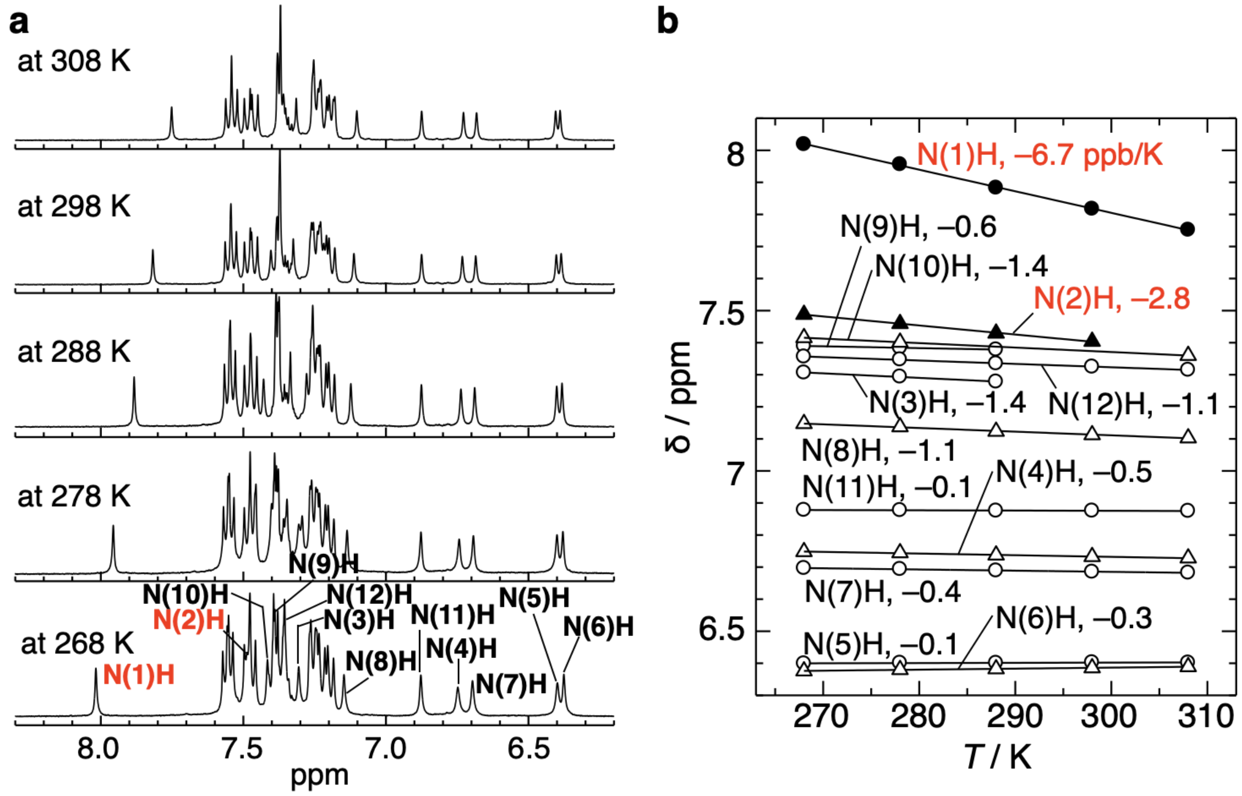

Supplementary Figure 20 | VT-^{1}H NMR spectra of dc2-Aib-OMe in $CD_2Cl_2/(CD_3)_2SO$ (95/5, v/v). a, Temperature-dependent ^{1}H NMR (400 MHz, $CD_2Cl_2/(CD_3)_2SO$ (95/5, v/v), 3.0 mM) spectral changes of **dc2-Aib-OMe**. **b**, Temperature dependence on the NH chemical shifts of **dc2-Aib-OMe** in $CD_2Cl_2/(CD_3)_2SO$ (95/5, v/v) in 400 MHz ^{1}H NMR spectra. The number in parentheses indicates the residue numbers of the amide NH protons from the N-terminus. For the NH signal assignment, see Supplementary Figures 17–19. Temperature coefficients (ppb/K) of each amide NH proton signal are also shown.

9. Determination of the Molar Ratios of (*P*)- and (*M*)-c1-Val-O^{t}Bu and (*P*)- and (*M*)-c1-Val-OH by ^{1}H NMR and CD Spectroscopies

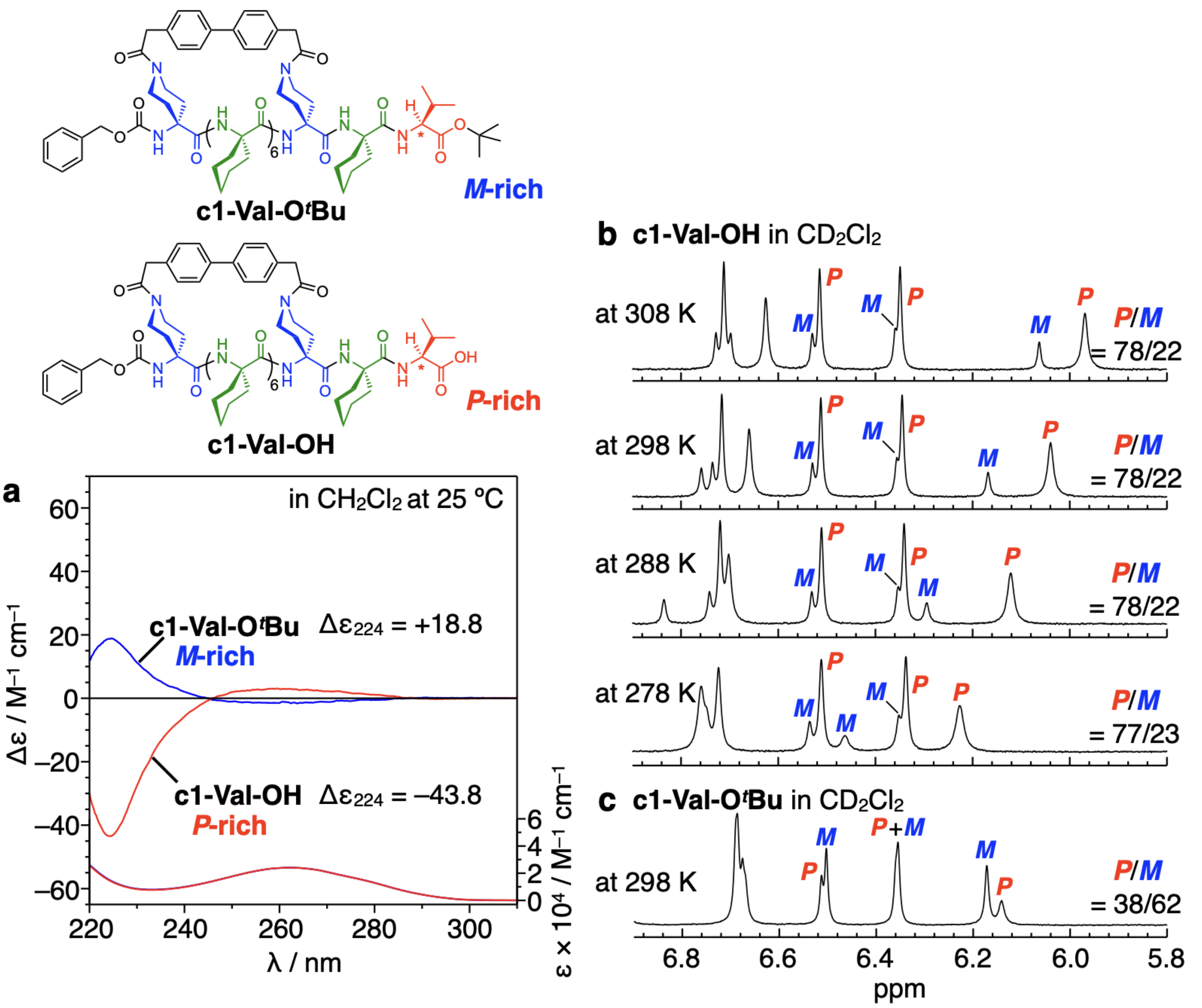

Supplementary Figure 21 | Determination of the molar ratios of (*P*)- and (*M*)-c1-Val-O^{t}Bu and (*P*)- and (*M*)- c1-Val-OH by ^{1}H NMR and CD spectroscopies. a, CD (top) and absorption (bottom) spectra of **c1-Val-O^{t}Bu** (blue line) and **c1-Val-OH** (red line) in CH_2Cl_2 at 25 ºC: [**c1-Val-O^{t}Bu**] = [**c1-Val-OH**] = 0.50 mM. **b**, Temperature-dependent ^{1}H NMR (400 MHz) spectral changes of **c1-Val-OH** in CD_2Cl_2 at the thermodynamic equilibrium state at each temperature: [**c1-Val-OH**] =1.5 mM. **c**, ^{1}H NMR (400 MHz) spectrum of **c1-Val-O^{t}Bu** in CD_2Cl_2 at 298 K: [**c1-Val-O^{t}Bu**] = 2.0 mM.

10. Kinetic and Thermodynamic Analyses of Acid/Base-Triggered Reversible Helicity Inversion and Solvent-Induced Helicity Inversion of c1-Val-OH

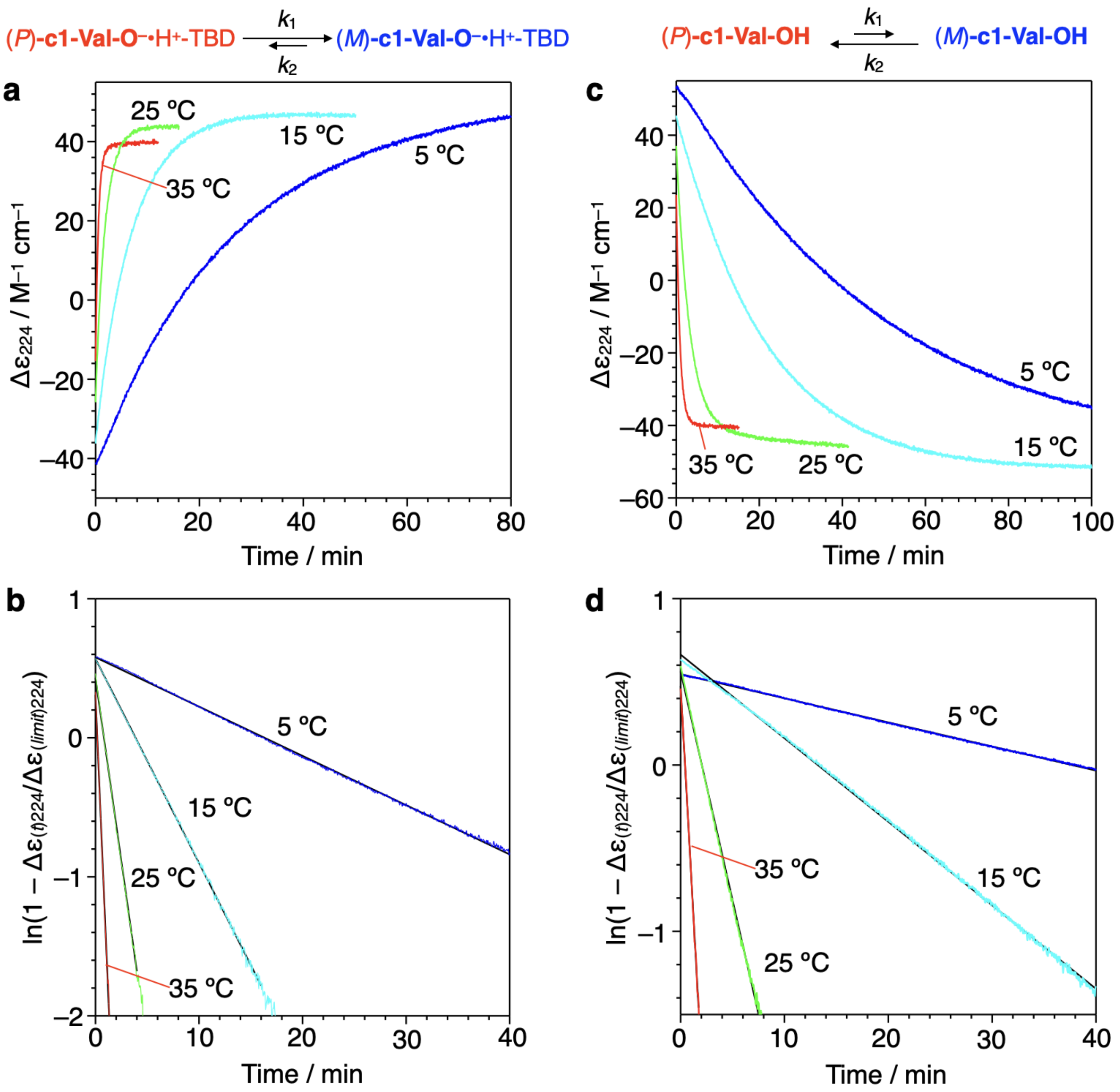

Supplementary Figure 22 | Kinetic analysis of acid/base-triggered reversible helicity inversion of c1-Val-OH. a, **c**, Time-dependent CD intensity changes at 224 nm ($\Delta\varepsilon_{224}$) for **c1-Val-OH** with TBD (1.2 equiv) (**a**) and for **c1-Val-OH**/TBD (1.2 equiv) with TFA (1.2 equiv) (**c**) in CH_2Cl_2 at 5, 15, 25 and 35 ºC: [**c1-Val-OH**] = 0.47–0.45 mM. The CD intensity changes were monitored immediately after the addition of TBD or TFA. **b**, **d**, Plots of ln(1 – $\Delta\varepsilon_{(t)224}/\Delta\varepsilon_{(limit)224}$) versus time for **c1-Val-OH** with TBD (1.2 equiv) (**b**) and **c1-Val-OH**/TBD (1.2 equiv) with TFA (1.2 equiv) (**d**) in CH_2Cl_2 at 5, 15, 25 and 35 ºC. $\Delta\varepsilon_{(limit)224}$ indicates the $\Delta\varepsilon_{224}$ value at an equilibrium.

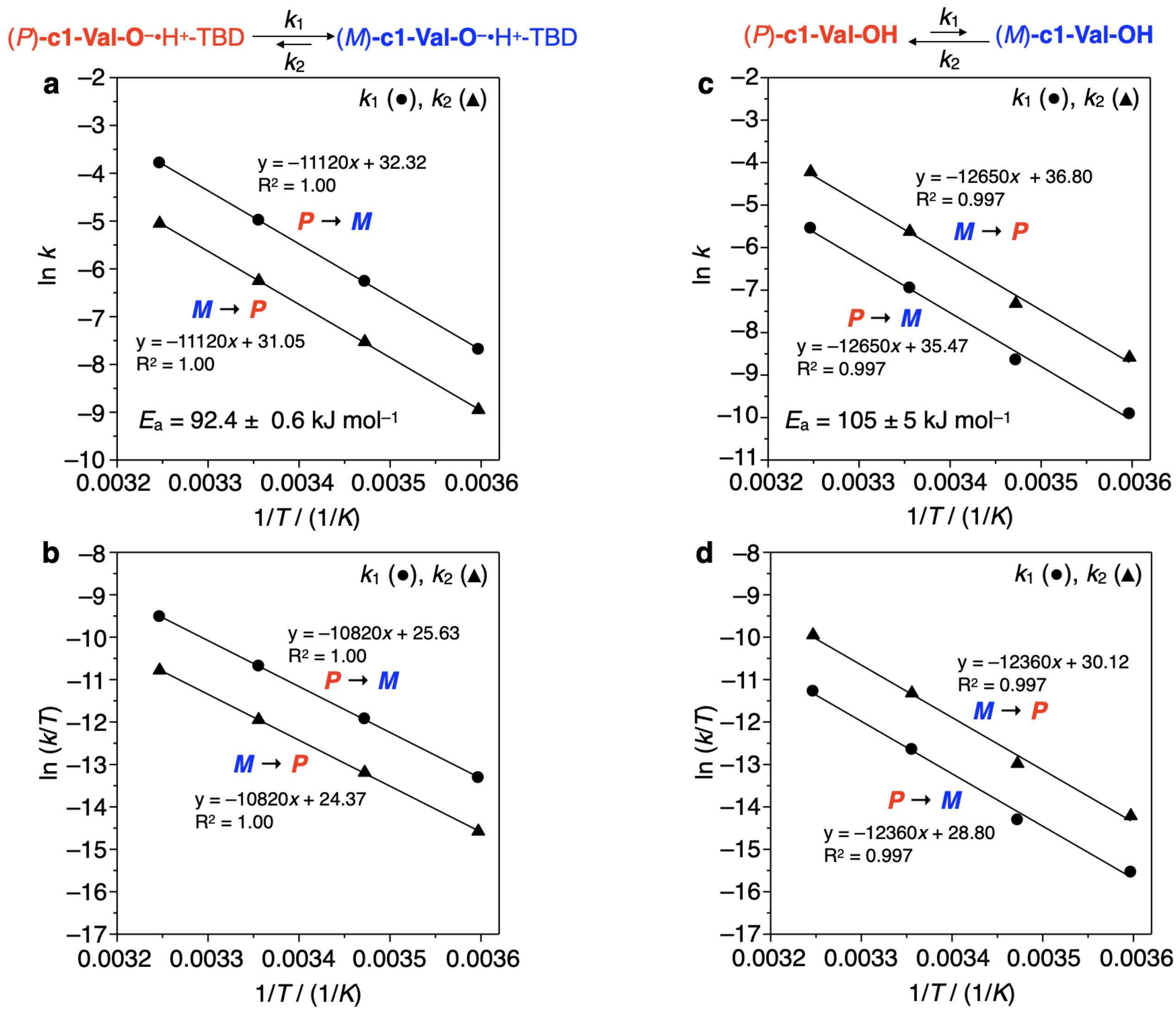

From ***P*** to ***M*** (k_1)

$\Delta H^{\ddagger} = 90.0 \pm 0.6$ kJ mol^{-1}

$\Delta S^{\ddagger} = 15.6 \pm 2.1$ J mol^{-1} K^{-1}

$\Delta G_{20}^{\ddagger} = 85.3 \pm 1.2$ kJ mol^{-1}

From ***M*** to ***P*** (k_2)

$\Delta H^{\ddagger} = 90.0 \pm 0.6$ kJ mol^{-1}

$\Delta S^{\ddagger} = 5.05 \pm 2.1$ J mol^{-1} K^{-1}

$\Delta G^{\ddagger}_{20} = 88.5 \pm 1.2$ kJ mol^{-1}

From ***P*** to ***M*** (k_1)

$\Delta H^{\ddagger} = 103 \pm 5$ kJ mol^{-1}

$\Delta S^{\ddagger} = 41.9 \pm 18.0$ J mol^{-1} K^{-1}

$\Delta G_{20}^{\ddagger} = 90.5 \pm 10.6$ kJ mol^{-1}

From ***M*** to ***P*** (k_2)

$\Delta H^{\ddagger} = 103 \pm 5$ kJ mol^{-1}

$\Delta S^{\ddagger} = 52.9 \pm 18.0$ J mol^{-1} K^{-1}

$\Delta G_{20}^{\ddagger} = 87.2 \pm 10.6$ kJ mol^{-1}

Supplementary Figure 23 | Thermodynamic analysis of acid/base-triggered reversible helicity inversion of c1-Val-OH. Arrhenius (**a**, **c**) and Eyring (**b**, **d**) plots for the interconversion between (*P*)- and (*M*)-**c1-Val-O**$^{-}$·H^{+}-TBD (**a**, **b**) and between (*P*)- and (*M*)-**c1-Val-OH** (**c**, **d**) (for data and condition, see Table 1). The obtained thermodynamic activation parameters are also shown.

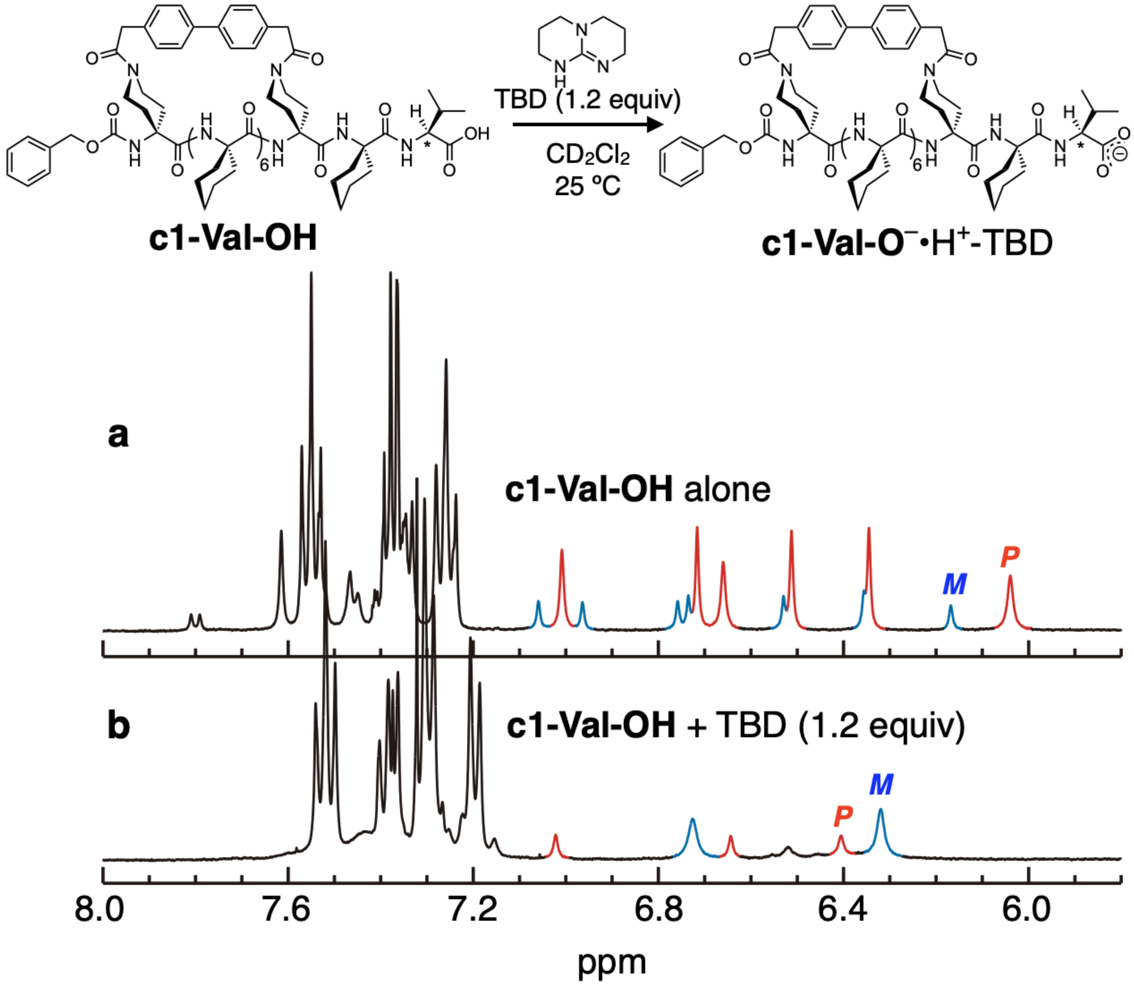

Supplementary Figure 24 | TBD-triggered ^{1}H NMR spectral changes of c1-Val-OH. ^{1}H NMR (400 MHz) spectra of **c1-Val-OH** in the absence (**a**) and presence (**b**) of TBD (1.2 equiv) in CD_2Cl_2 at the thermodynamic equilibrium state at 298 K: [**c1-Val-OH**] = 1.5 mM. The *P*/*M* assignments were made based on the CD measurement results shown in Fig. 2a(i),(ii).

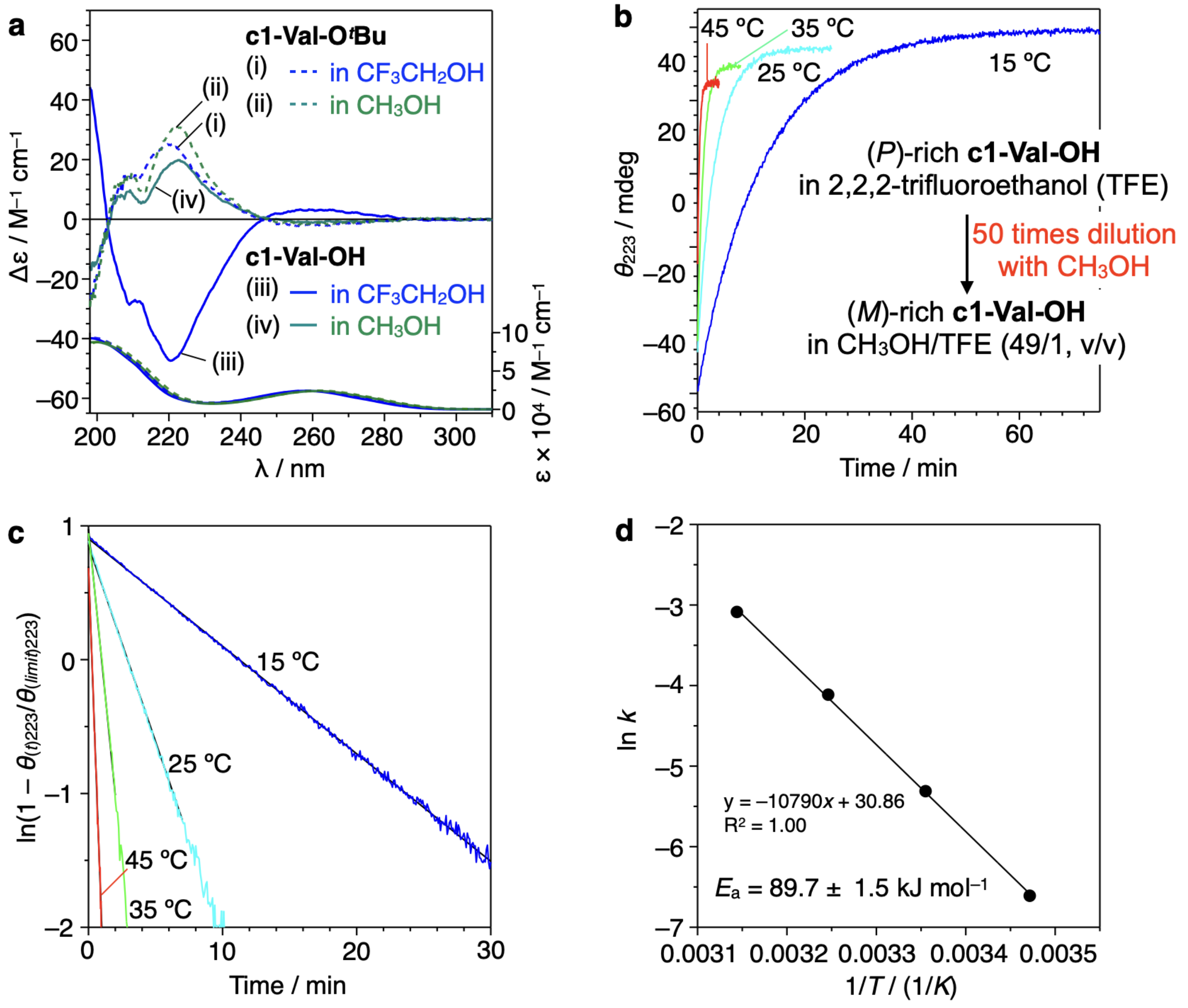

Supplementary Figure 25 | Solvent-induced helicity inversion of c1-Val-OH. **a**, CD (top) and absorption (bottom) spectra (1.8×10^{-5} M, 25 ºC) of **c1-Val-O^{t}Bu** in TFE (i) and MeOH (ii) and **c1-Val-OH** in TFE (iii) and MeOH (iv) at the thermodynamic equilibrium state. **b**, Time-dependent CD intensity changes at 223 nm (θ_{223}) for **c1-Val-OH** in MeOH/TFE (95/5, v/v) at 15, 25, 35 and 45 ºC. The CD intensity changes were monitored immediately after 50 times dilution of a 50 µL aliquot of a stock solution of **c1-Val-OH** in TFE ([**c1-Val-OH**] = 0.92 mM) with 2.45 mL of MeOH in 1.0 cm quartz cell at 15, 25, 35 and 45 ºC. **c**, Plots of ln(1 – $\theta_{(t)223}/\theta_{(limit)223}$) versus time for **c1-Val-OH** upon 50 times dilution of a TFE solution of **c1-Val-OH** with MeOH at 15, 25, 35 and 45 ºC. $\theta_{(limit)223}$ indicates the θ_{223} value at an equilibrium. **d**, Arrhenius plot for the interconversion between (*P*)- and (*M*)-**c1-Val-OH** (for data and condition, see Supplementary Table 12). The obtained activation energy (E_a) is also shown.

Supplementary Table 12. Rate constants (k (= $k_1 + k_2$), sec^{-1}) and half-life time ($t_{1/2}$, min) for the interconversion between (*P*)- and (*M*)-**c1-Val-OH** in MeOH/TFE (49/1, v/v)[a]

temp (°C)	k (sec^{-1})	$t_{1/2}$ (min)[d]
45	4.54×10^{-2} [b]	0.3
35	1.63×10^{-2} [b]	0.7
25	4.91×10^{-3} [b]	2.4
15	1.34×10^{-3} [c]	8.6

[a]Conditions: in MeOH/TFE (49/1, v/v), [**c1-Val-OH**] = 1.8×10^{-5} M. [b]Estimated from the time-dependent CD intensity changes (Supplementary Fig. 21b). [c]Half–life time ($t_{1/2}$) was obtained from the following equation: $t_{1/2}$ (min) = ln2/($k \times 60$) = 0.693/($k \times 60$).

11. Determination of the Association Constants of c1-Val-OH with Various Organic Bases

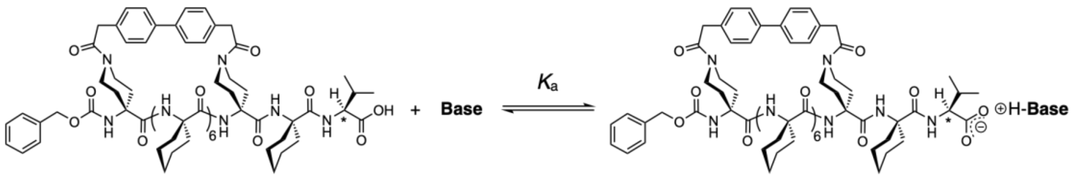

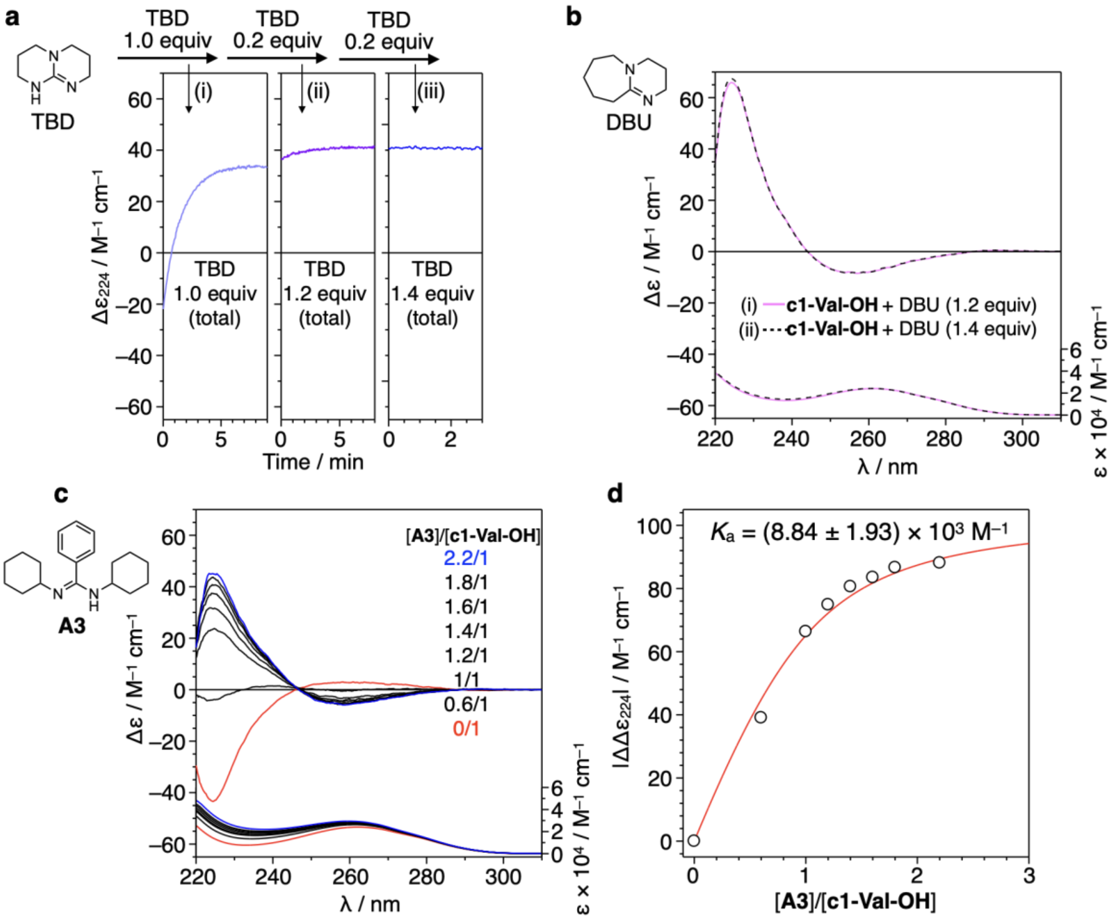

Supplementary Figure 26 | Determination of the Association Constants of c1-Val-OH with Various Organic Bases. a, Time-dependent CD intensity changes at 224 nm ($\Delta\varepsilon_{224}$) for **c1-Val-OH** + TBD (1.0 equiv) (i) and (i) + 0.2 equiv of TBD (total = 1.2 equiv) (ii) and (ii) + 0.2 equiv of TBD (total = 1.4 equiv) (iii) in CH_2Cl_2 at 25 ºC: [**c1-Val-OH**]$_0$ = 0.50 mM. **b**, CD (top) and absorption (bottom) spectra of **c1-Val-OH** in the presence of 1.2 (i) and 1.4 (ii) equiv of DBU in CH_2Cl_2 at the thermodynamic equilibrium state at 25 ºC: [**c1-Val-OH**]$_0$ = 0.50 mM. **c**, CD (top) and absorption (bottom) spectral changes of **c1-Val-OH** upon the addition of **A3** in CH_2Cl_2 at 25 ºC: [**c1-Val-OH**]$_0$ = 0.50 mM. The spectra were measured after reaching the thermodynamic equilibrium state. **d**, Plots of CD intensity changes ($\Delta\Delta\varepsilon_{224}$) of **c1-Val-OH** at 224 nm versus the molar ratio of [**A3**]/[**c1-Val-OH**]. The curve in the plots and the association constant (K_a) of **c1-Val-OH** with **A3** were obtained by the least-squares curve-fitting method based on a 1:1 stoichiometry using OpenDataFit (Bind Fit) software (http://app.supramolecular.org/bindfit/).

The $\Delta\varepsilon_{224}$ value of **c1-Val-OH** in the presence of 1.2 equiv of TBD reached a plateau value at the thermodynamic equilibrium and did not change by the further addition of TBD (total = 1.4 equiv), indicating that 1.2 equiv of TBD is enough to deprotonate most of the C-terminal CO_2H group of **c1-Val-OH** (>98%) (Supplementary Fig. 26a). Based on simulated 1:1 binding curves with various K_a values (Supplementary Fig. 27), the K_a value of **c1-Val-OH** with TBD was estimated to be $>10^6$ M^{-1}. Similarly, the K_a value for DBU was estimated to be $>10^6$ M^{-1} (Supplementary Fig. 26b).

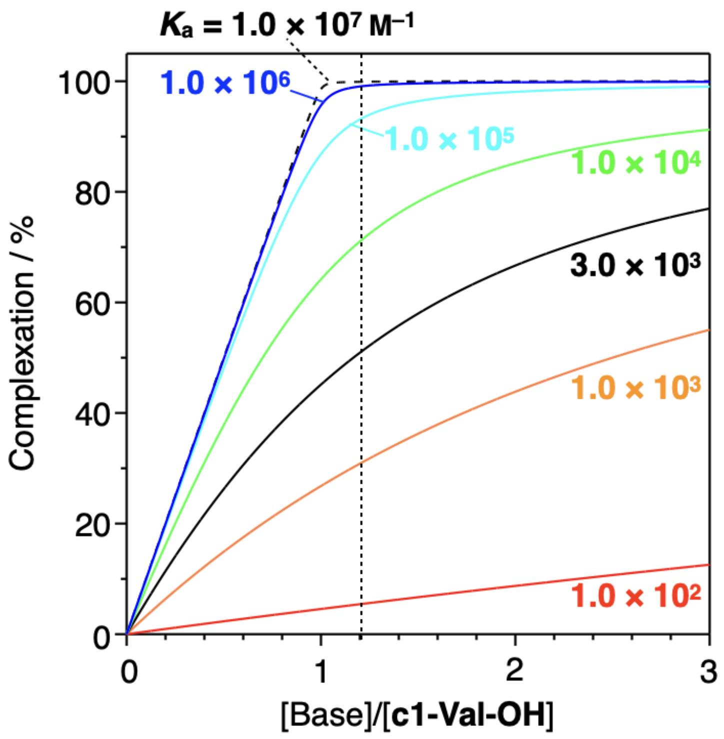

$$\text{Complexation (\%)} = \frac{100(K_a[\textbf{c1-Val-OH}] + xK_a + 1 - \{(-K_a[\textbf{c1-Val-OH}] + xK_a - 1)^2 - 4xK_a^2[\textbf{c1-Val-OH}])^{1/2}\}}{2K_a[\textbf{c1-Val-OH}]}$$

$$x = [\text{Base}]/[\textbf{c1-Val-OH}]$$

Supplementary Figure 27 | Simulated 1:1 binding curves for the complexation of c1-Val-OH with base. Simulated 1:1 binding curves using various K_a values with a fixed concentration of **c1-Val-OH** (5.0×10^{-4} M).

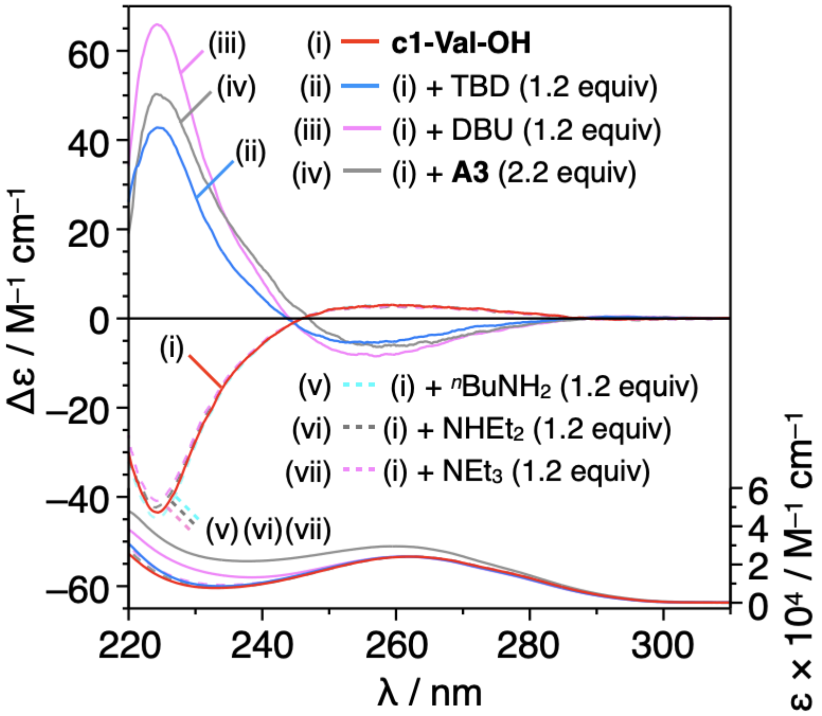

Supplementary Figure 28 | Base-triggered CD spectral changes of c1-Val-OH. CD (top) and absorption (bottom) spectra of **c1-Val-OH** (i) with TBD (1.2 equiv) (ii), DBU (1.2 equiv) (iii), **A3** (2.2 equiv) (iv), ^{n}BuNH$_2$ (1.2 equiv) (v), NHEt$_2$ (1.2 equiv) (vi) and NEt$_3$ (1.2 equiv) (vii) in CH_2Cl_2 at the thermodynamic equilibrium state at 25 °C: [**c1-Val-OH**]$_0$ = 0.50 mM.

The $\Delta\varepsilon_{224}$ value of **c1-Val-OH** did not change upon the addition of 1.2 equiv of primary, secondary and tertiary aliphatic amines (^{n}BuNH$_2$, NHEt$_2$ and NEt$_3$, respectively), most likely due to their low K_a values for **c1-Val-OH** (Supplementary Fig. 28(v)–(vii)). Based on simulated 1:1 binding curves as shown in Supplementary Fig. 27, these K_a values were estimated to be <10^2.

12. Kinetic and Thermodynamic Analyses of the Transformation of the Kinetically-Trapped (*M*)-Rich dc2-Val-OH into the Thermodynamically-Stable (*P*)-Rich dc2-Val-OH

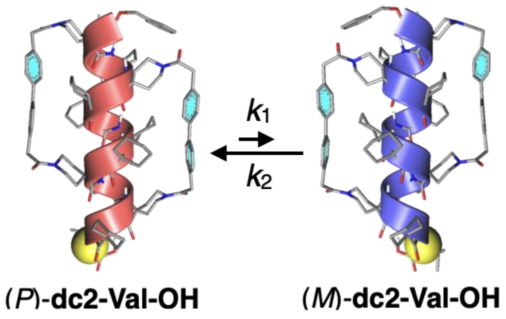

Supplementary Figure 29 | Interconversion between (*P*)- and (*M*)-dc2-Val-OH.

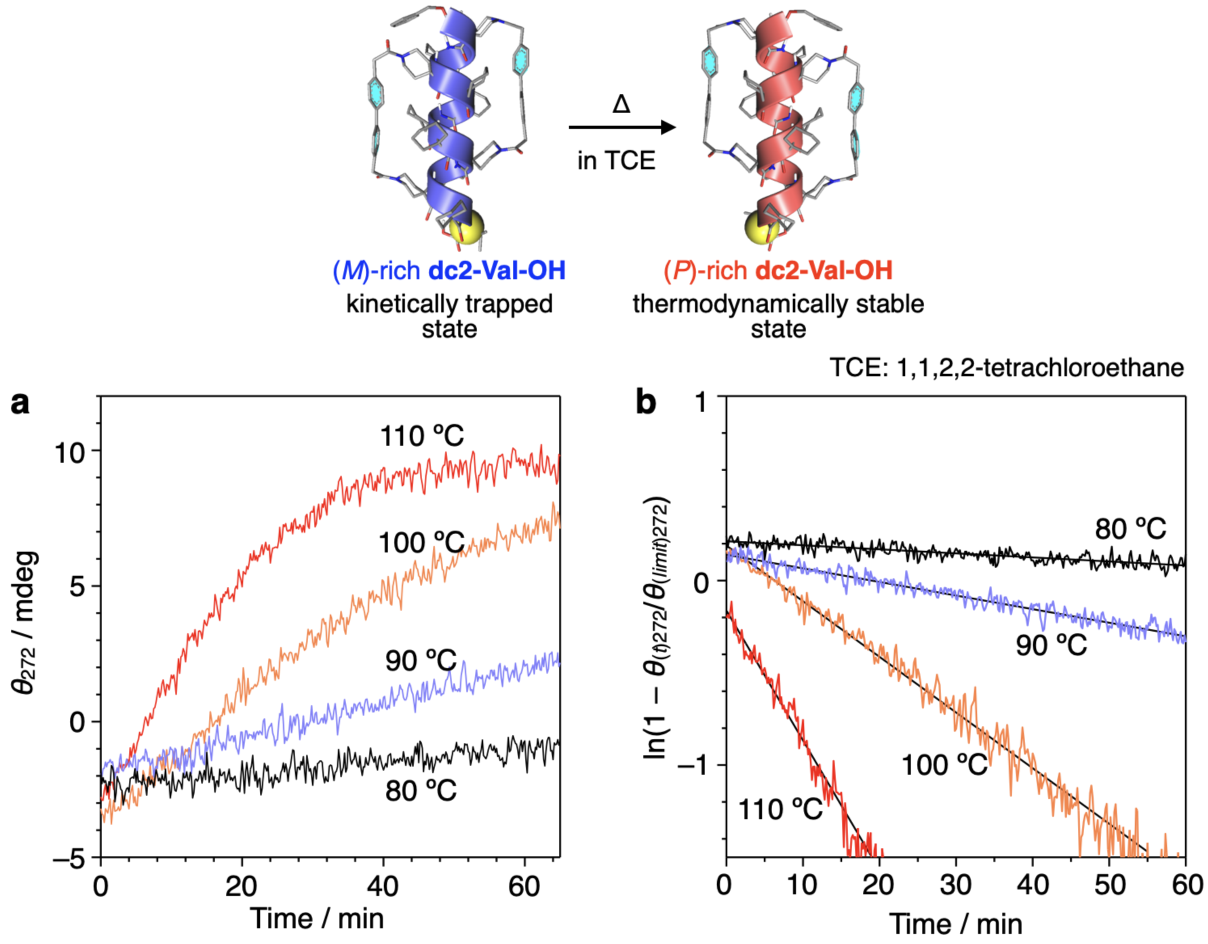

Supplementary Figure 30 | Kinetic analysis of helicity inversion of dc2-Val-OH. a, Time-dependent CD intensity changes at 272 nm (θ_{272}) for the as-obtained **dc2-Val-OH** in TCE at 80, 90, 100 and 110 ºC: [**dc2-Val-OH**] = 0.51 mM. CD intensity changes were monitored after reaching the desired temperature. **b**, Plots of ln(1– $\theta_{(t)272}/\theta_{(limit)272}$) versus time for the as-obtained **dc2-Val-OH** in TCE at 80, 90, 100 and 110 ºC. $\theta_{(limit)272}$ indicates the θ_{272} value at an equilibrium.

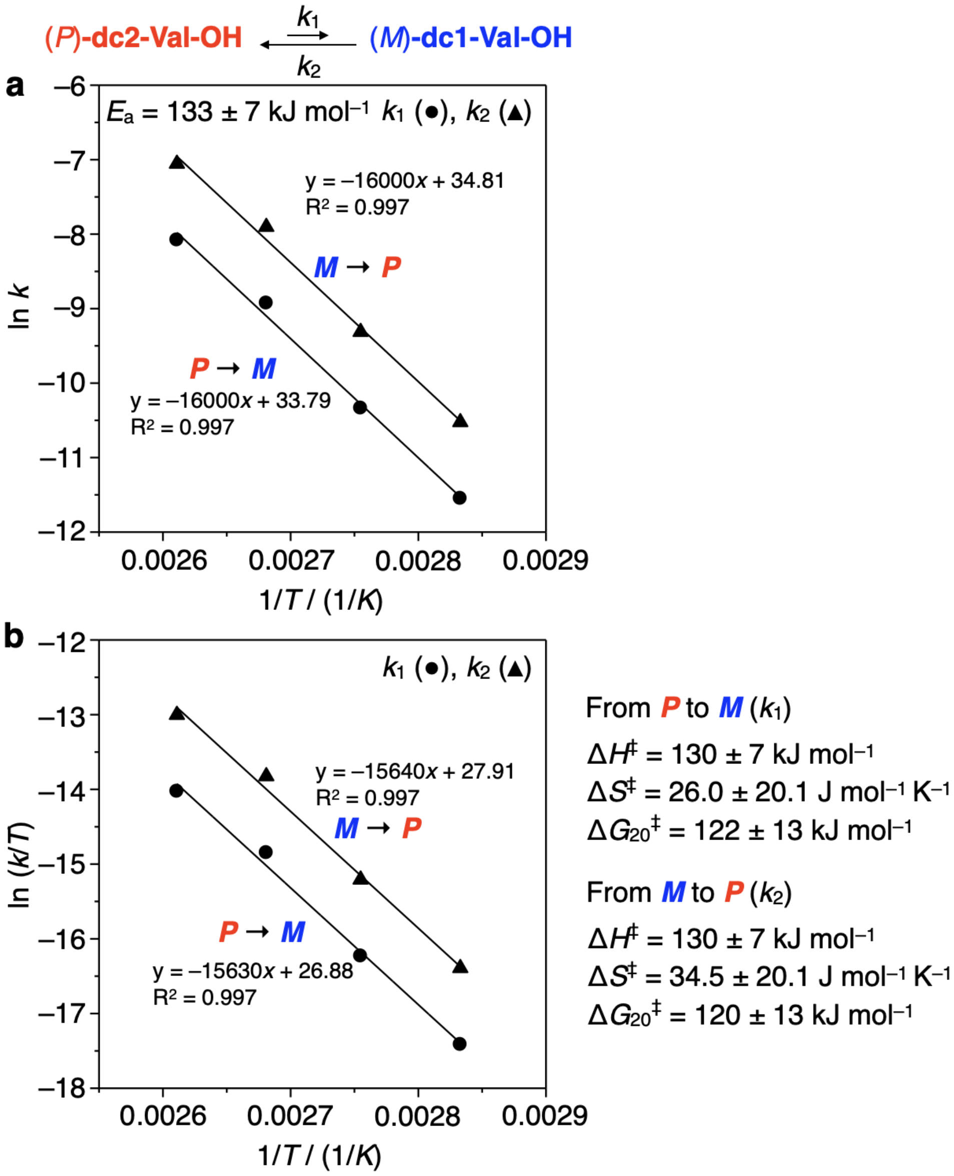

Supplementary Figure 31 | Thermodynamic analysis of helicity inversion of dc2-Val-OH. Arrhenius (**a**) and Eyring (**b**) plots for the interconversion between (*P*)- and (*M*)-**dc2-Val-OH** (for data and condition, see Table 3). The obtained thermodynamic activation parameters are also shown.

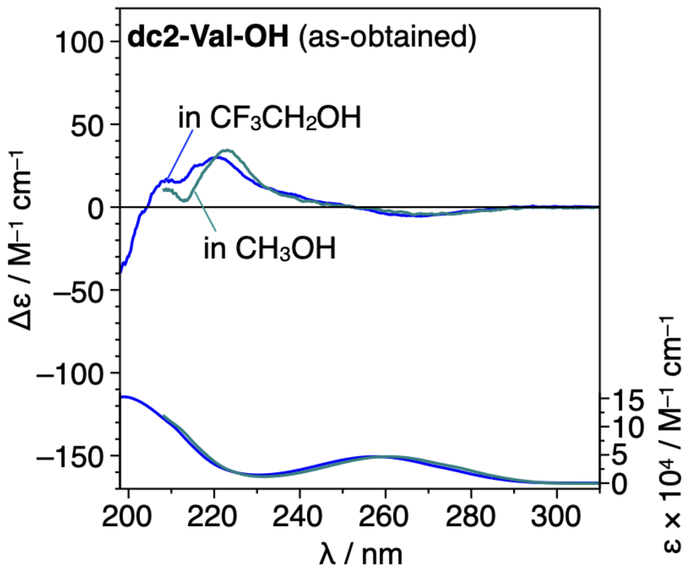

Supplementary Figure 32 | Solvent-dependent CD and absorption spectral changes of dc2-Val-OH. CD (top) and absorption (bottom) spectra of the as-obtained **dc2-Val-OH** in TFE (blue line) and MeOH (green line) at 25 ºC: [**dc2-Val-OH**] = 0.10 mM in TFE and 9.9 μM in MeOH.

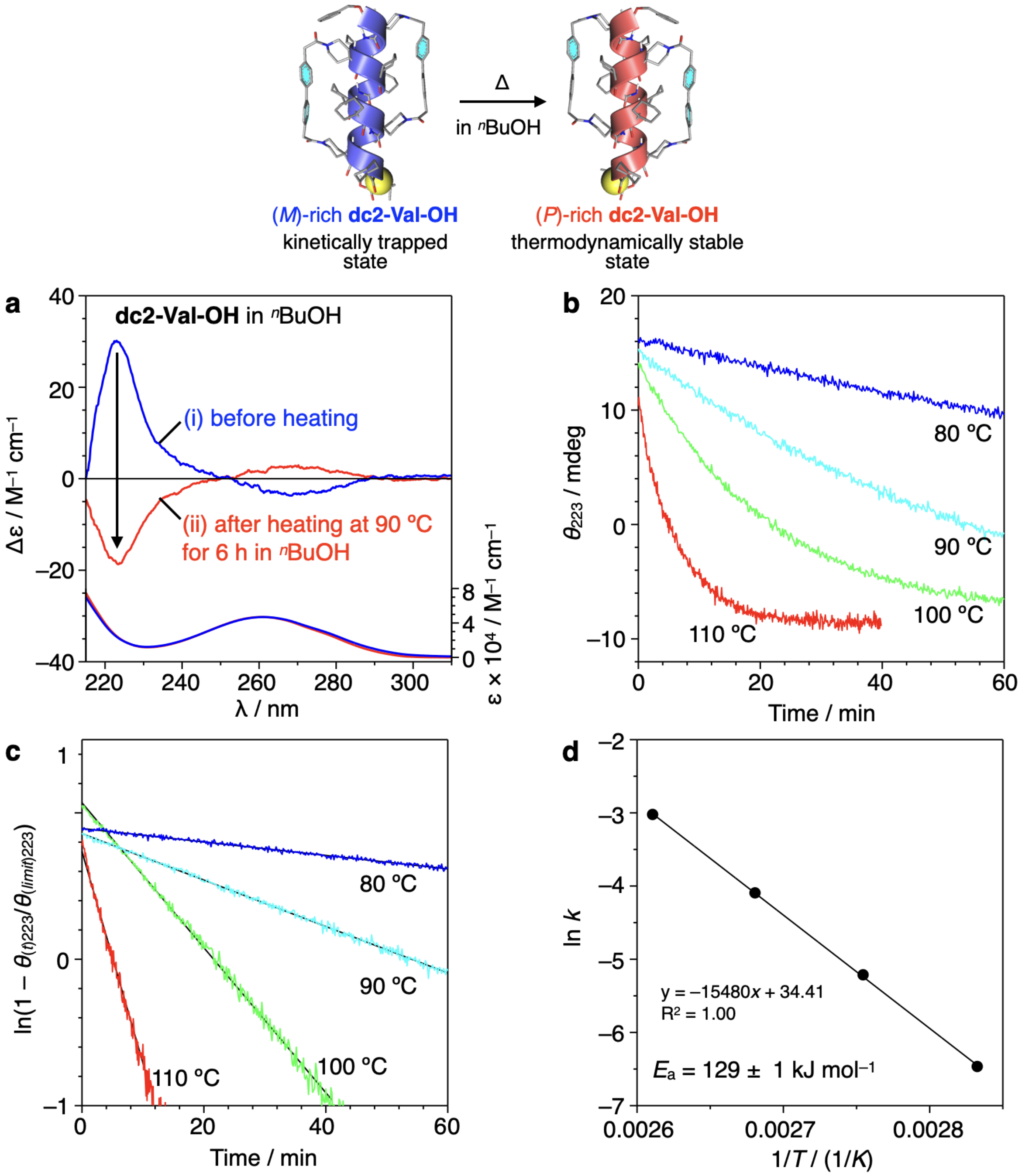

Supplementary Figure 33 | Solvent-induced helicity inversion of dc2-Val-OH. **a**, CD (top) and absorption (bottom) spectra (1.6×10^{-5} M, 25 ºC) of the as-obtained **dc2-Val-OH** (i) and the annealed **dc2-Val-OH** in ^{n}BuOH. **b**, Time-dependent CD intensity changes at 223 nm (θ_{223}) for the as-obtained **dc2-Val-OH** in ^{n}BuOH at 80, 90, 100 and 110 ºC: [**dc2-Val-OH**] = 1.6×10^{-5} M. CD intensity changes were monitored after reaching the desired temperature. **c**, Plots of ln(1– $\theta_{(t)223}/\theta_{(limit)223}$) versus time for the as-obtained **dc2-Val-OH** in ^{n}BuOH at 80, 90, 100 and 110 ºC. $\theta_{(limit)223}$ indicates the θ_{223} value at an equilibrium. **d**, Arrhenius plot for the interconversion between (*P*)- and (*M*)-**dc2-Val-OH** (for data and condition, see Supplementary Table 13). The obtained activation energy (E_a) is also shown.

Supplementary Table 13. Rate constants (k (= $k_1 + k_2$), sec^{-1}) and half-life time ($t_{1/2}$, min) for the interconversion between (*P*)- and (*M*)-**dc2-Val-OH** in ^{n}BuOH[a]

temp (°C)	k (sec^{-1})	$t_{1/2}$ (min)[d]
110	2.41×10^{-3} [b]	4.8
100	8.25×10^{-4} [b]	14
90	2.70×10^{-4} [b]	43
80	7.7×10^{-5} [c]	150

[a]Conditions: in ^{n}BuOH, [**dc2-Val-OH**] = 1.6×10^{-5} M. [b]Estimated from the time-dependent CD intensity changes (Supplementary Fig. 28b). [c]Half–life time ($t_{1/2}$) was obtained from the following equation: $t_{1/2}$ (min) = $\ln 2/(k \times 60) = 0.693/(k \times 60)$.

13. Kinetic and Thermodynamic Analyses of the Racemization of (*M*)-dc2-Aib-OMe

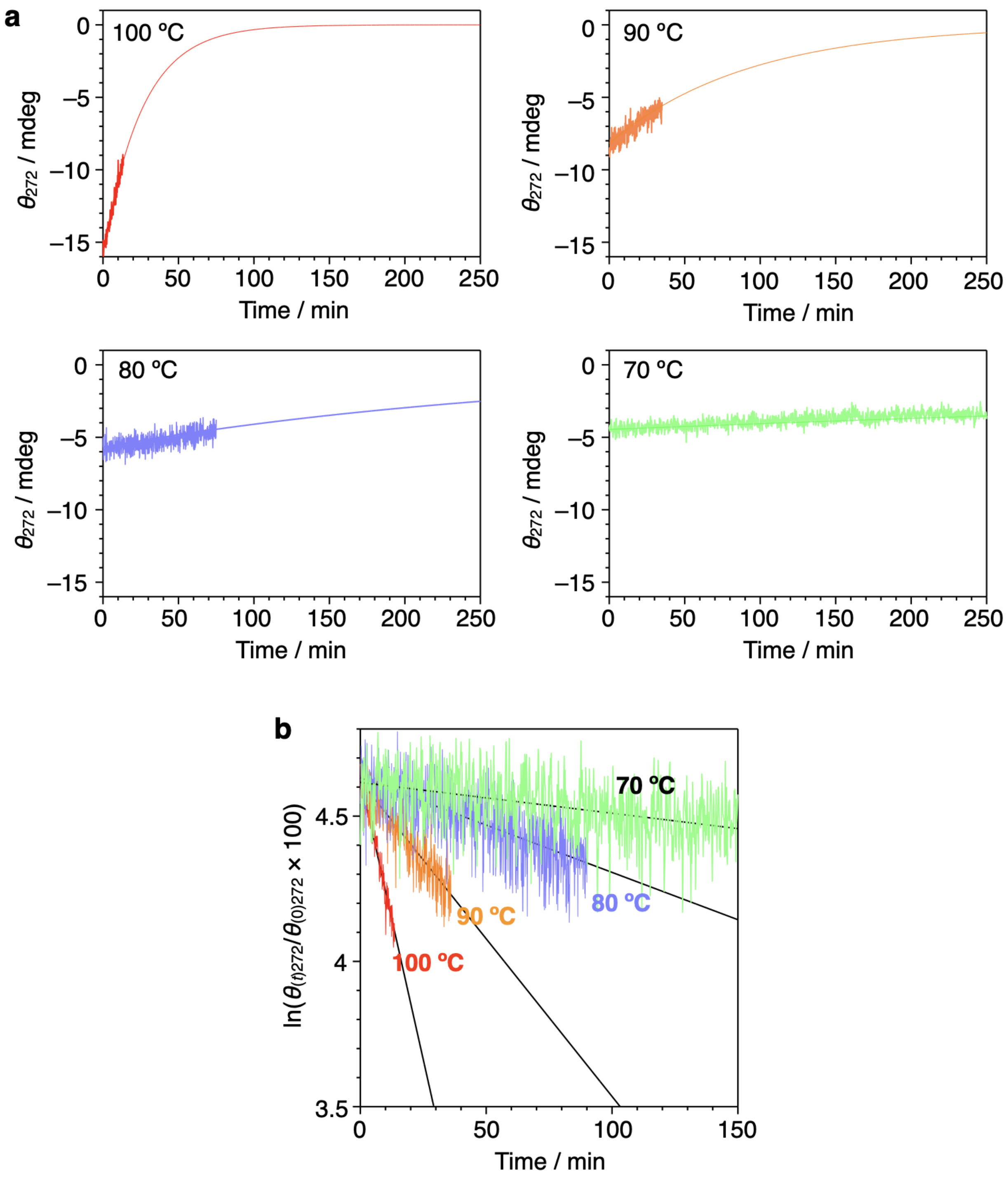

Supplementary Figure 34 | Racemization kinetics of (*M*)-dc2-Aib-OMe in TCE. a, Time-dependent CD intensity changes at 272 nm (θ_{272}) for (*M*)-**dc2-Aib-OMe** in TCE at 70, 80, 90 and 100 ºC: [**dc2-Aib-OMe**] = 0.46 mM. The CD intensity changes were monitored after reaching the desired temperature. Simulated kinetic curves ($\theta_{(t)272} = \theta_{(0)272} \cdot \exp(-2k_{\mathrm{rac}}t \cdot 60)$) for the racemization of (*M*)-**dc2-Aib-OMe** are also shown. **b**, Plots of $\ln(\theta_{(t)272}/\theta_{(0)272})$ versus time for (*M*)-**dc2-Aib-OMe** in TCE at 70, 80, 90 and 100 ºC.

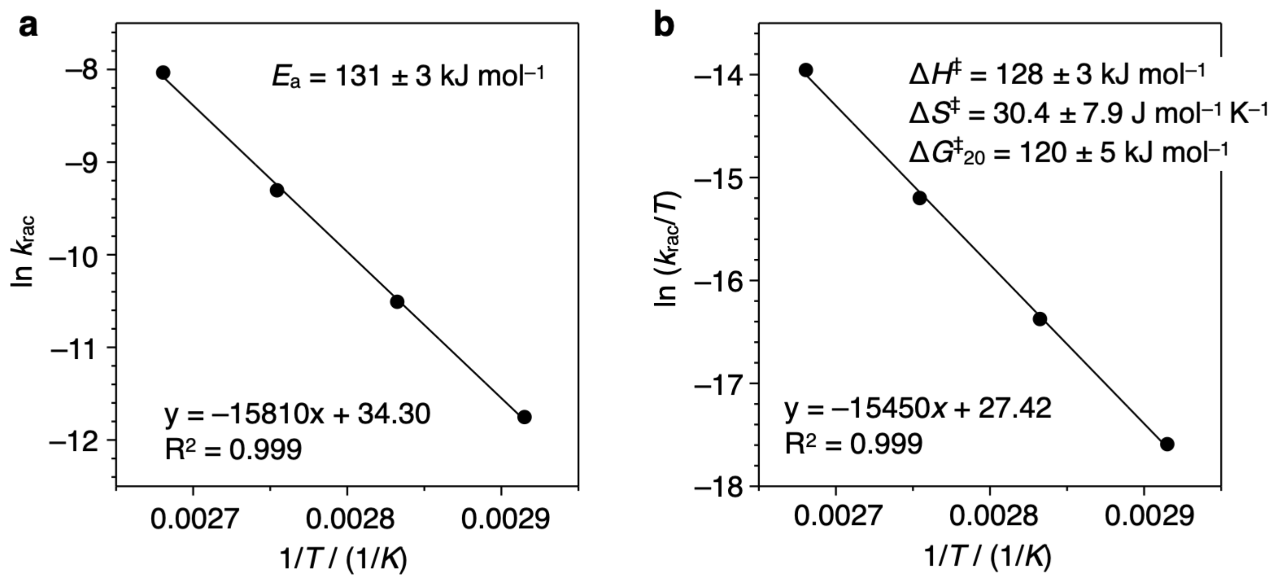

Supplementary Figure 35 | Thermodynamic analysis of the racemization of (*M*)-dc2-Aib-OMe. Arrhenius (**a**) and Eyring (**b**) plots for the racemization of (*M*)-**dc2-Aib-OMe** (for data and condition, see Table 4). The obtained thermodynamic activation parameters are also shown.

14. ^{1}H and ^{13}C NMR Spectra

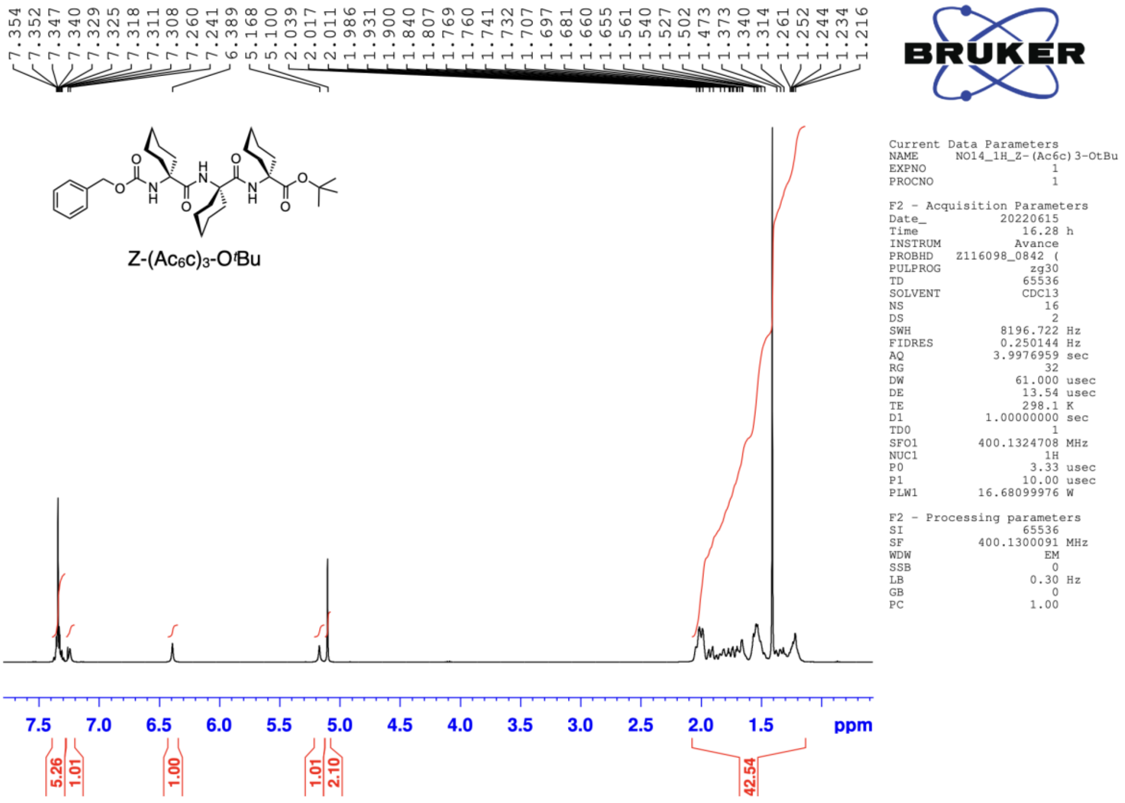

^{1}H NMR (400 MHz, $CDCl_3$, 25 ºC) spectrum of Z-$(Ac_6c)_3$-O^{t}Bu.

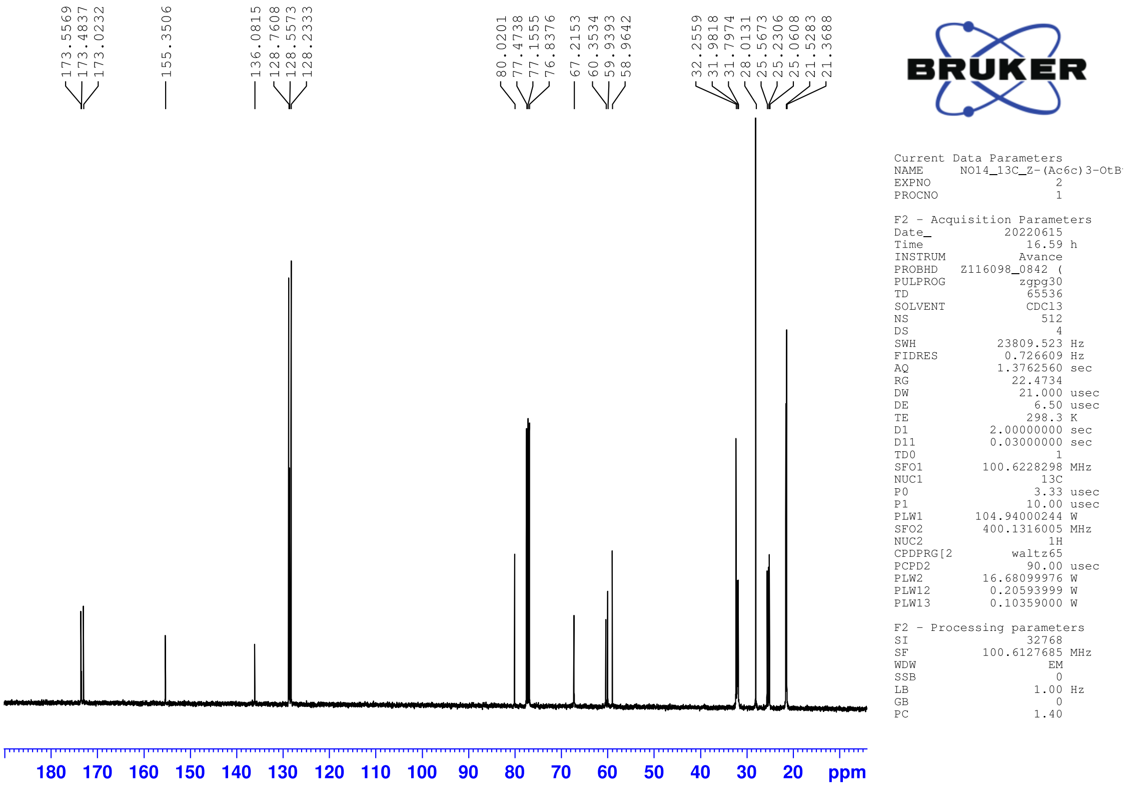

^{13}C NMR (100 MHz, $CDCl_3$, 25 ºC) spectrum of Z-$(Ac_6c)_3$-O^{t}Bu.

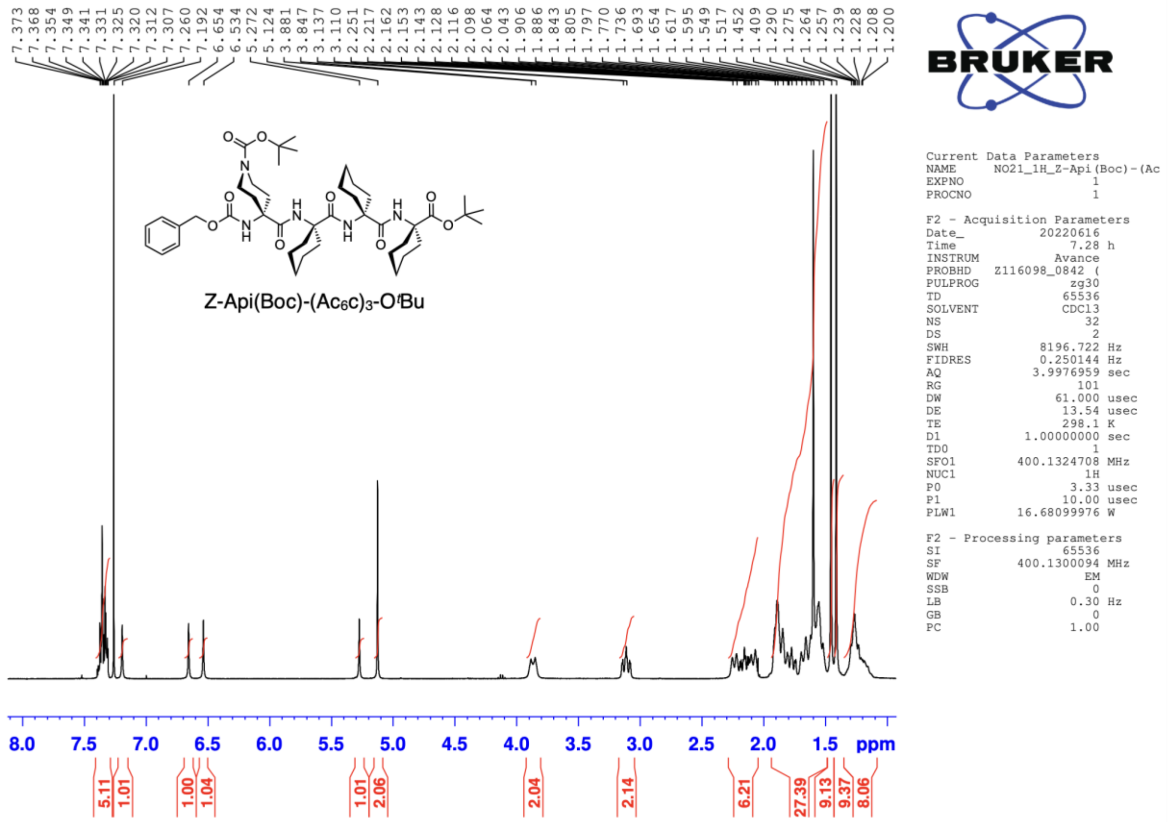

^{1}H NMR (400 MHz, $CDCl_3$, 25 ºC) spectrum of Z-Api(Boc)-(Ac_6c)$_3$-O^{t}Bu.

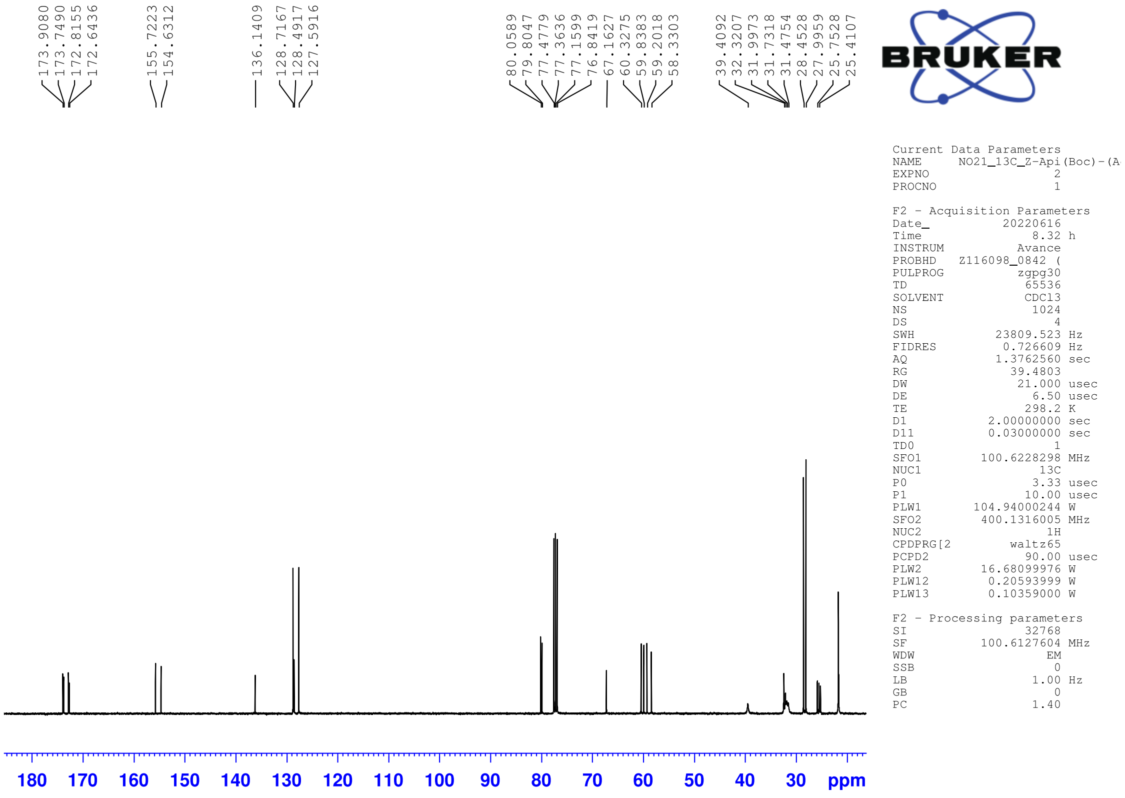

^{13}C NMR (100 MHz, $CDCl_3$, 25 ºC) spectrum of Z-Api(Boc)-(Ac_6c)$_3$-O^{t}Bu.

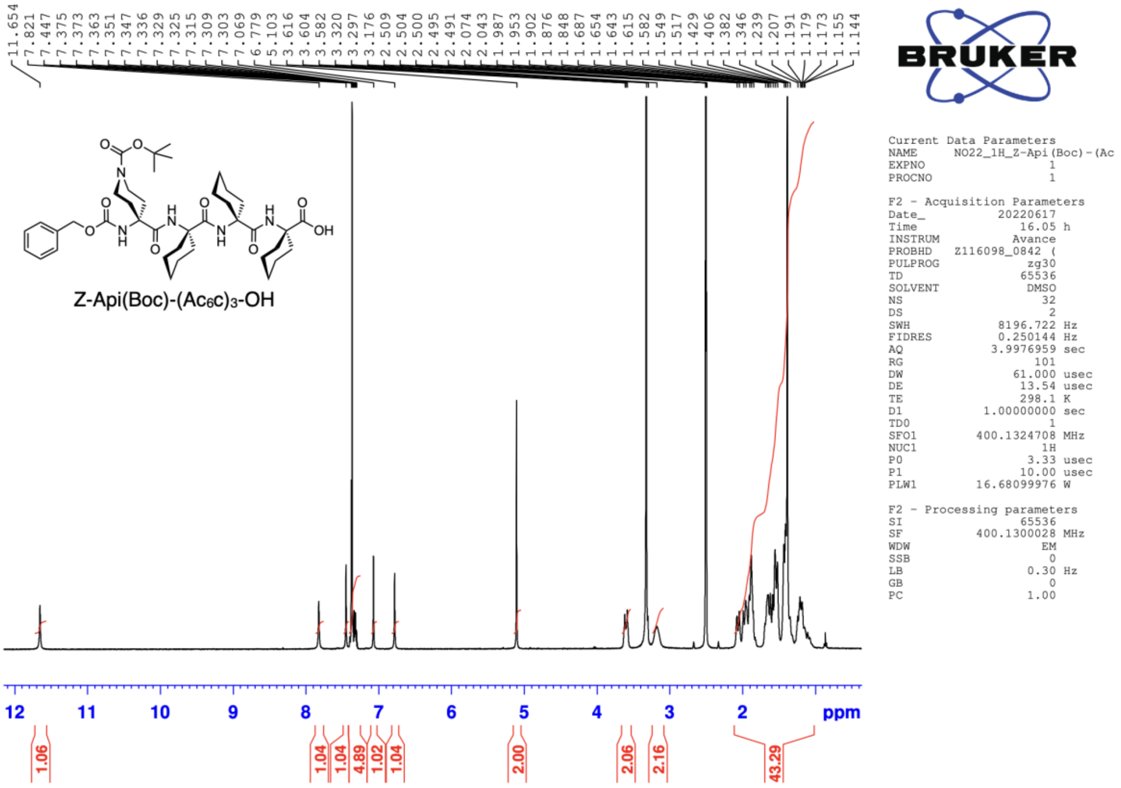

^{1}H NMR (400 MHz, $(CD_3)_2SO$, 25 ºC) spectrum of Z-Api(Boc)-(Ac$_6$c)$_3$-OH.

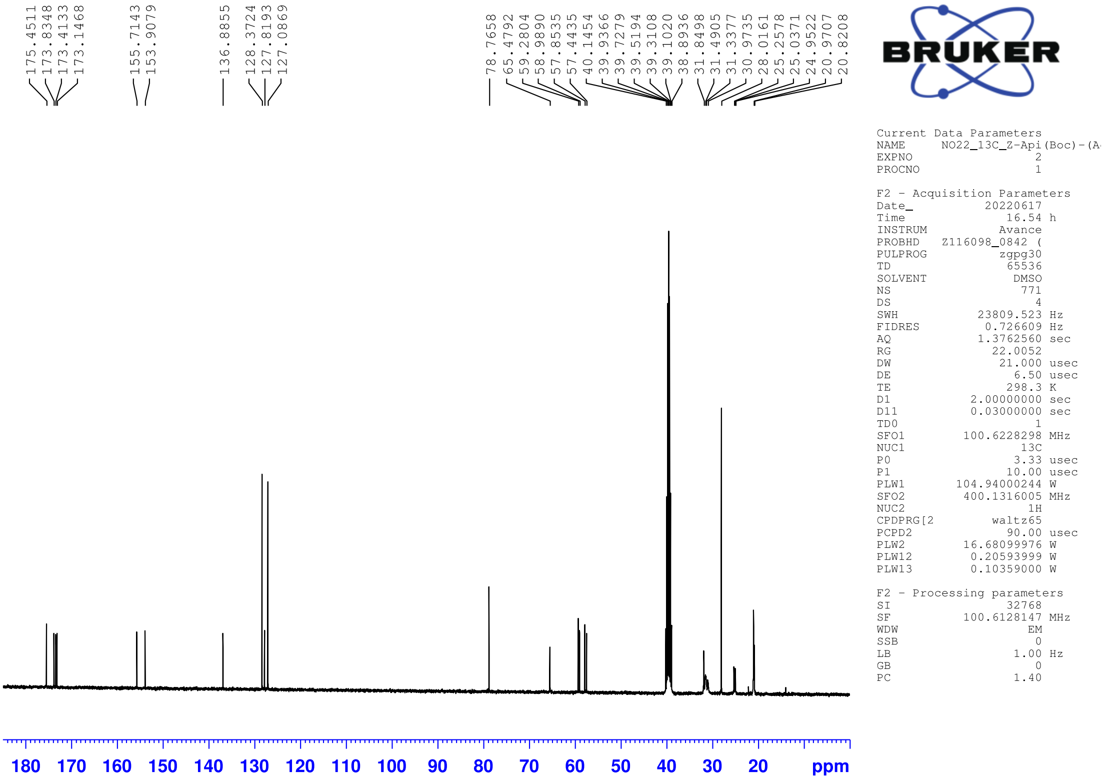

^{13}C NMR (100 MHz, $(CD_3)_2SO$, 25 ºC) spectrum of Z-Api(Boc)-(Ac$_6$c)$_3$-OH.

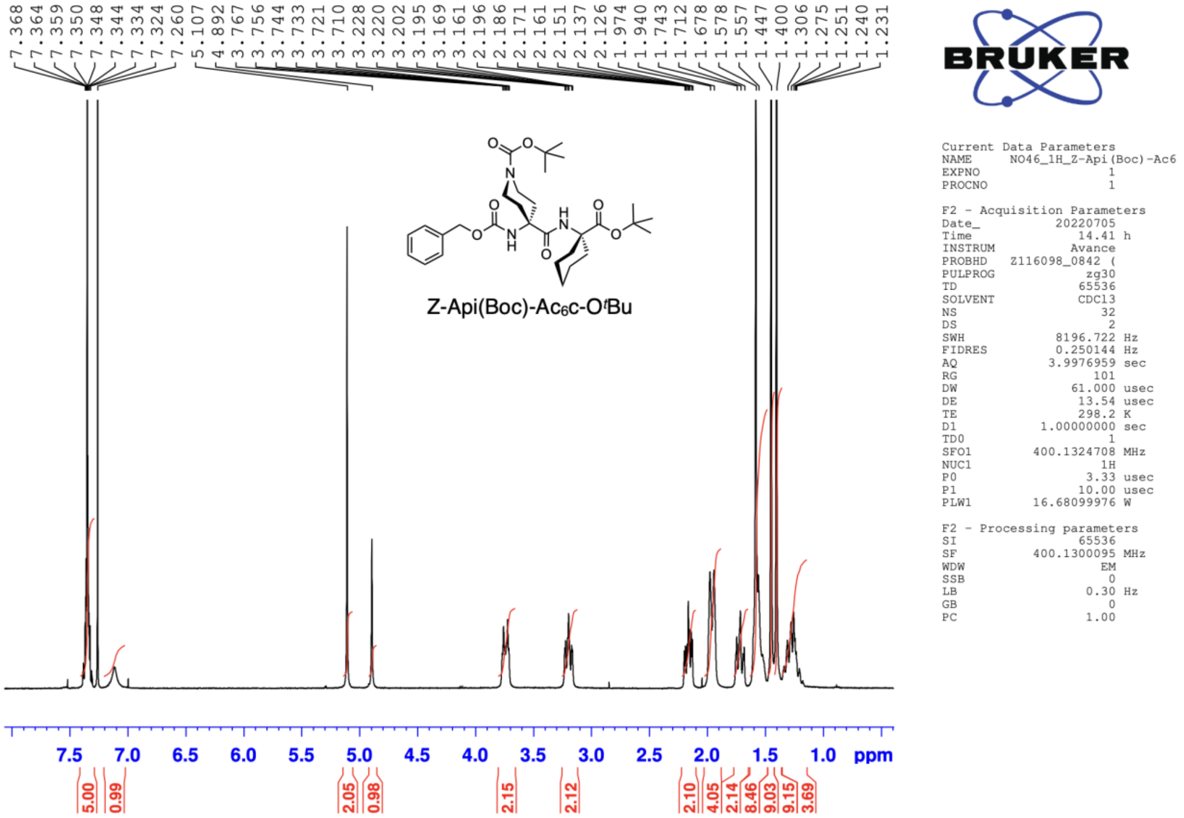

^{1}H NMR (400 MHz, $CDCl_3$, 25 ºC) spectrum of Z-Api(Boc)-Ac$_6$c-O^{t}Bu.

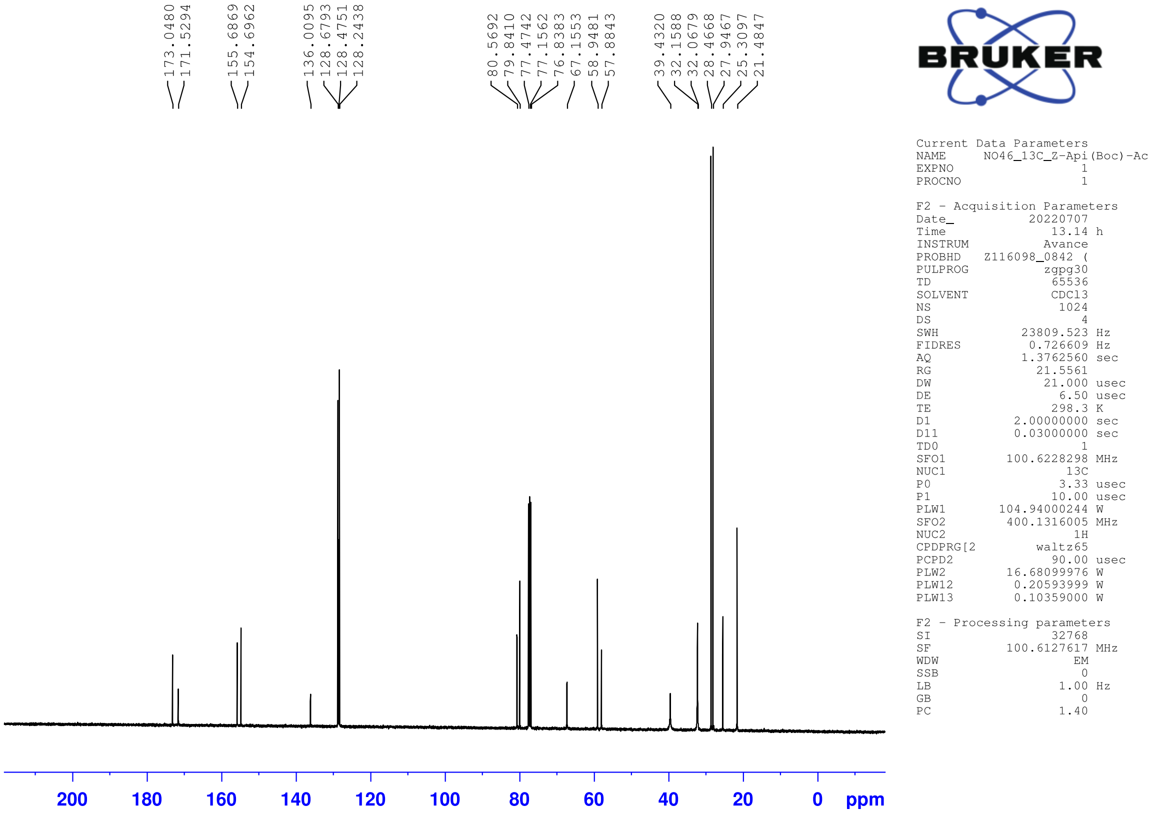

^{13}C NMR (100 MHz, $CDCl_3$, 25 ºC) spectrum of Z-Api(Boc)-Ac$_6$c-O^{t}Bu.

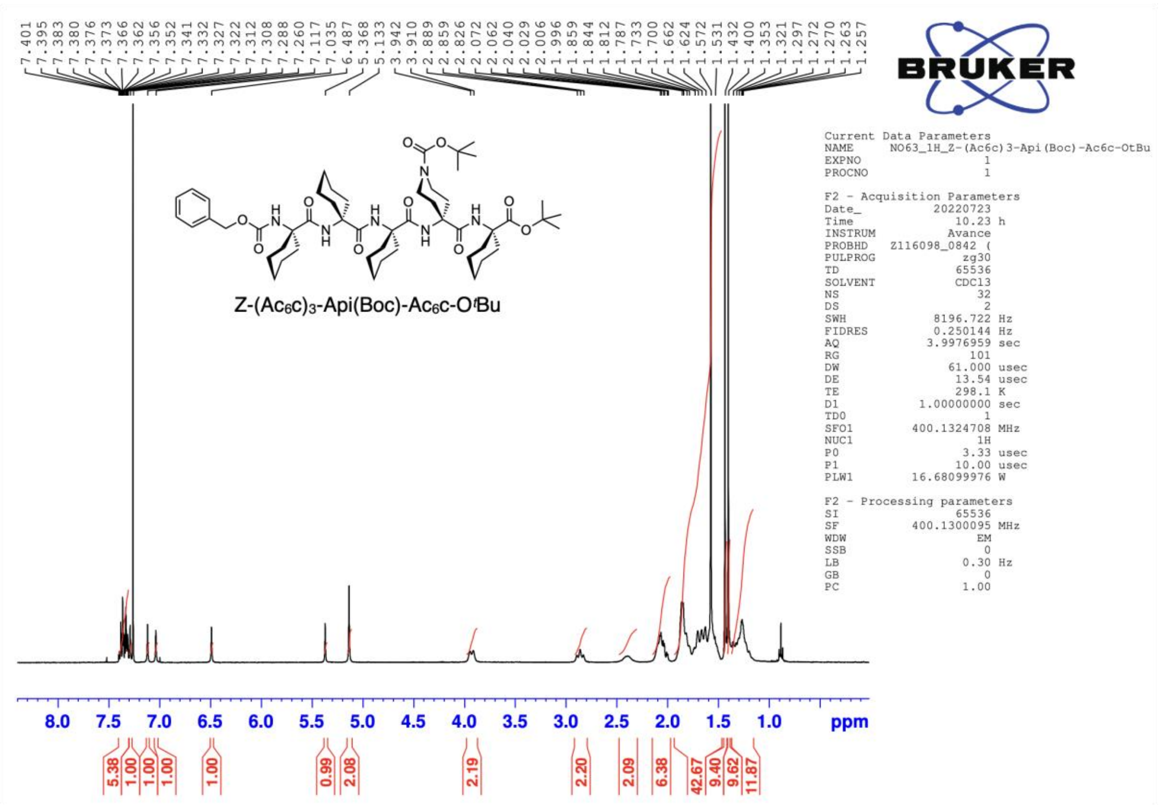

^{1}H NMR (400 MHz, $CDCl_3$, 25 ºC) spectrum of Z-(Ac$_6$c)$_3$-Api(Boc)-Ac$_6$c-O^{t}Bu.

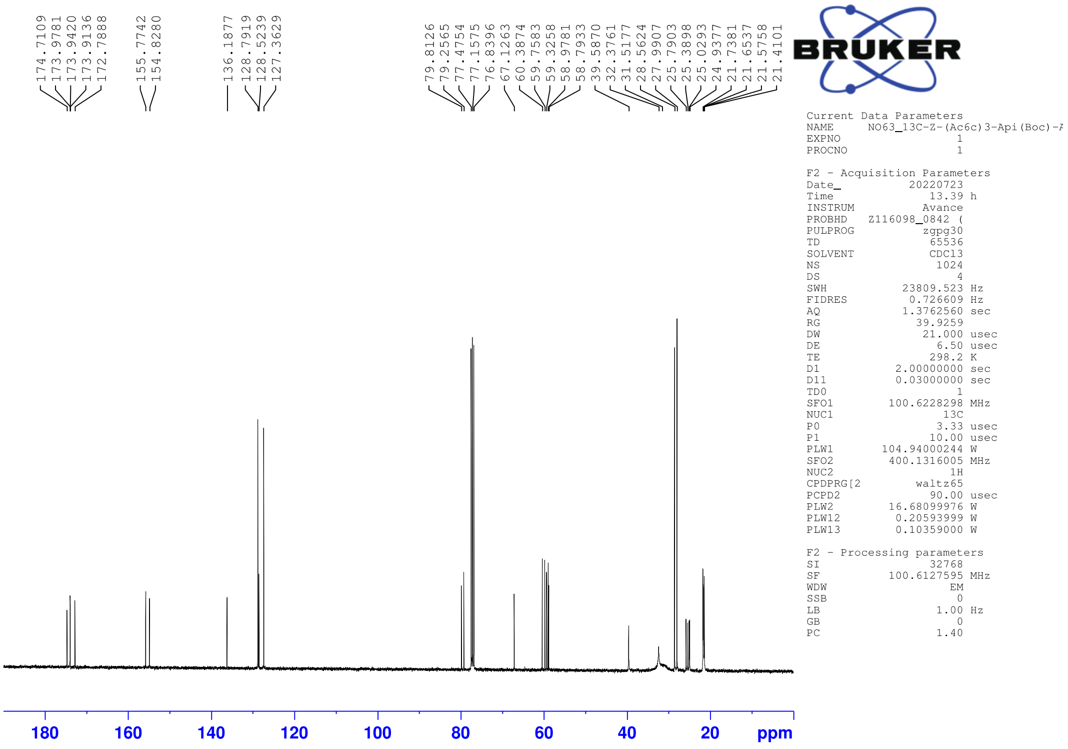

^{13}C NMR (100 MHz, $CDCl_3$, 25 ºC) spectrum of Z-(Ac$_6$c)$_3$-Api(Boc)-Ac$_6$c-O^{t}Bu.

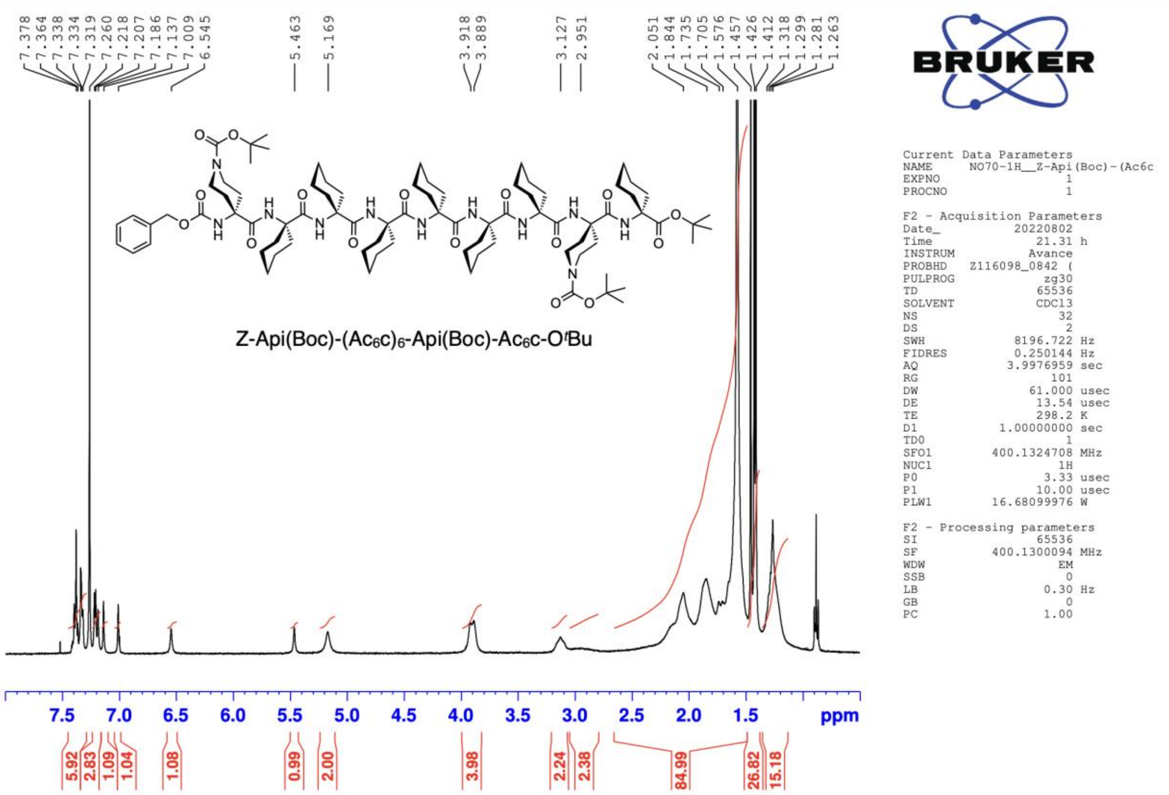

^{1}H NMR (400 MHz, CDCl$_3$, 25 ºC) spectrum of Z-Api(Boc)-(Ac$_6$c)$_6$-Api(Boc)-Ac$_6$c-O^{t}Bu.

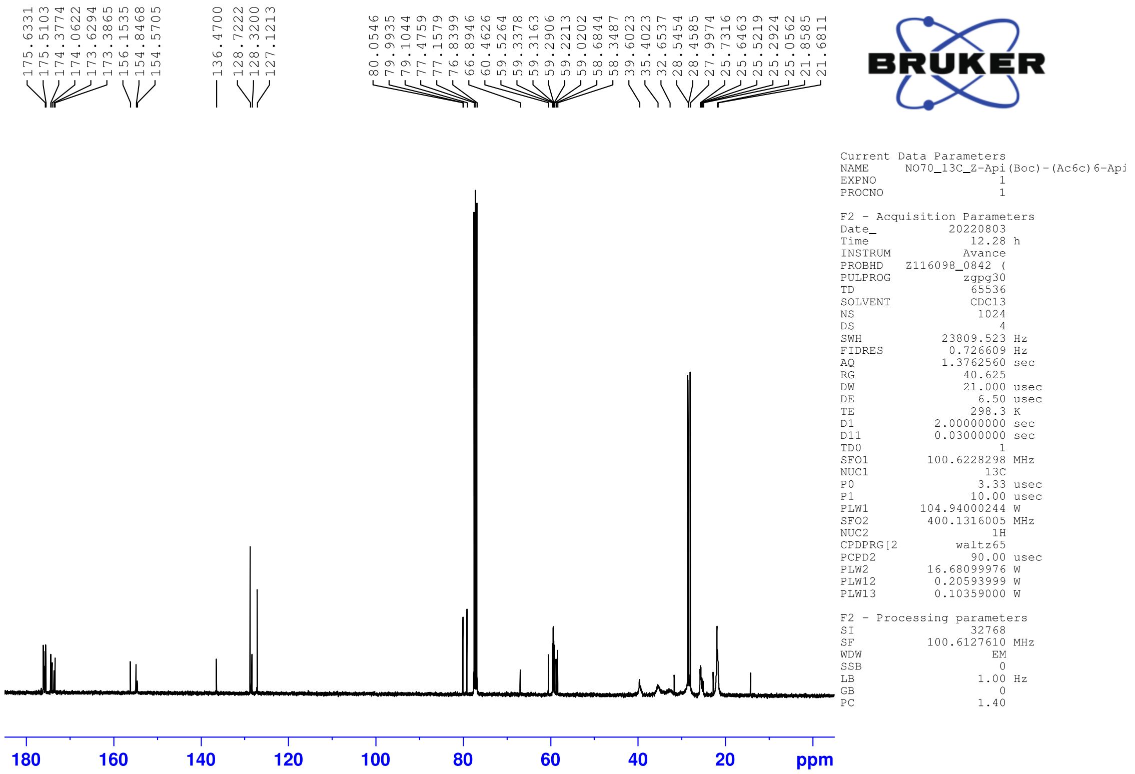

^{13}C NMR (100 MHz, CDCl$_3$, 25 ºC) spectrum of Z-Api(Boc)-(Ac$_6$c)$_6$-Api(Boc)-Ac$_6$c-O^{t}Bu.

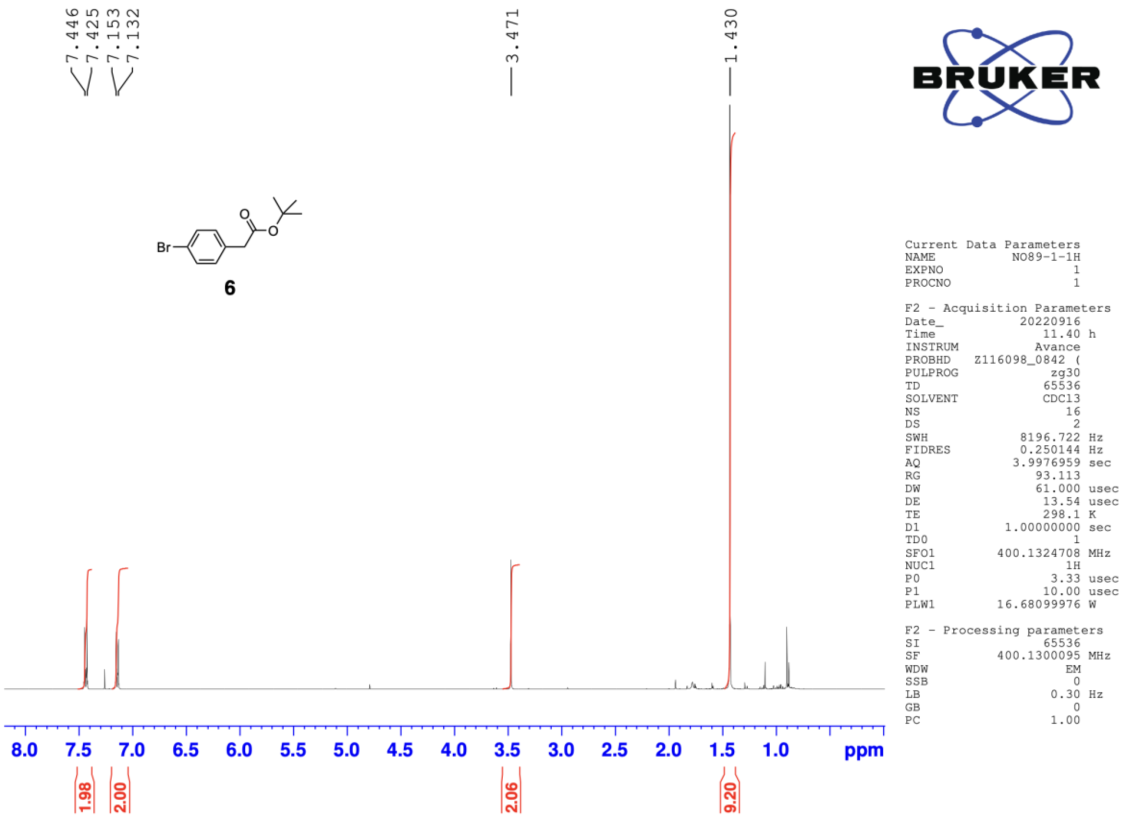

^{1}H NMR (400 MHz, CDCl$_3$, 25 ºC) spectrum of **6**.

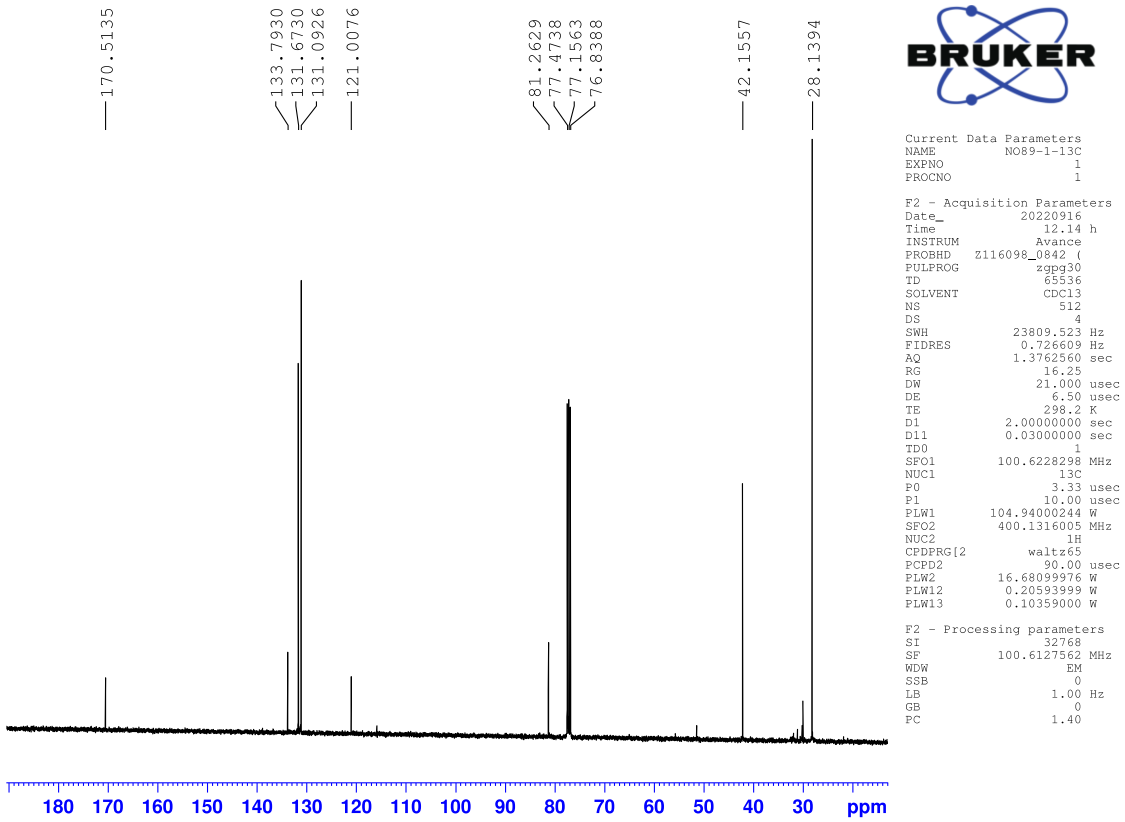

^{13}C NMR (100 MHz, CDCl$_3$, 25 ºC) spectrum of **6**.

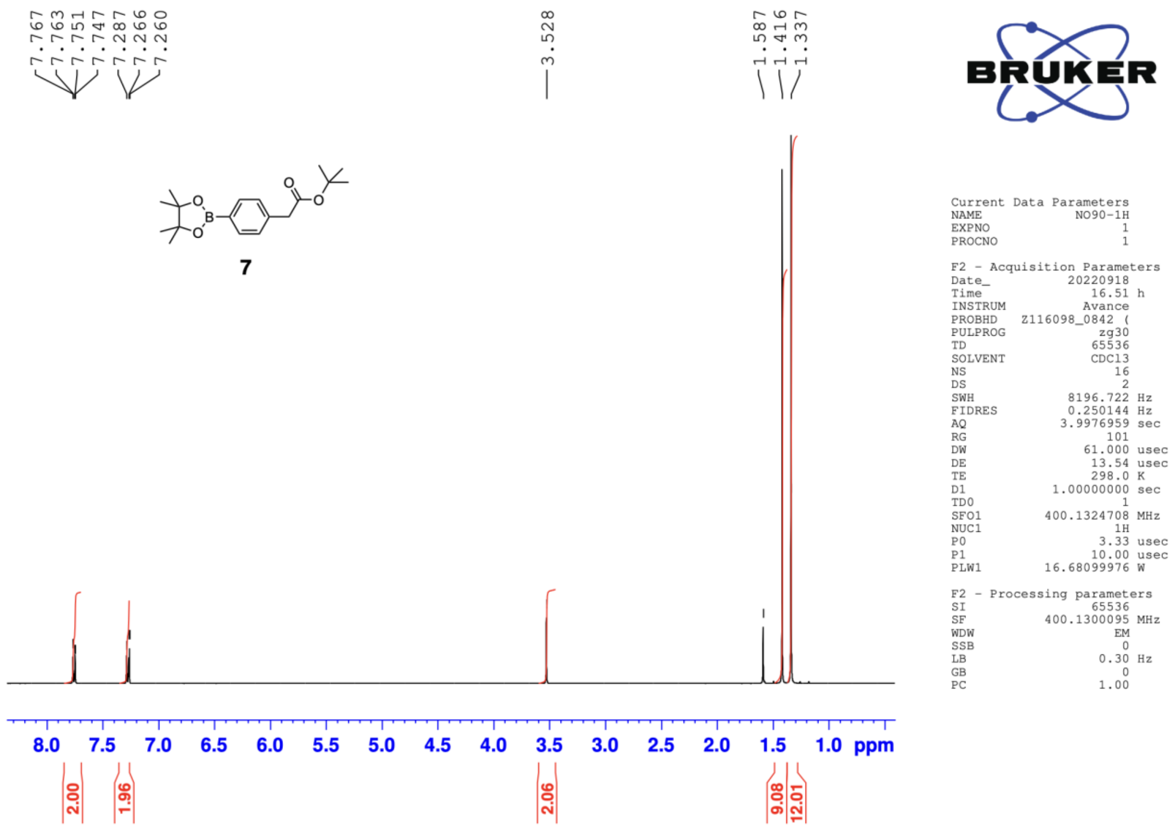

^{1}H NMR (400 MHz, $CDCl_3$, 25 ºC) spectrum of **7**.

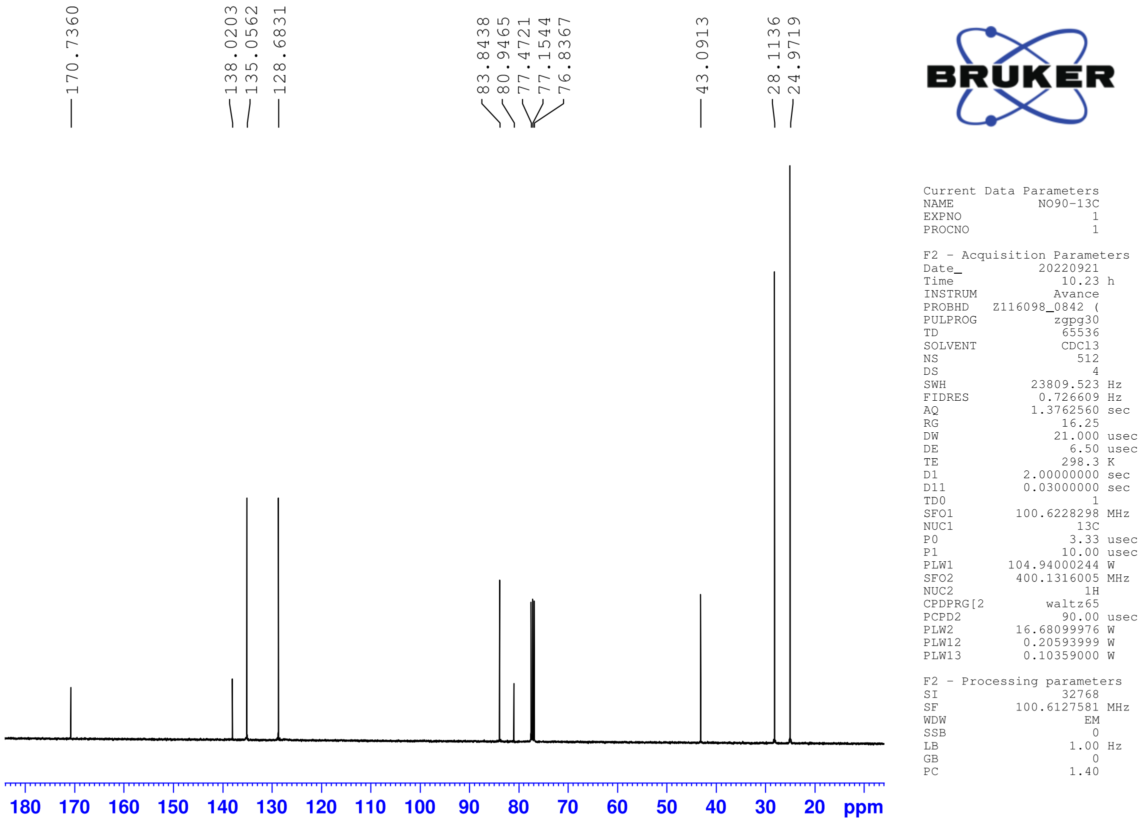

^{13}C NMR (100 MHz, $CDCl_3$, 25 ºC) spectrum of **7**.

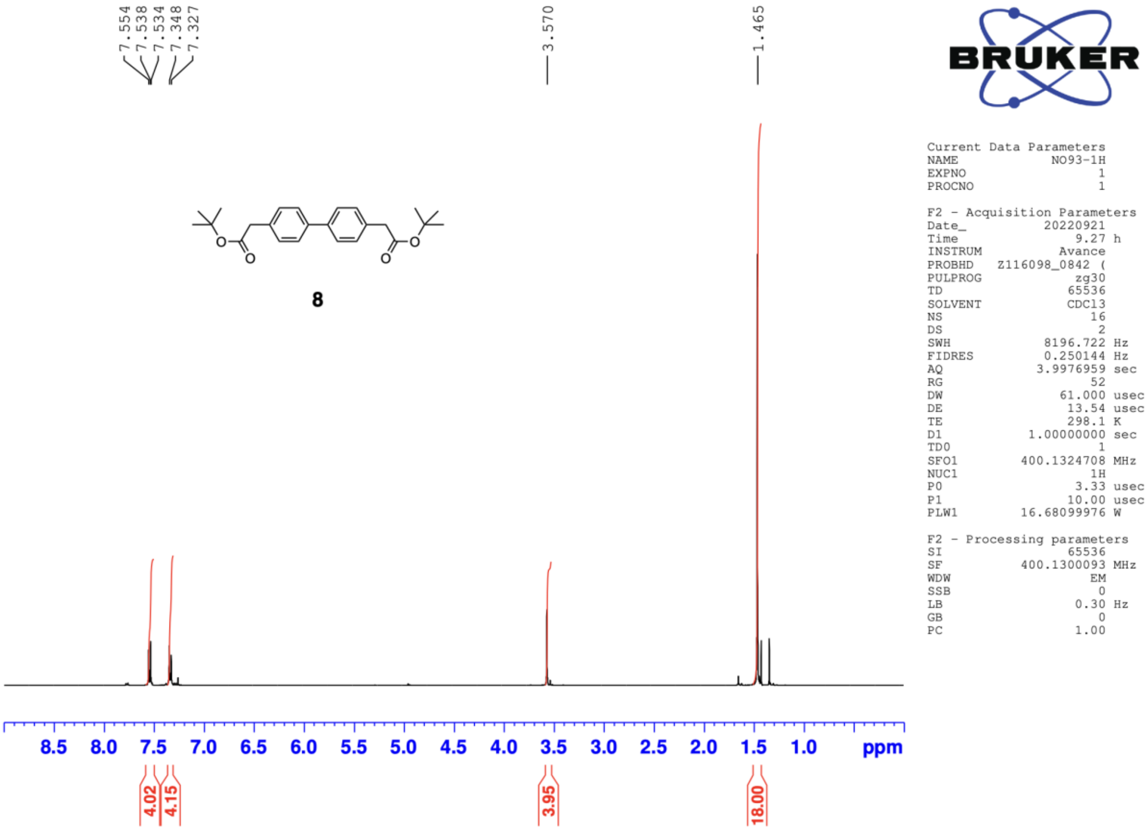

^{1}H NMR (400 MHz, CDCl$_3$, 25 ºC) spectrum of **8**.

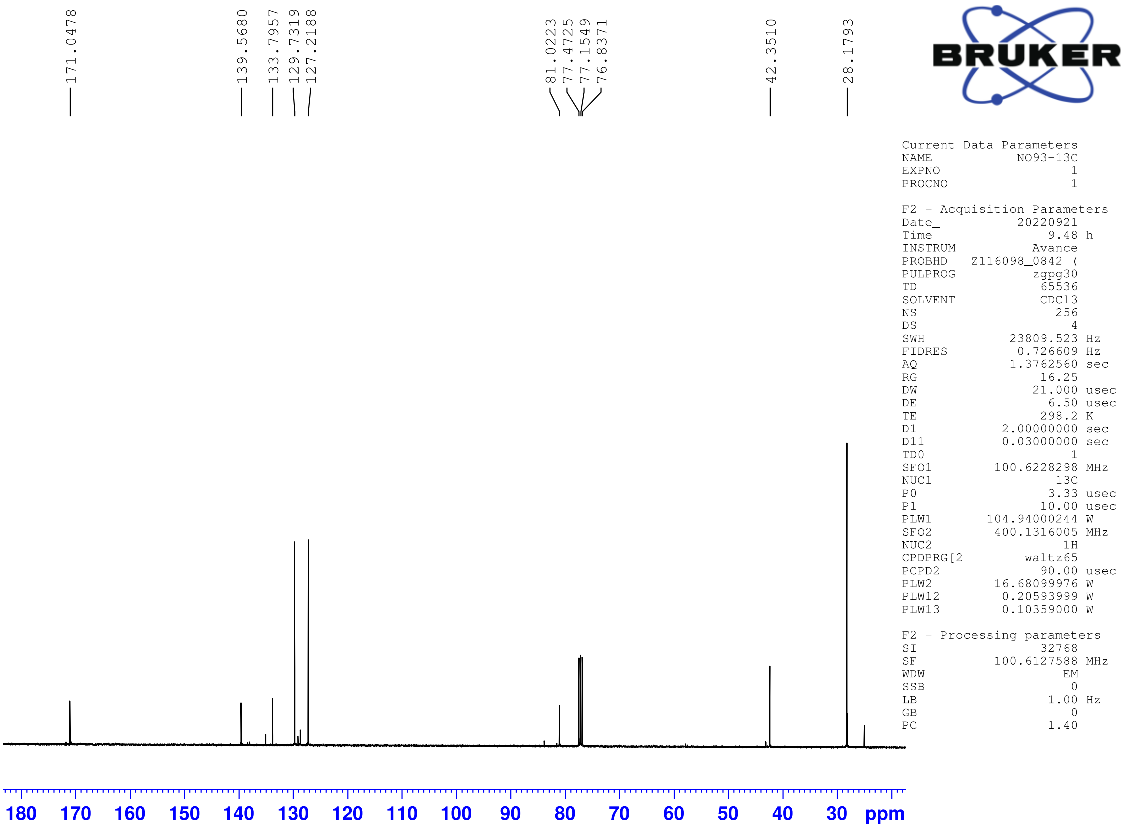

^{13}C NMR (100 MHz, CDCl$_3$, 25 ºC) spectrum of **8**.

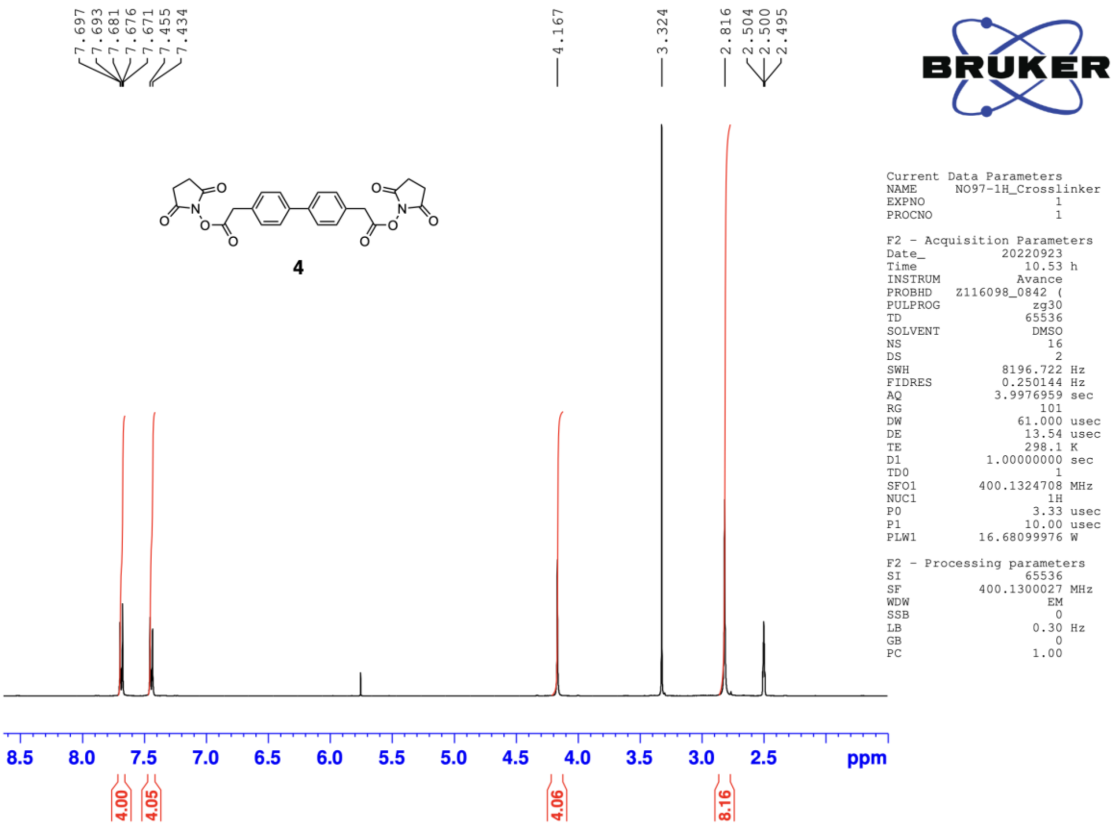

^{1}H NMR (400 MHz, $(CD_3)_2SO$, 25 ºC) spectrum of **4**.

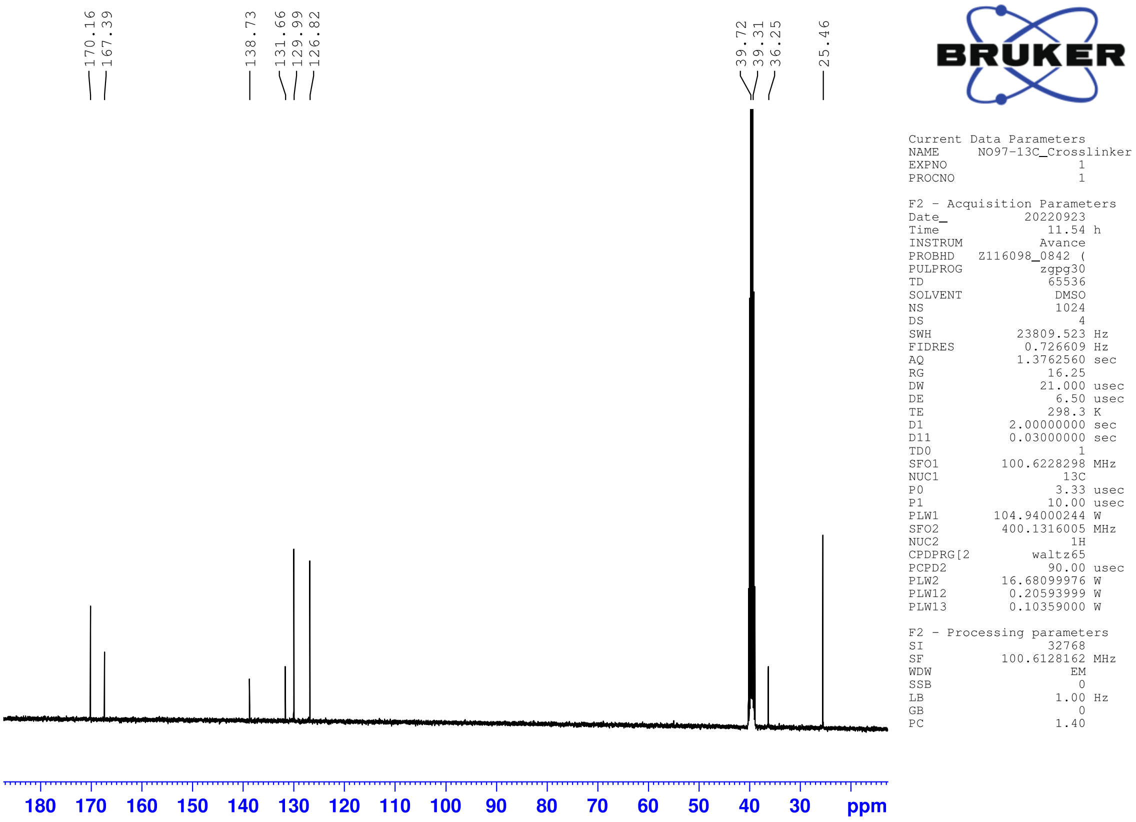

^{13}C NMR (100 MHz, $(CD_3)_2SO$, 25 ºC) spectrum of **4**.

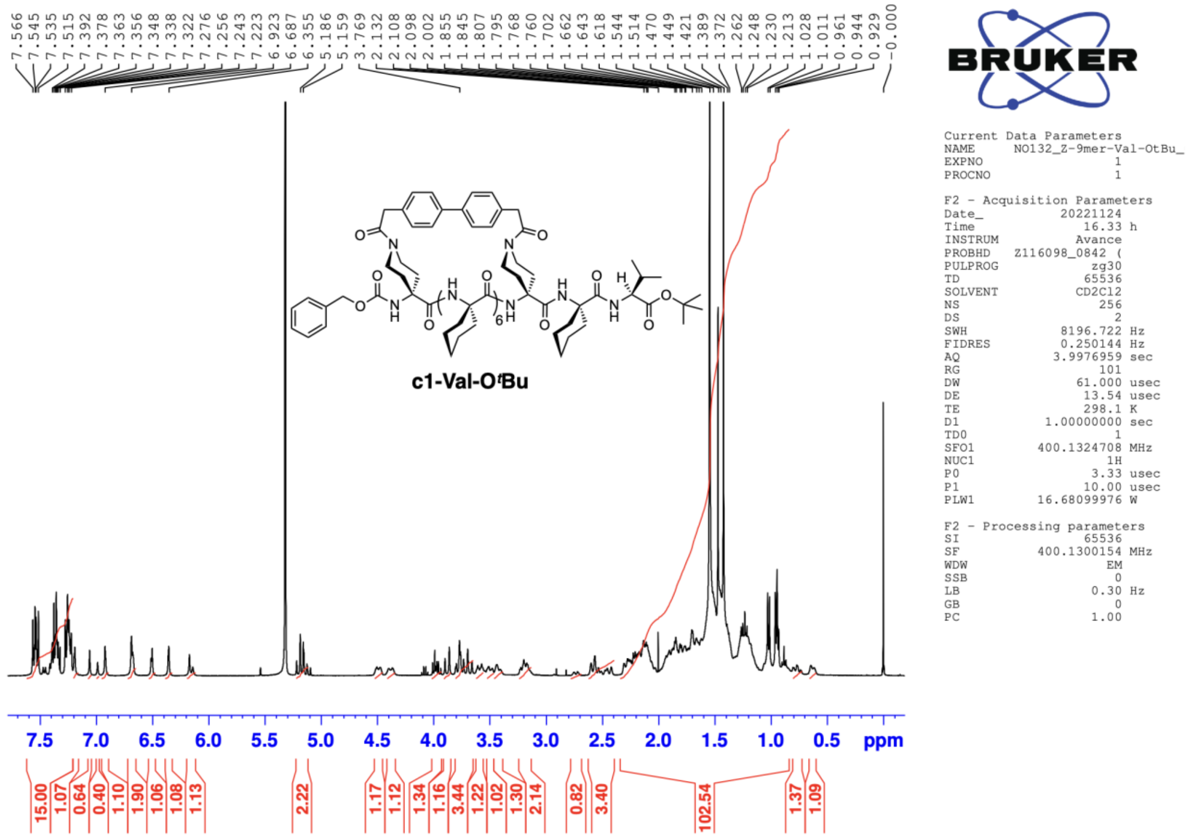

^{1}H NMR (400 MHz, CD_2Cl_2, 25 ºC) spectrum of **c1-Val-O*t*Bu**.

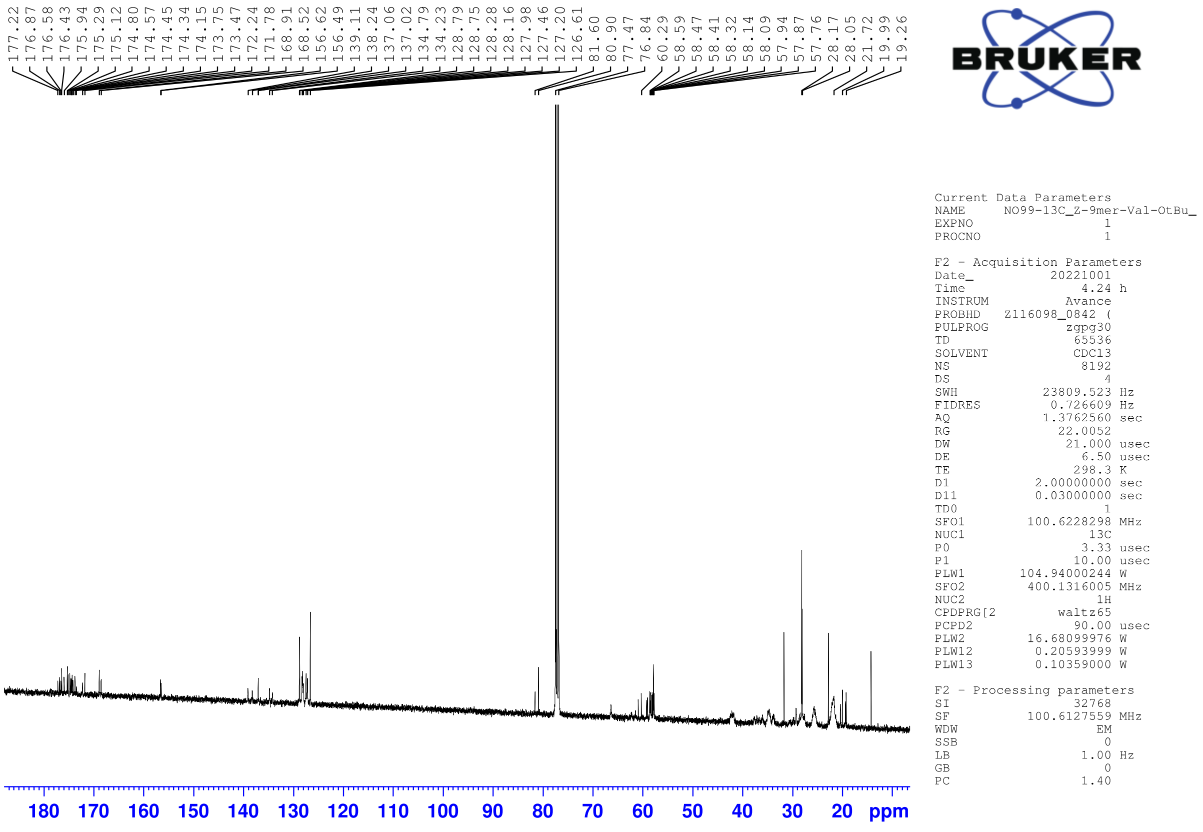

^{13}C NMR (100 MHz, CD_2Cl_2, 25 ºC) spectrum of **c1-Val-O*t*Bu**.

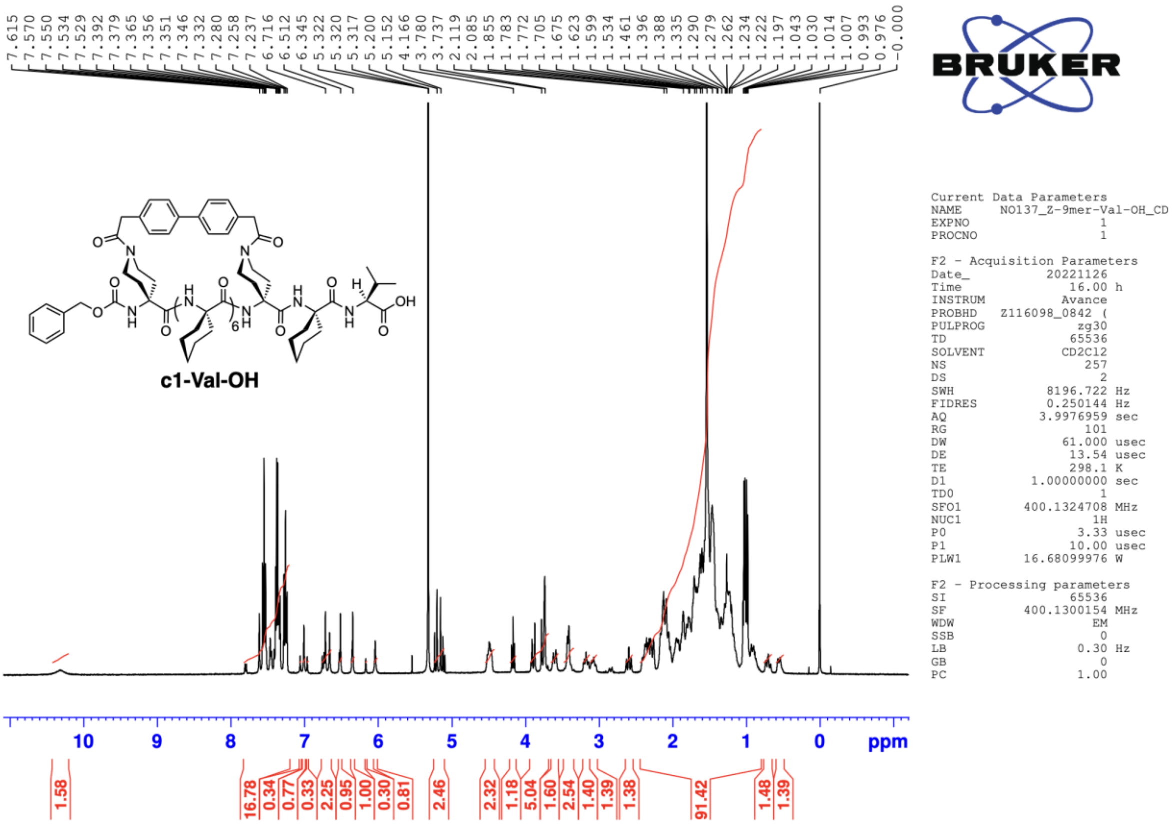

^{1}H NMR (400 MHz, CD_2Cl_2, 25 ºC) spectrum of **c1-Val-OH**.

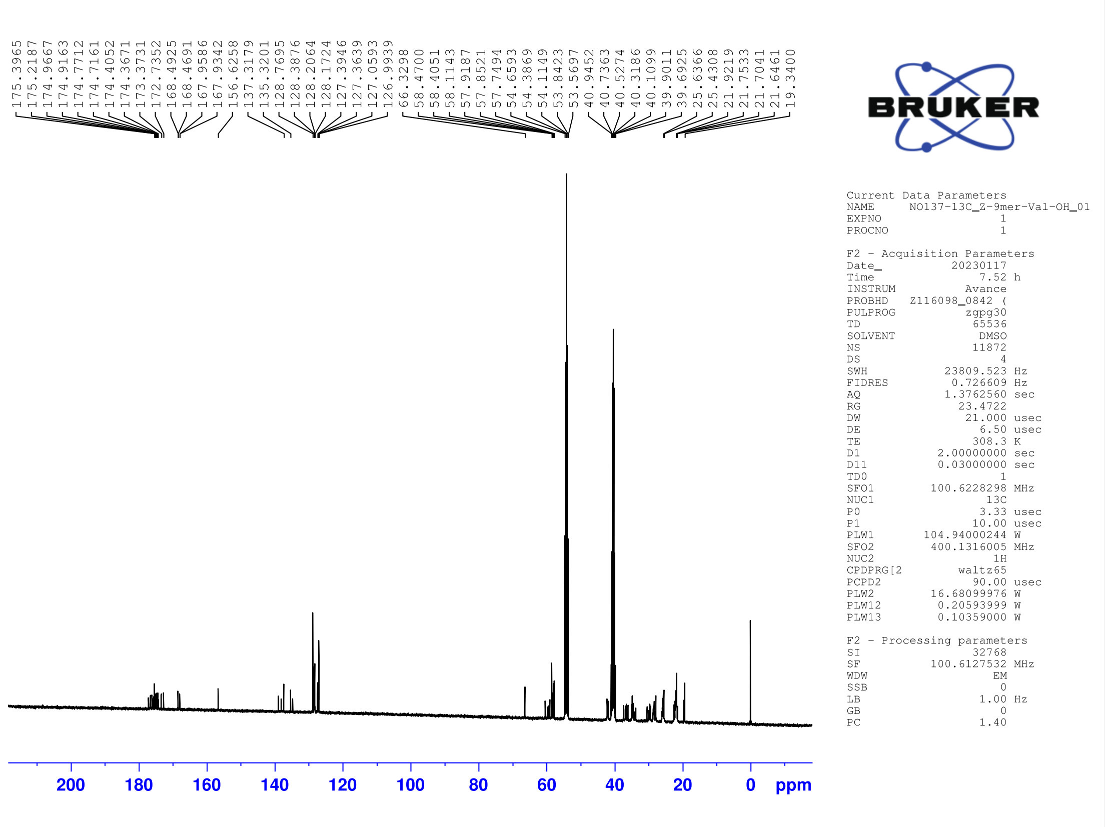

^{13}C NMR (100 MHz, $CD_2Cl_2/(CD_3)_2SO$ (5/2, v/v), 25 ºC) spectrum of **c1-Val-OH**.

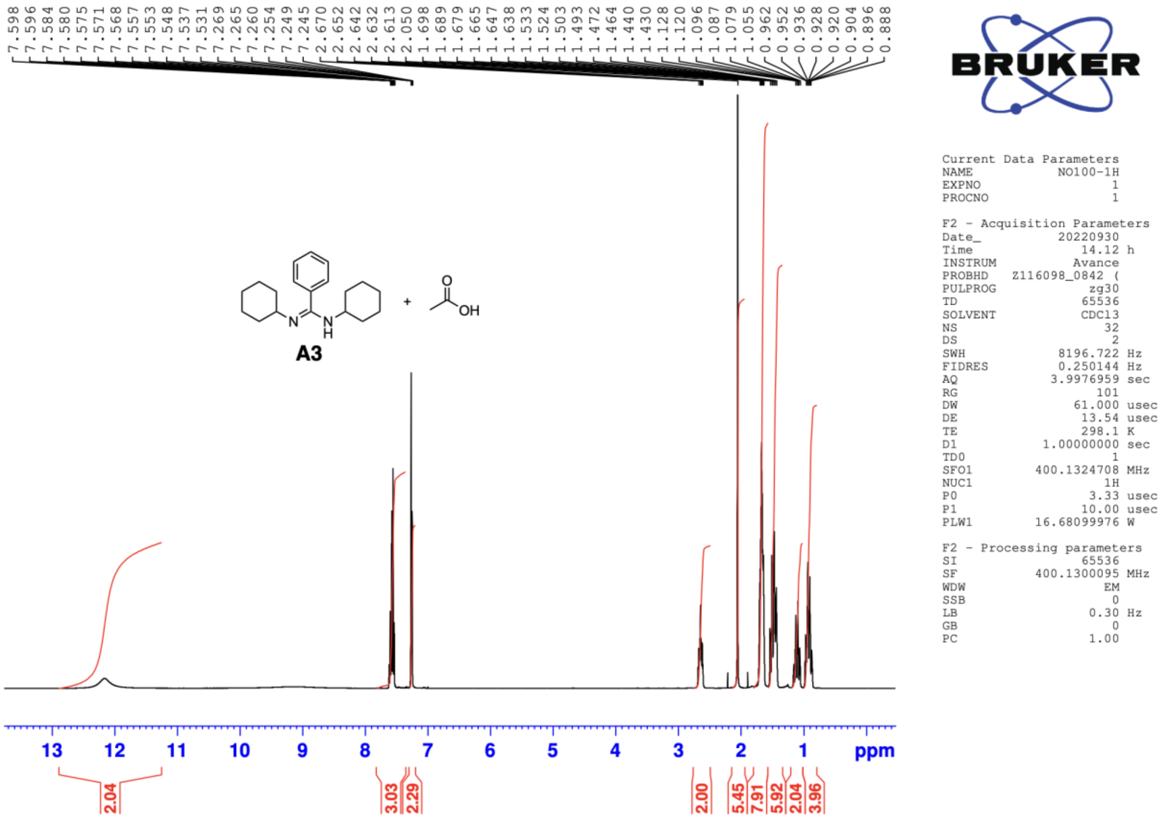

^{1}H NMR (400 MHz, $CDCl_3$, 25 ºC) spectrum of **A3**·(CH_3CO_2H).

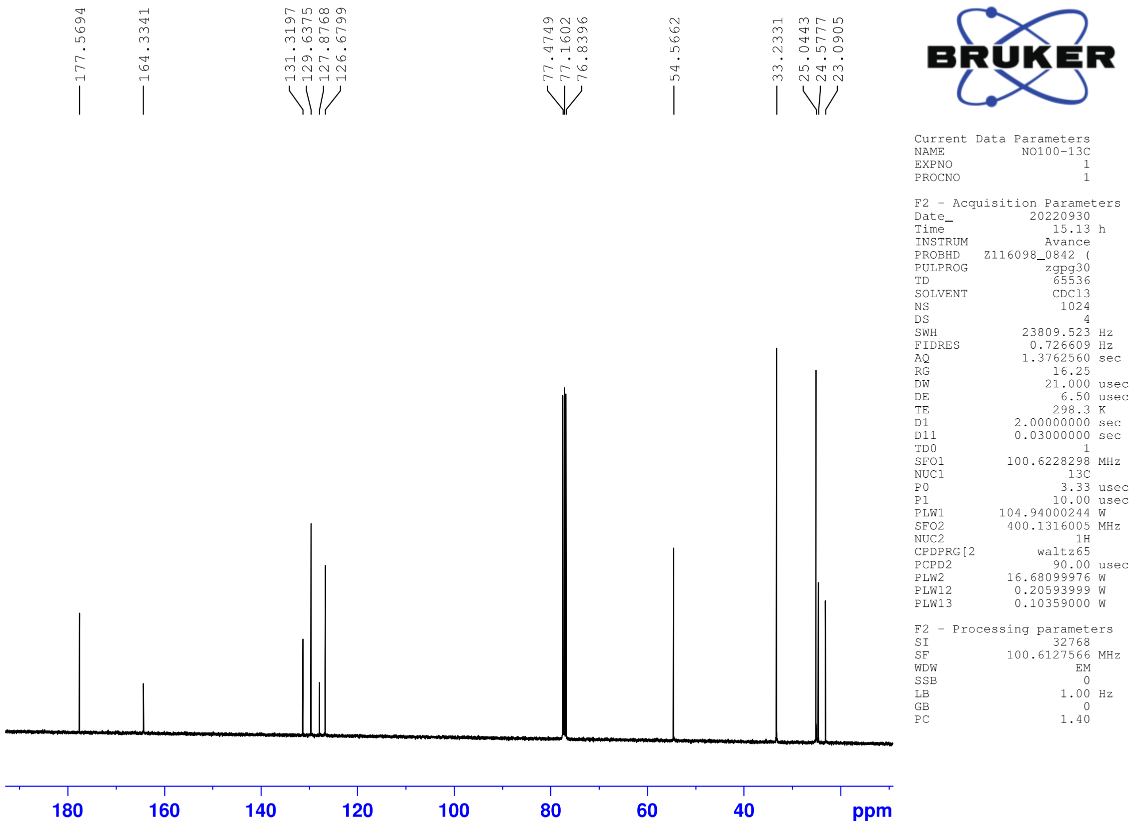

^{13}C NMR (100 MHz, $CDCl_3$, 25 ºC) spectrum of **A3**·(CH_3CO_2H).

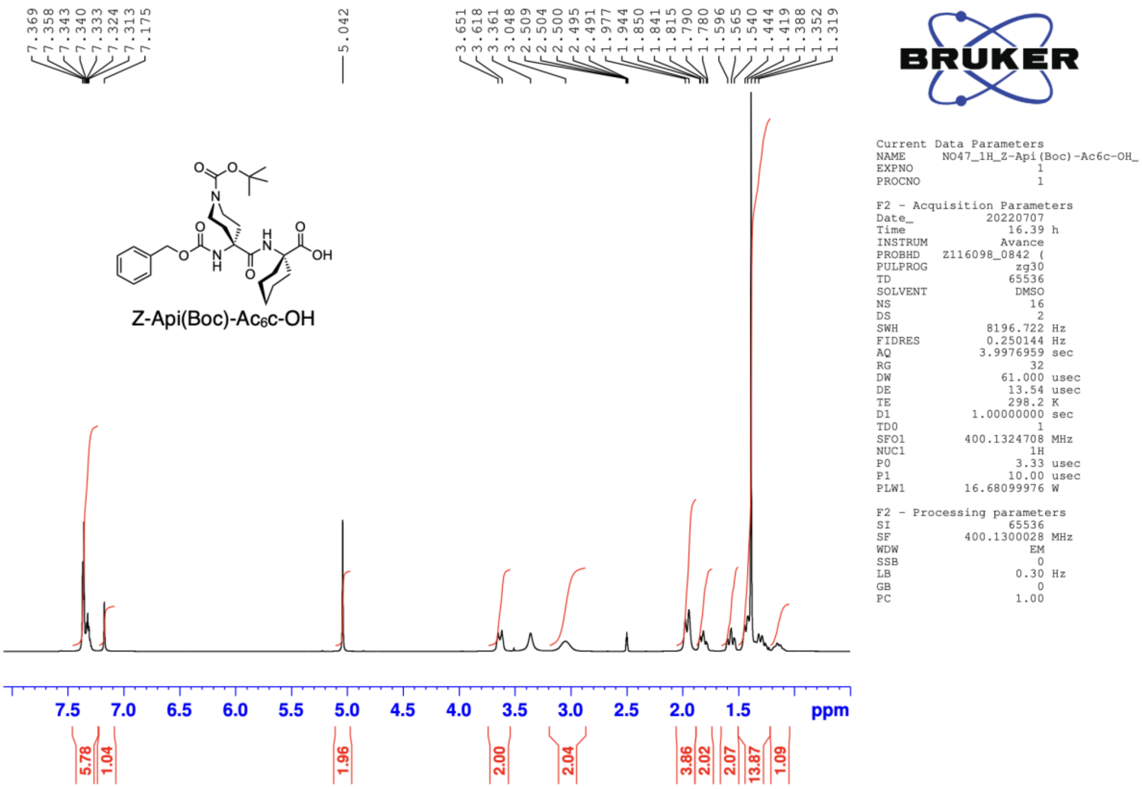

^{1}H NMR (400 MHz, $(CD_3)_2SO$, 25 ºC) spectrum of Z-Api(Boc)-Ac$_6$c-OH.

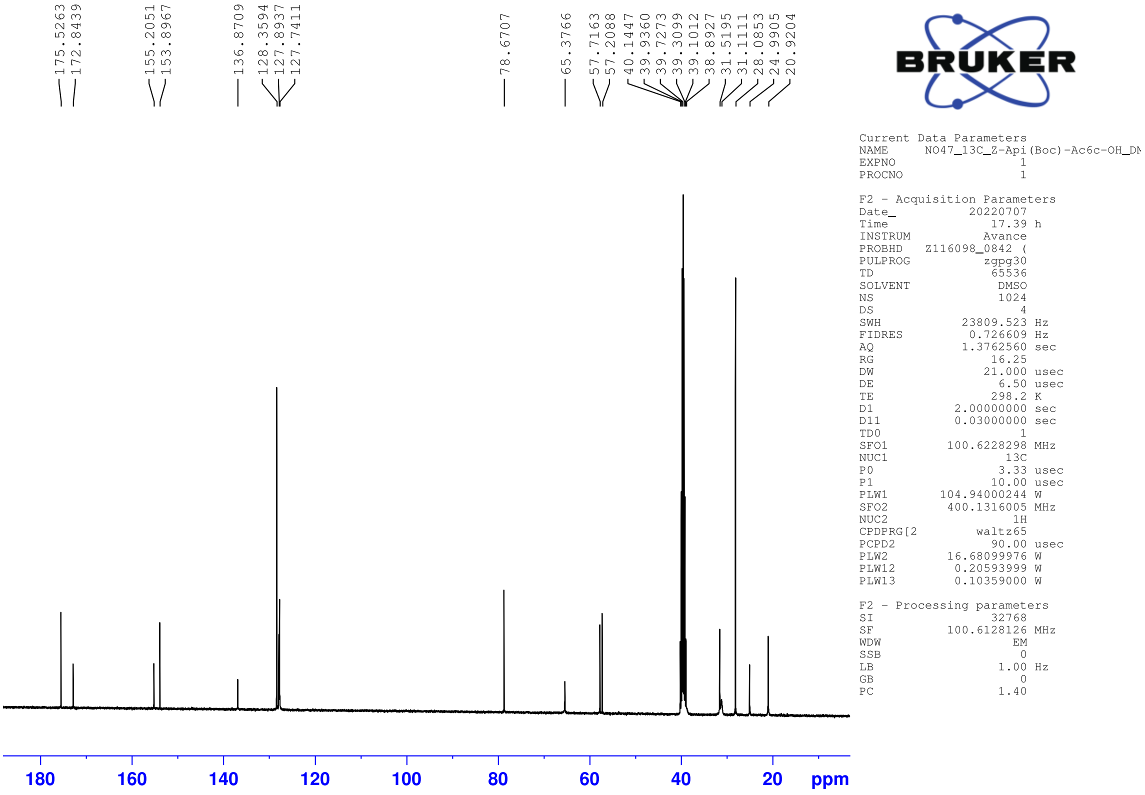

^{13}C NMR (100 MHz, $(CD_3)_2SO$, 25 ºC) spectrum of Z-Api(Boc)-Ac$_6$c-OH.

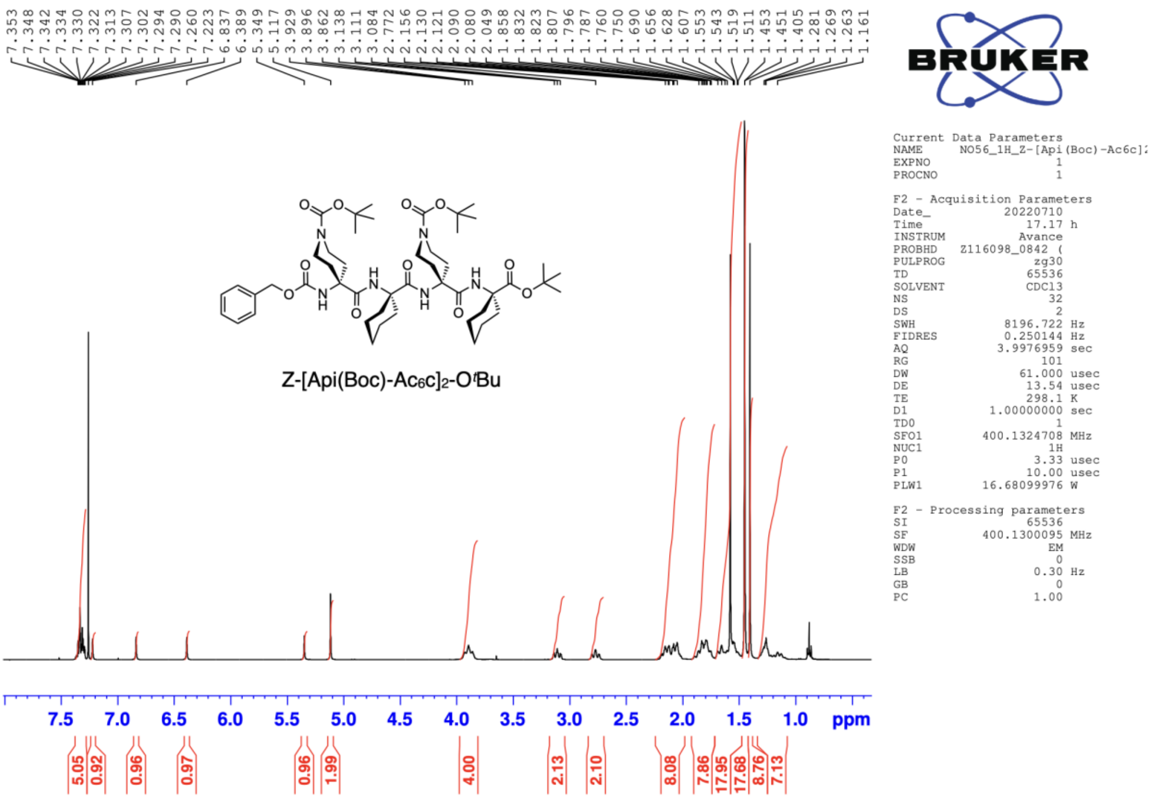

^{1}H NMR (400 MHz, $CDCl_3$, 25 ºC) spectrum of Z-[Api(Boc)-Ac$_6$c]$_2$-O^{t}Bu.

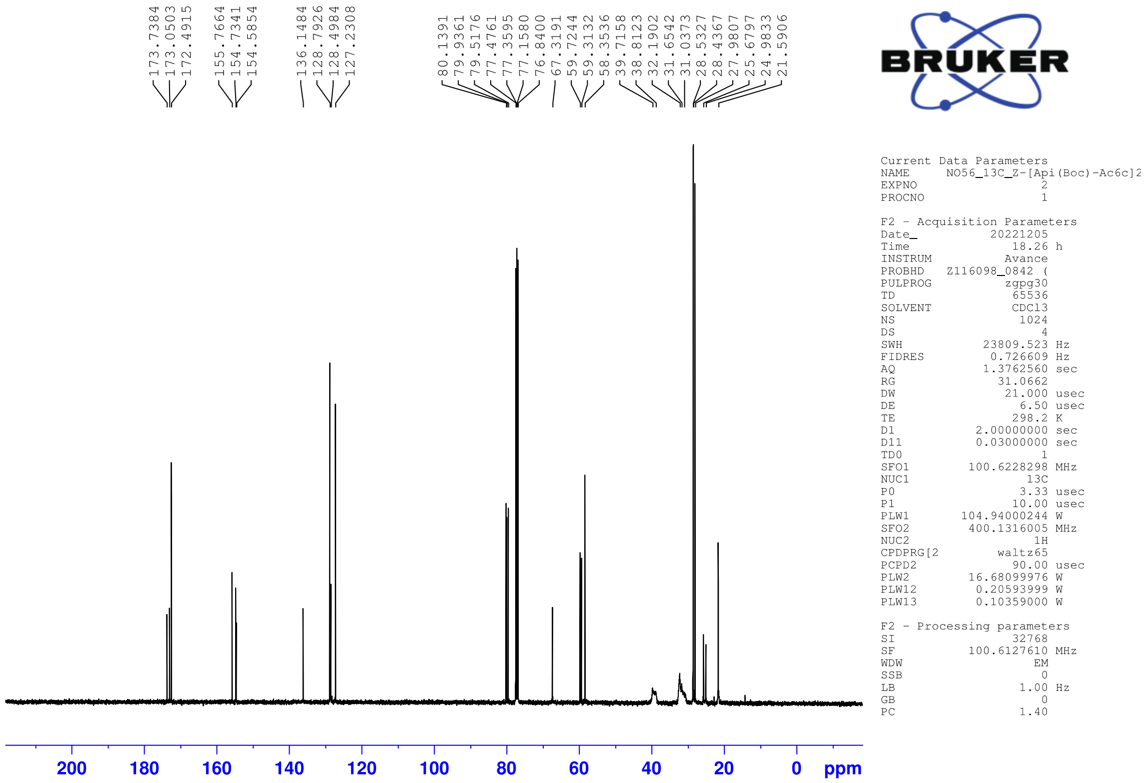

^{13}C NMR (100 MHz, $CDCl_3$, 25 ºC) spectrum of Z-[Api(Boc)-Ac$_6$c]$_2$-O^{t}Bu.

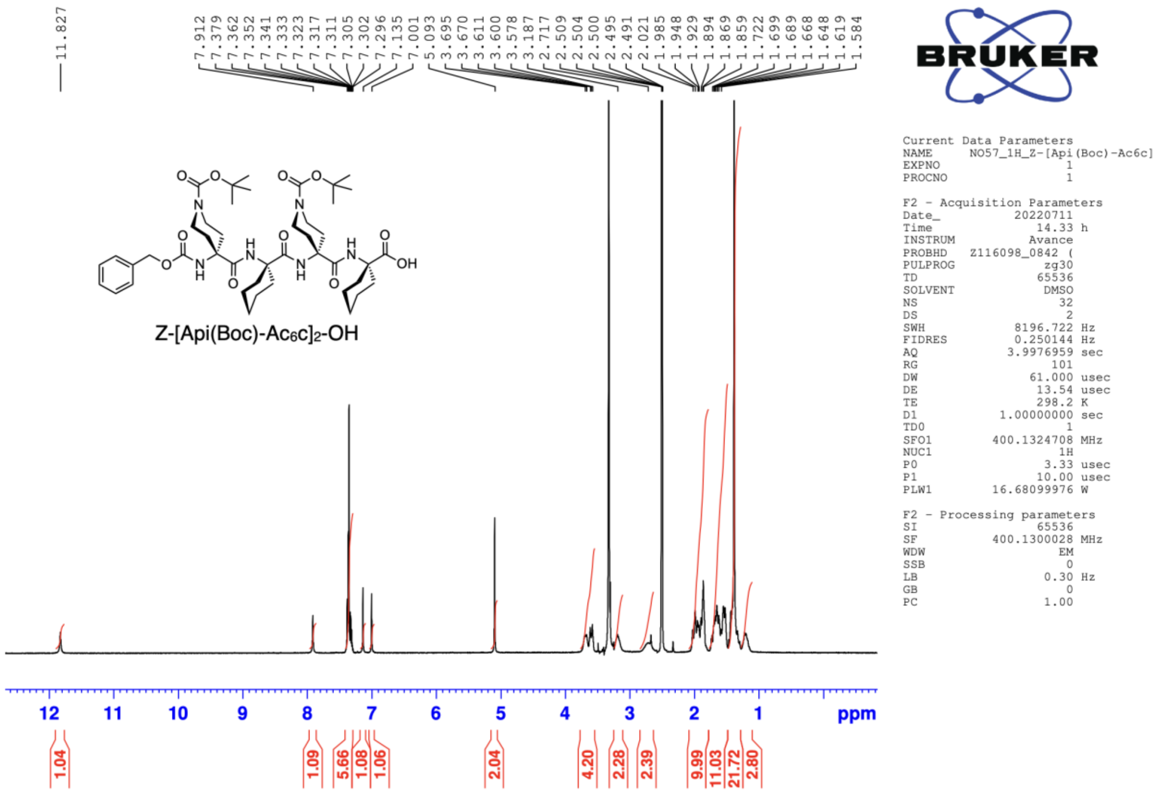

^{1}H NMR (400 MHz, $(CD_3)_2SO$, 25 ºC) spectrum of Z-[Api(Boc)-Ac$_6$c]$_2$-OH.

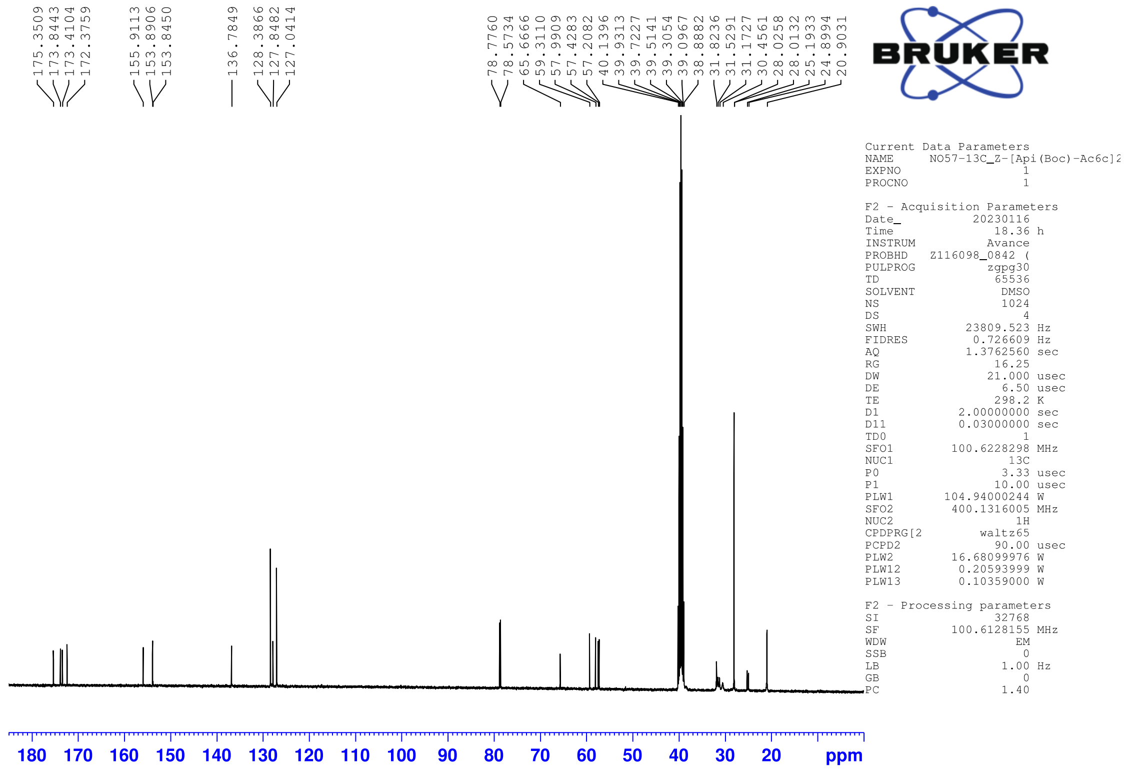

^{13}C NMR (100 MHz, $(CD_3)_2SO$, 25 ºC) spectrum of Z-[Api(Boc)-Ac$_6$c]$_2$-OH.

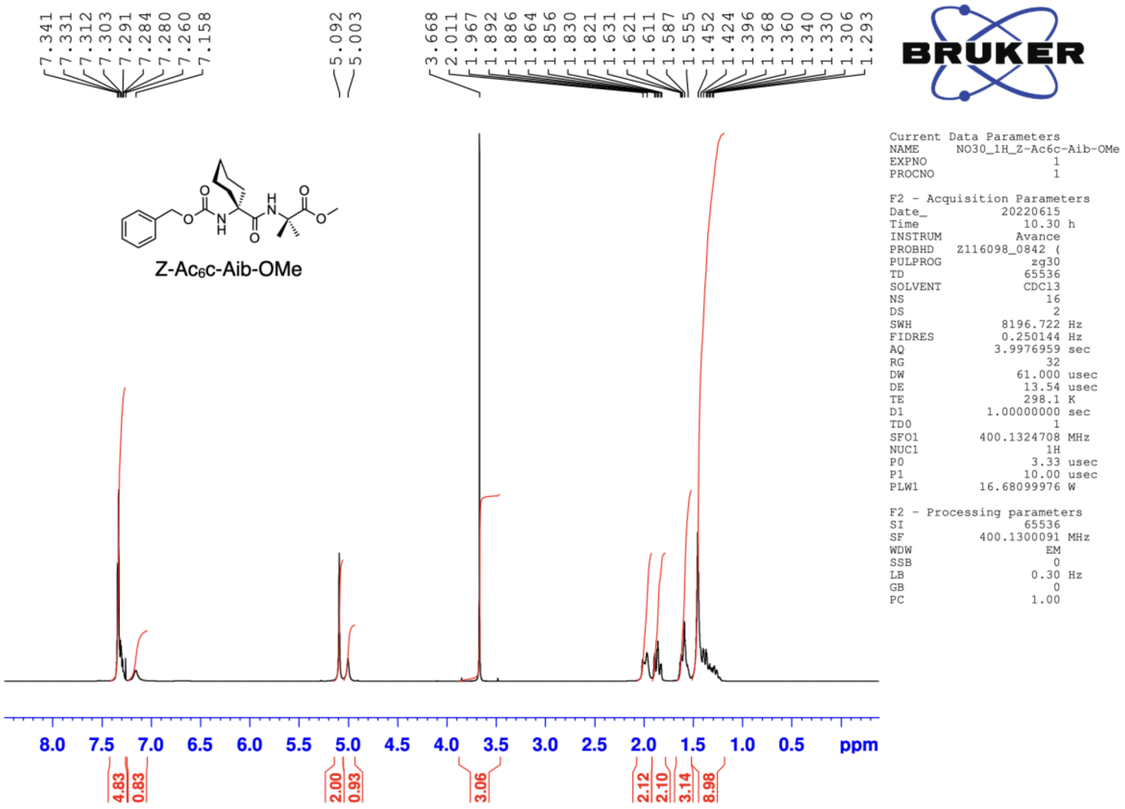

^{1}H NMR (400 MHz, $CDCl_3$, 25 ºC) spectrum of Z-Ac$_6$c-Aib-OMe.

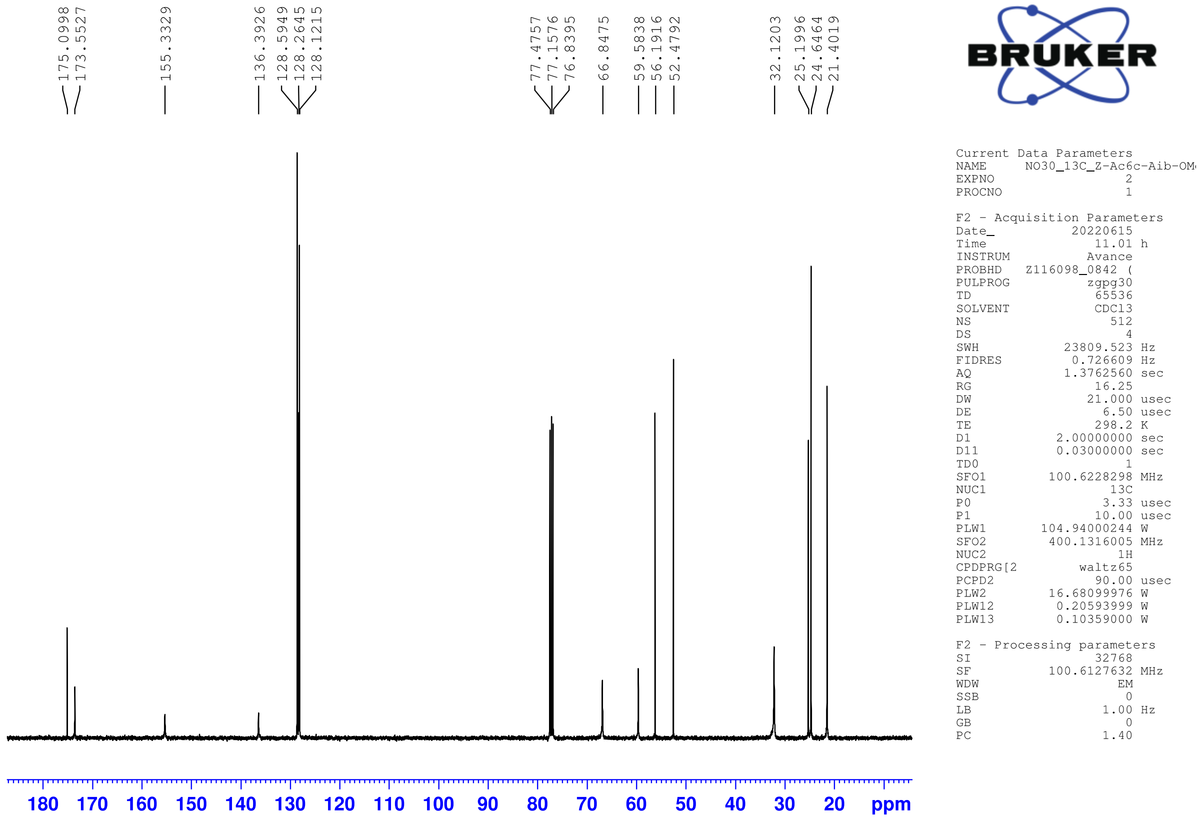

^{13}C NMR (100 MHz, $CDCl_3$, 25 ºC) spectrum of Z-Ac$_6$c-Aib-OMe.

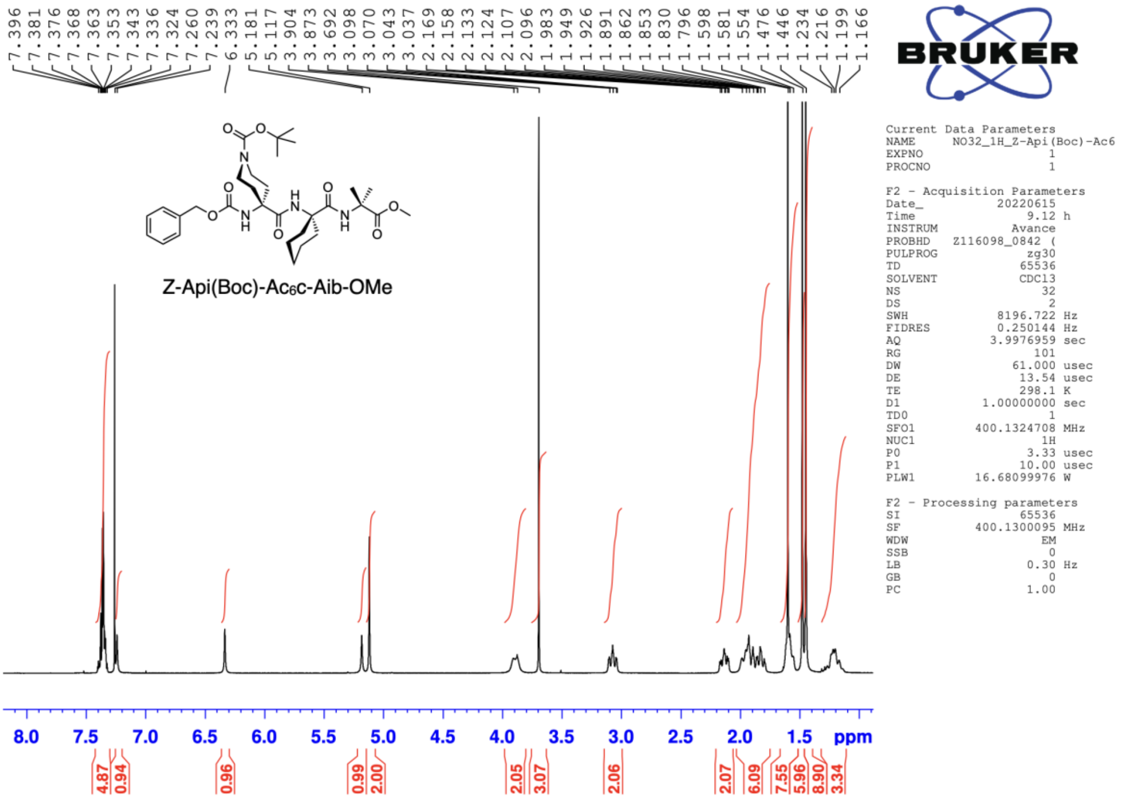

^{1}H NMR (400 MHz, $CDCl_3$, 25 ºC) spectrum of Z-Api(Boc)-Ac$_6$c-Aib-OMe.

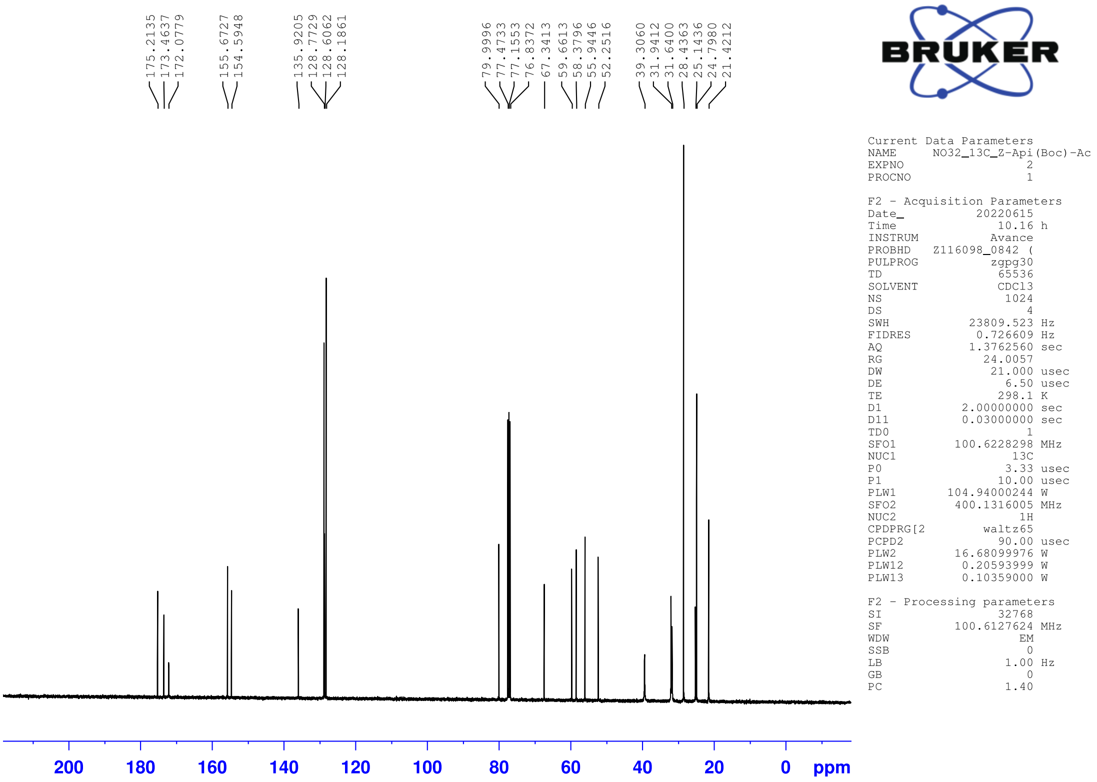

^{13}C NMR (100 MHz, $CDCl_3$, 25 ºC) spectrum of Z-Api(Boc)-Ac$_6$c-Aib-OMe.

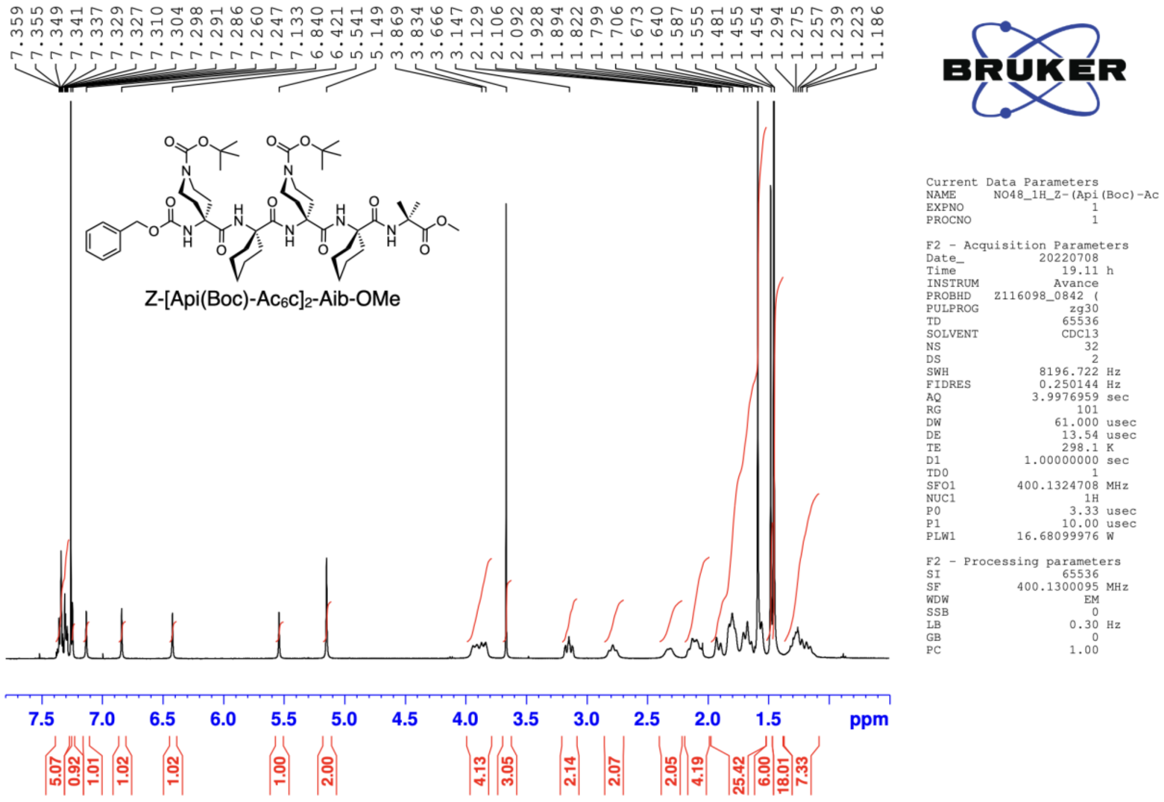

^{1}H NMR (400 MHz, $CDCl_3$, 25 ºC) spectrum of Z-[Api(Boc)-Ac$_6$c]$_2$-Aib-OMe.

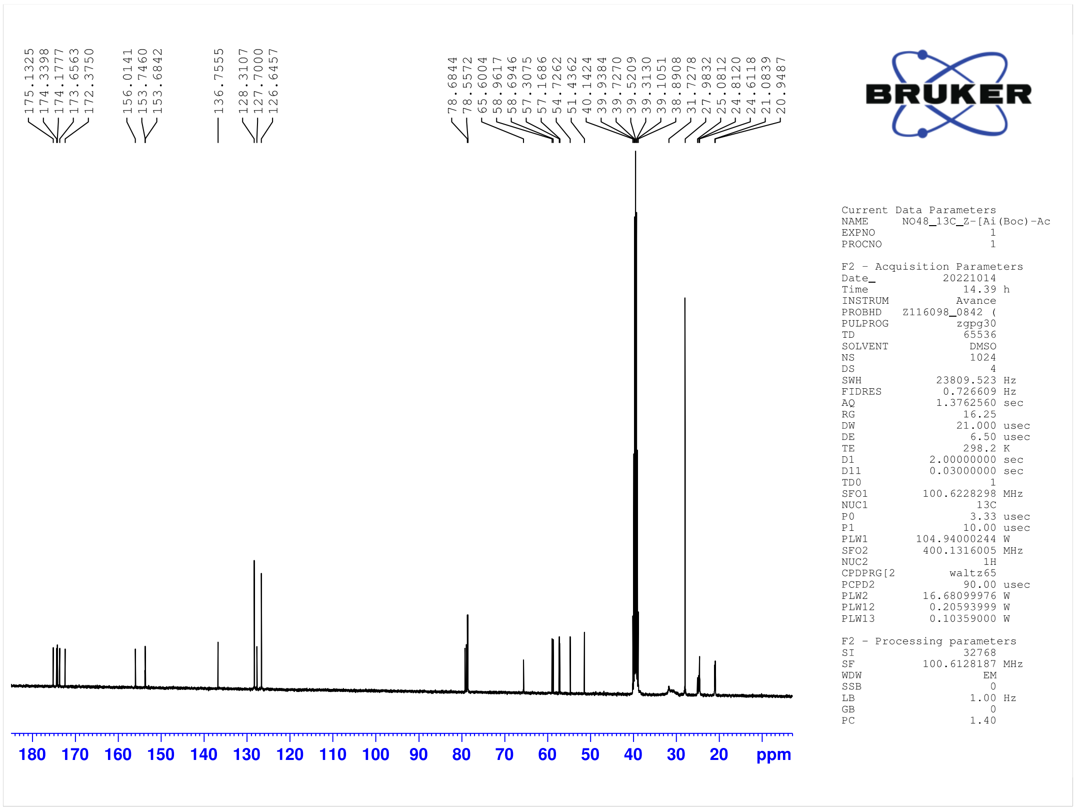

^{13}C NMR (100 MHz, $CDCl_3$, 25 ºC) spectrum of Z-[Api(Boc)-Ac$_6$c]$_2$-Aib-OMe.

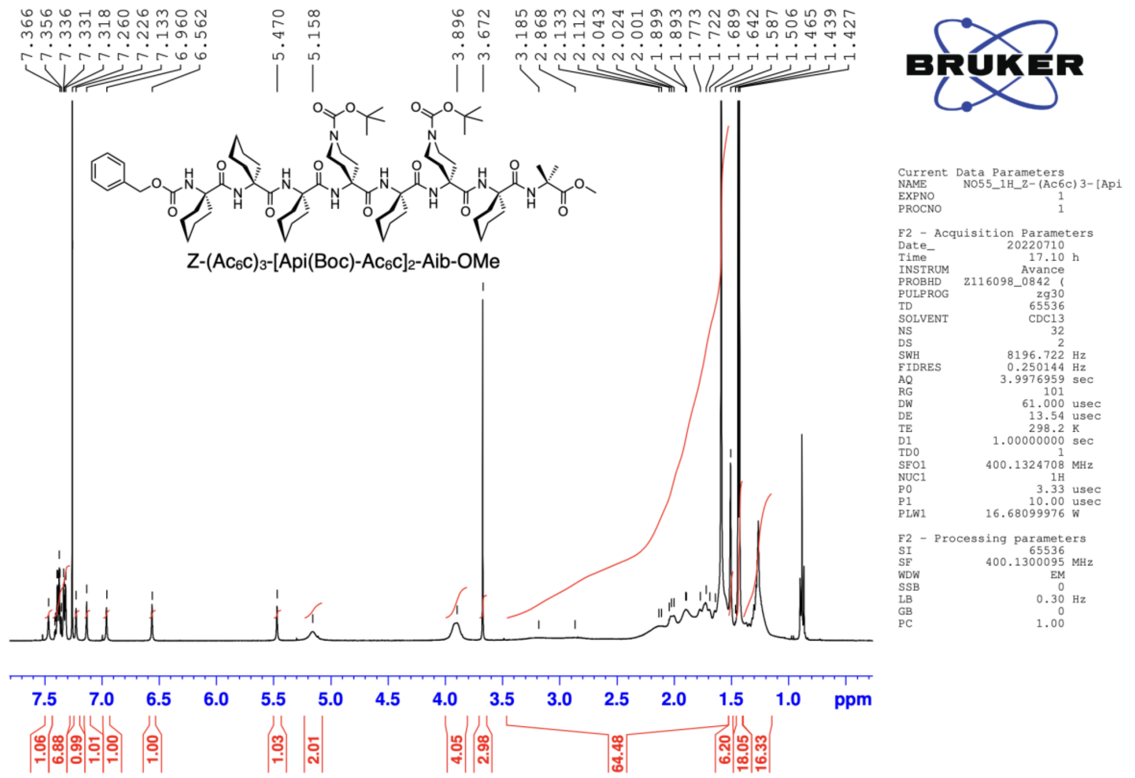

^{1}H NMR (400 MHz, $CDCl_3$, 25 ºC) spectrum of Z-$(Ac_6c)_3$-[Api(Boc)-$Ac_6c]_2$-Aib-OMe.

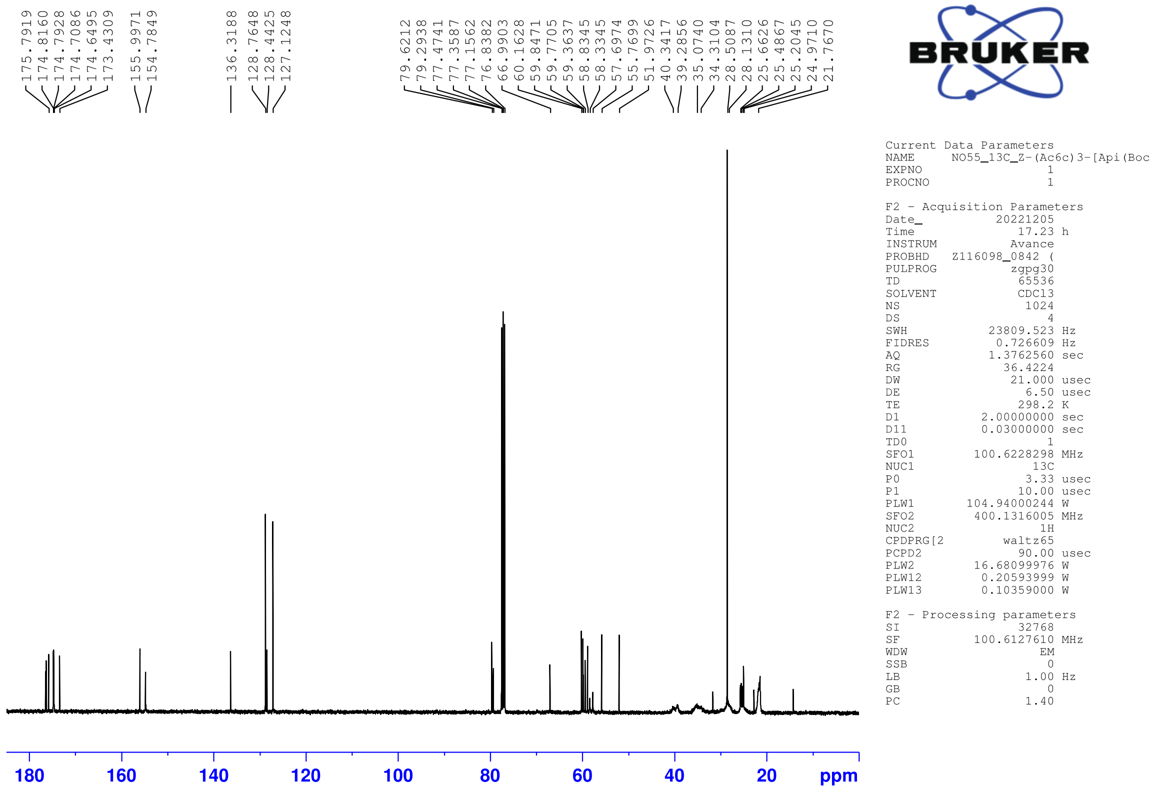

^{13}C NMR (100 MHz, $CDCl_3$, 25 ºC) spectrum of Z-$(Ac_6c)_3$-[Api(Boc)-$Ac_6c]_2$-Aib-OMe.

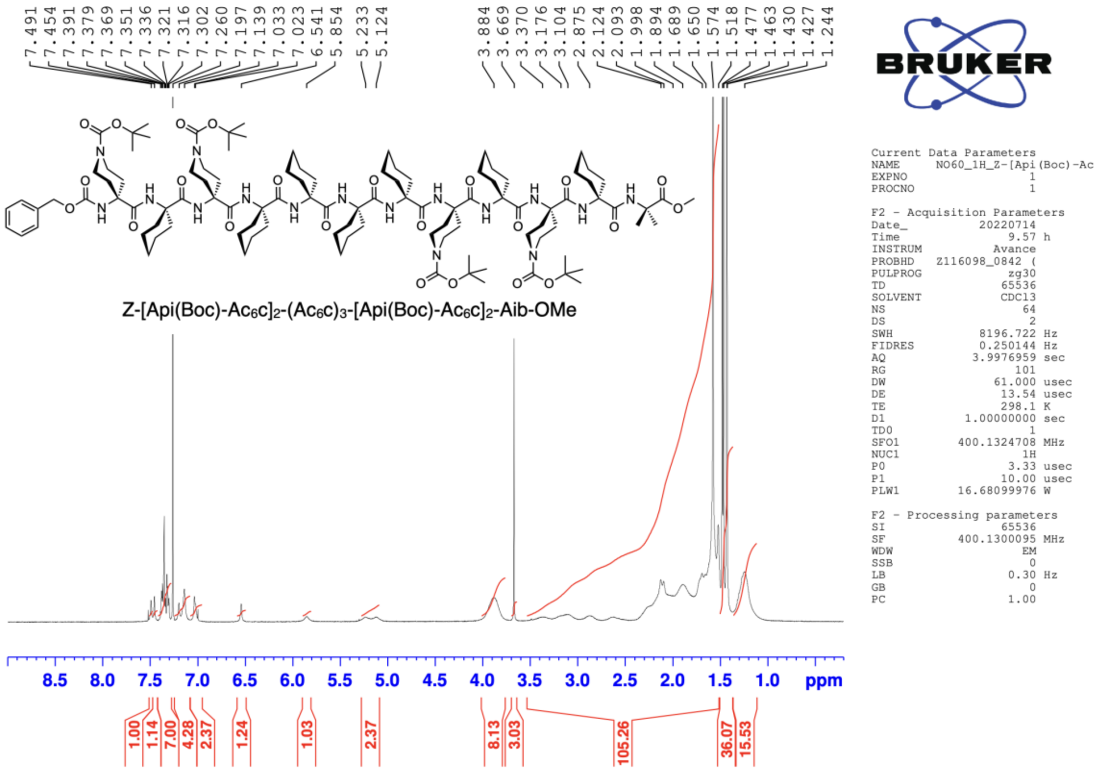

^{1}H NMR (400 MHz, $CDCl_3$, 25 ºC) spectrum of Z-[Api(Boc)-Ac$_6$c]$_2$-(Ac$_6$c)$_3$-[Api(Boc)-Ac$_6$c]$_2$-Aib-OMe.

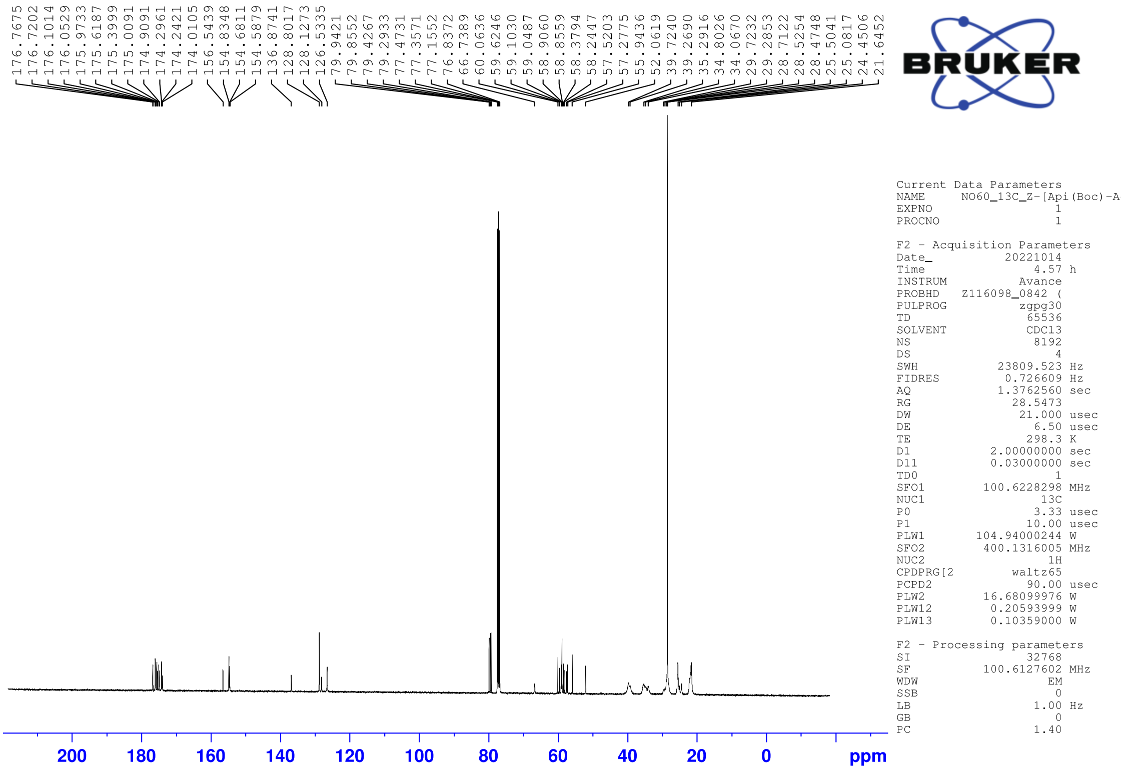

^{13}C NMR (100 MHz, $CDCl_3$, 25 ºC) spectrum of Z-[Api(Boc)-Ac$_6$c]$_2$-(Ac$_6$c)$_3$-[Api(Boc)-Ac$_6$c]$_2$-Aib-OMe.

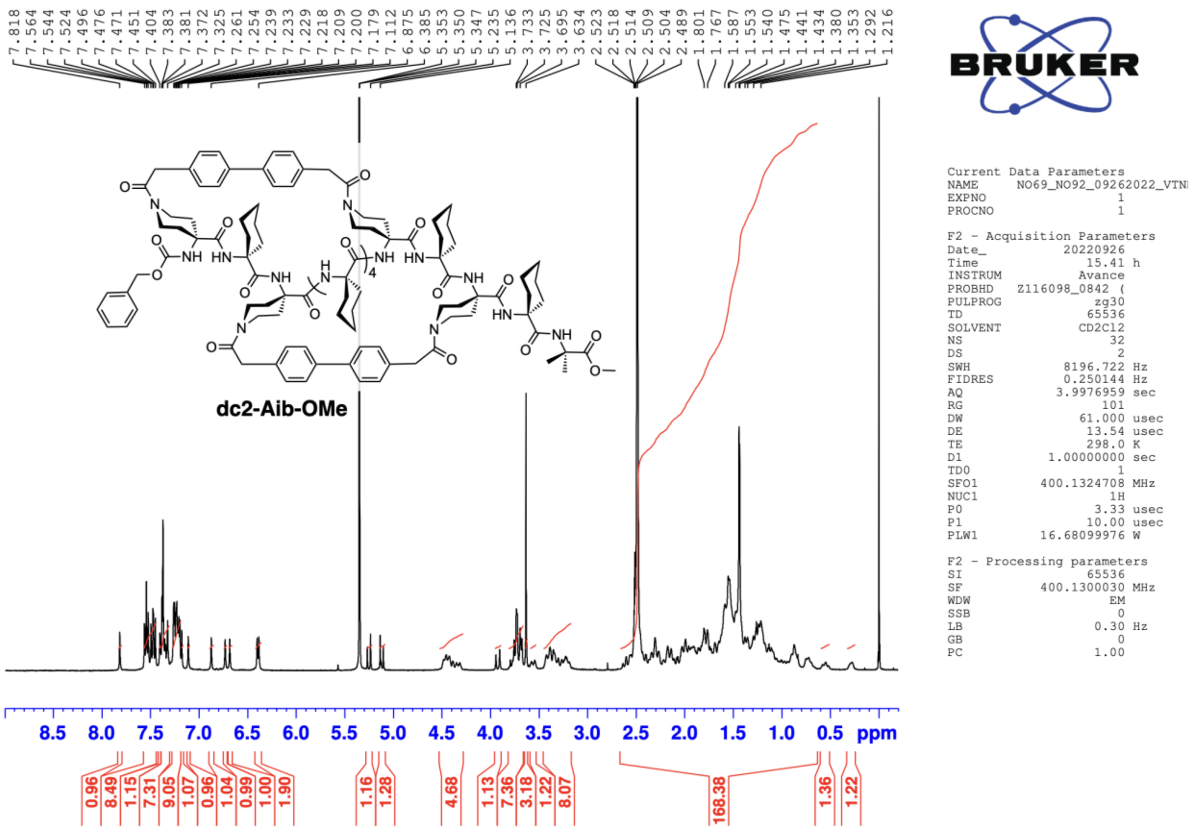

^{1}H NMR (400 MHz, CD_2Cl_2, 25 ºC) spectrum of **dc2-Aib-OMe**.

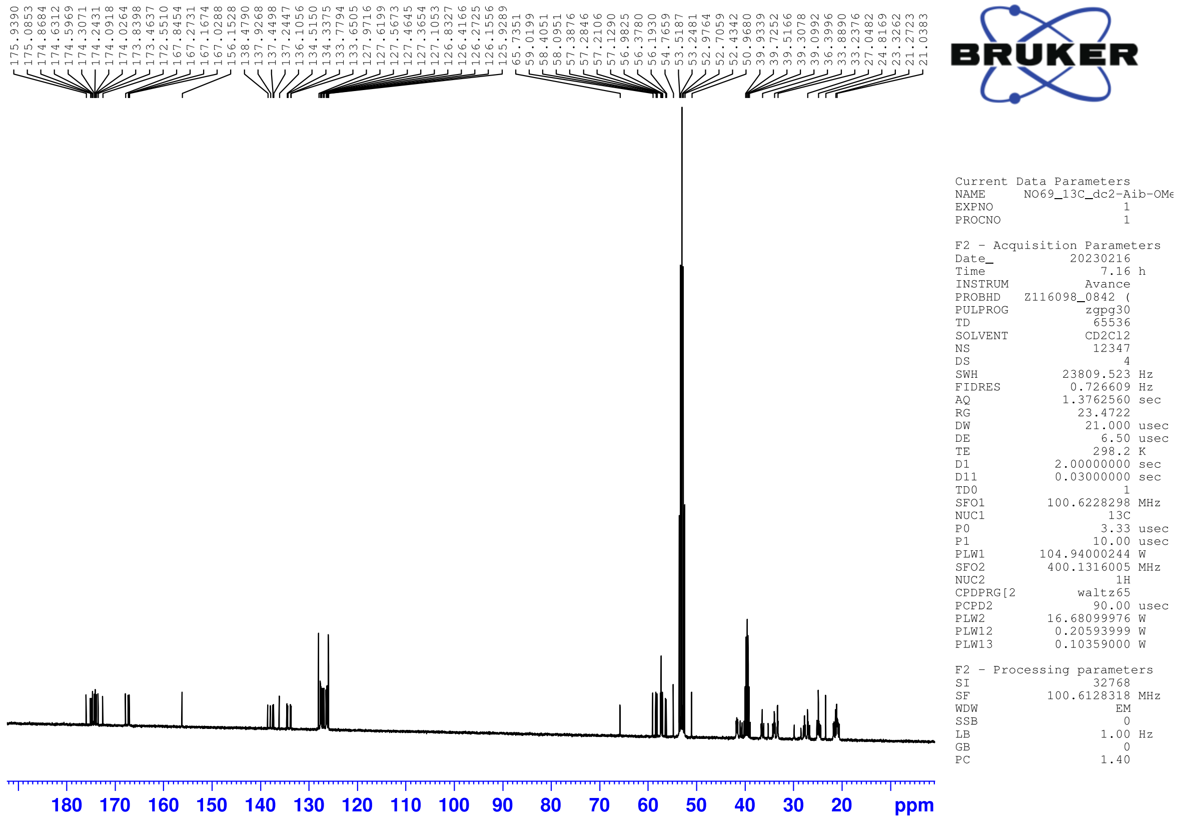

^{13}C NMR (100 MHz, CD_2Cl_2, 25 ºC) spectrum of **dc2-Aib-OMe**.

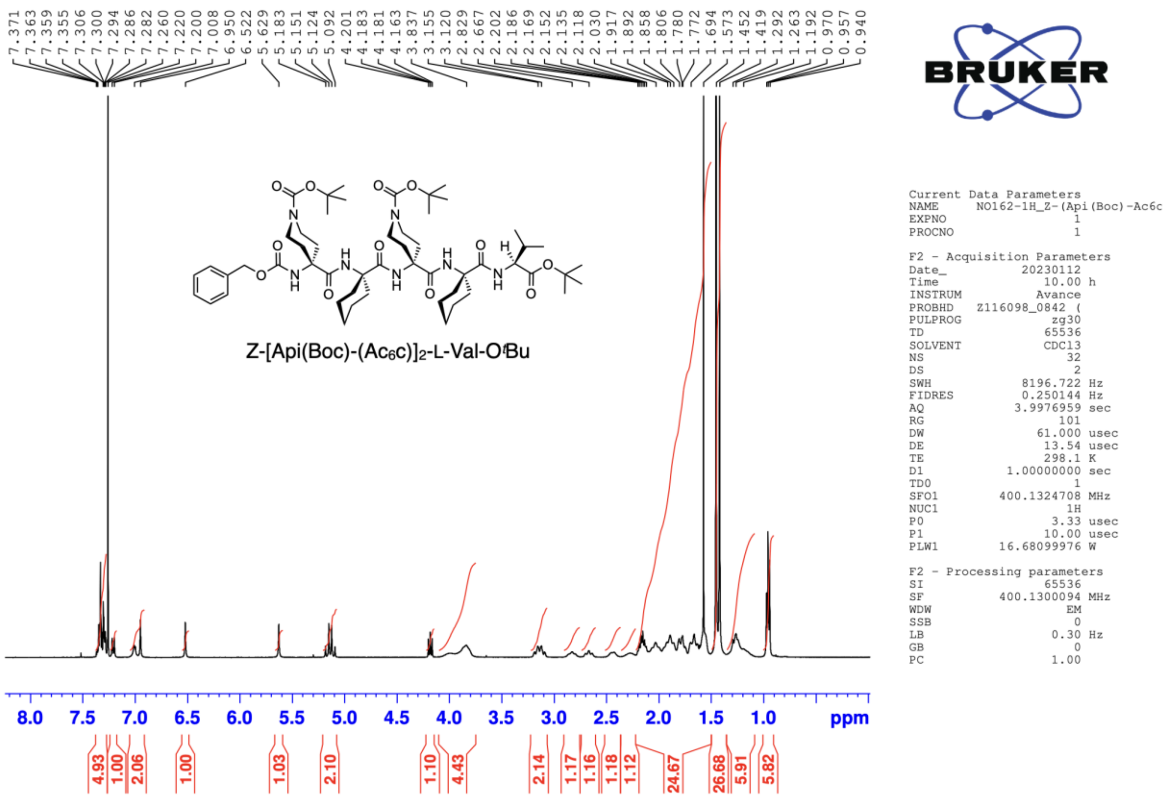

^{1}H NMR (400 MHz, $CDCl_3$, 25 ºC) spectrum of Z-[Api(Boc)-Ac$_6$c]$_2$-L-Val-O^{t}Bu.

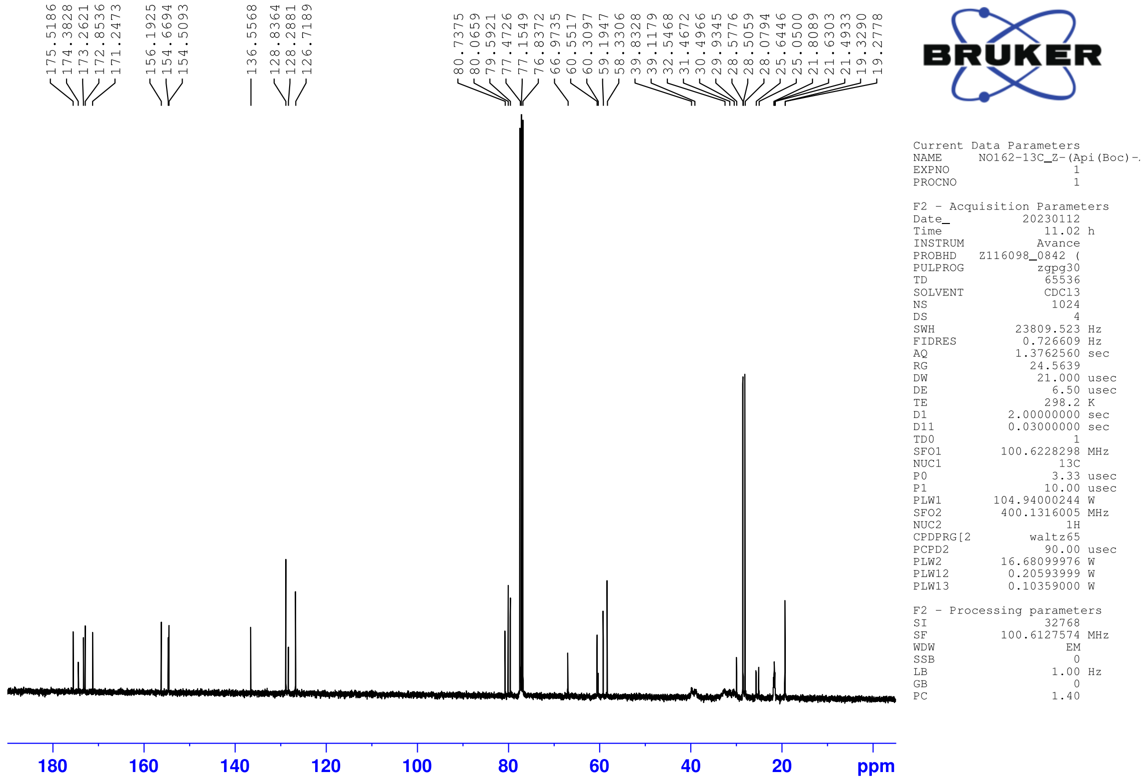

^{13}C NMR (100 MHz, $CDCl_3$, 25 ºC) spectrum of Z-[Api(Boc)-Ac$_6$c]$_2$-L-Val-O^{t}Bu.

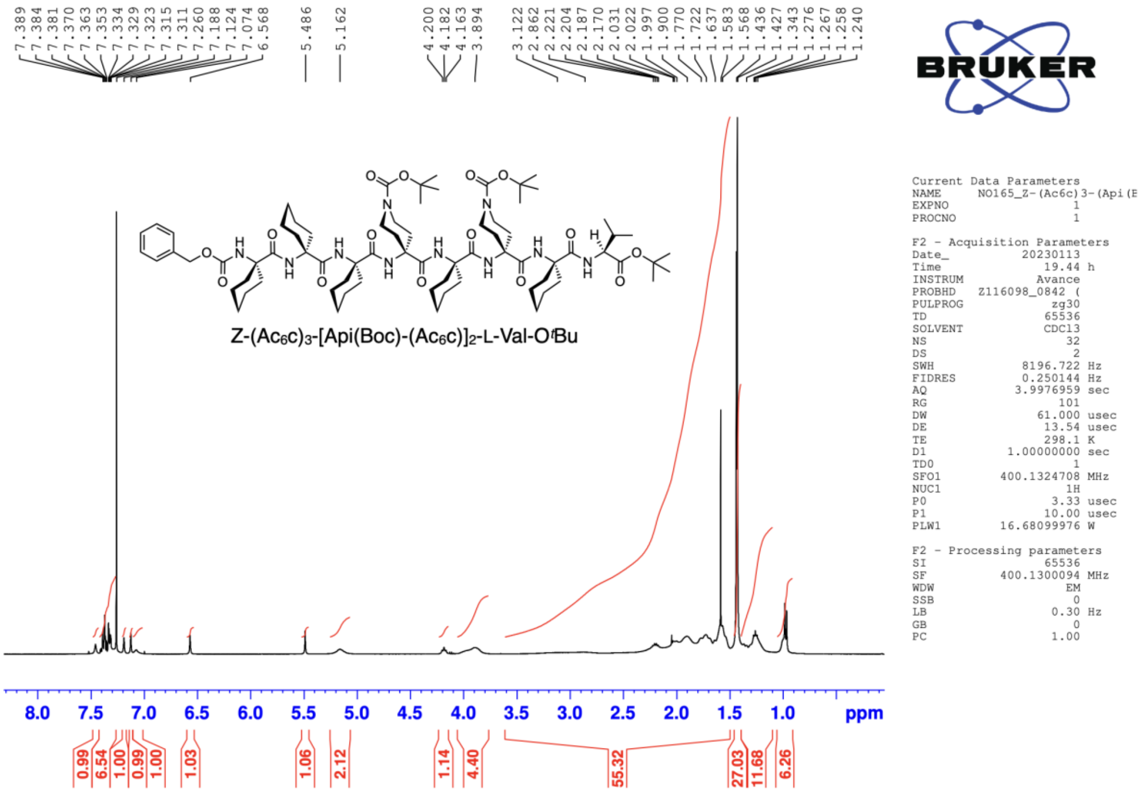

^{1}H NMR (400 MHz, $CDCl_3$, 25 ºC) spectrum of Z-$(Ac_6c)_3$-$[Api(Boc)$-$Ac_6c]_2$-L-Val-O^{t}Bu.

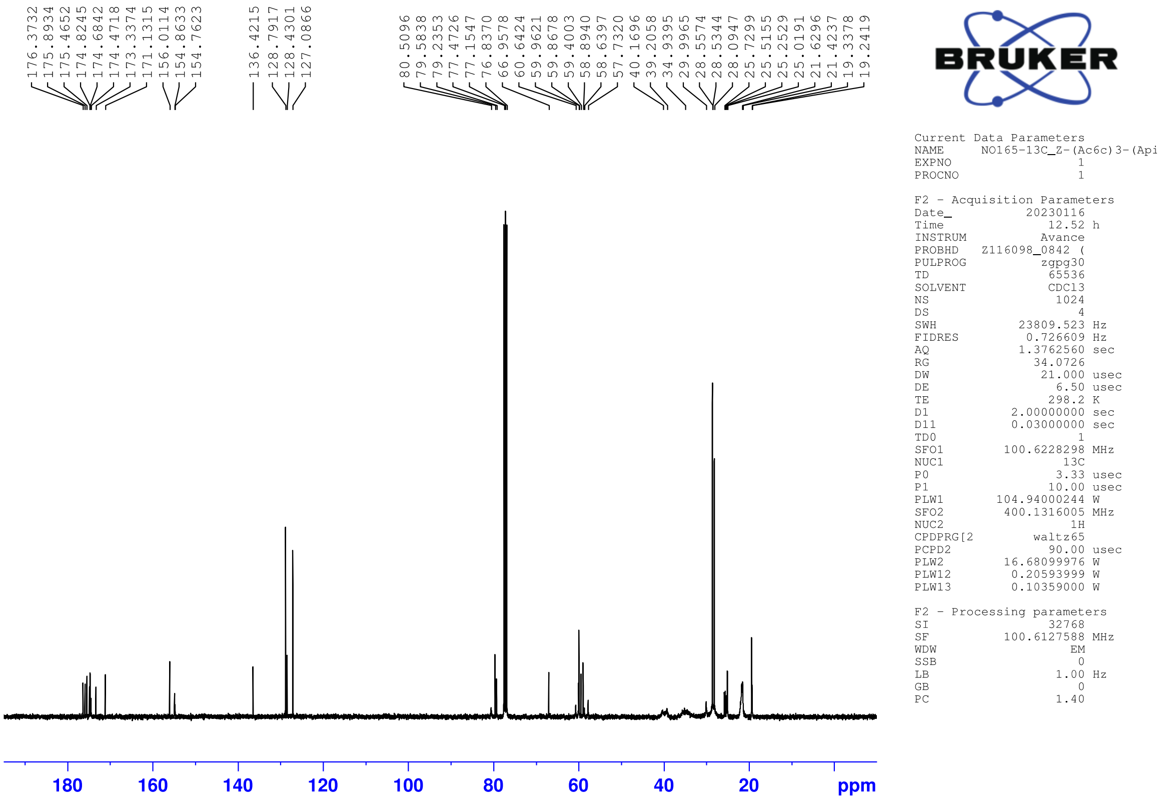

^{13}C NMR (100 MHz, $CDCl_3$, 25 ºC) spectrum of Z-$(Ac_6c)_3$-$[Api(Boc)$-$Ac_6c]_2$-L-Val-O^{t}Bu.

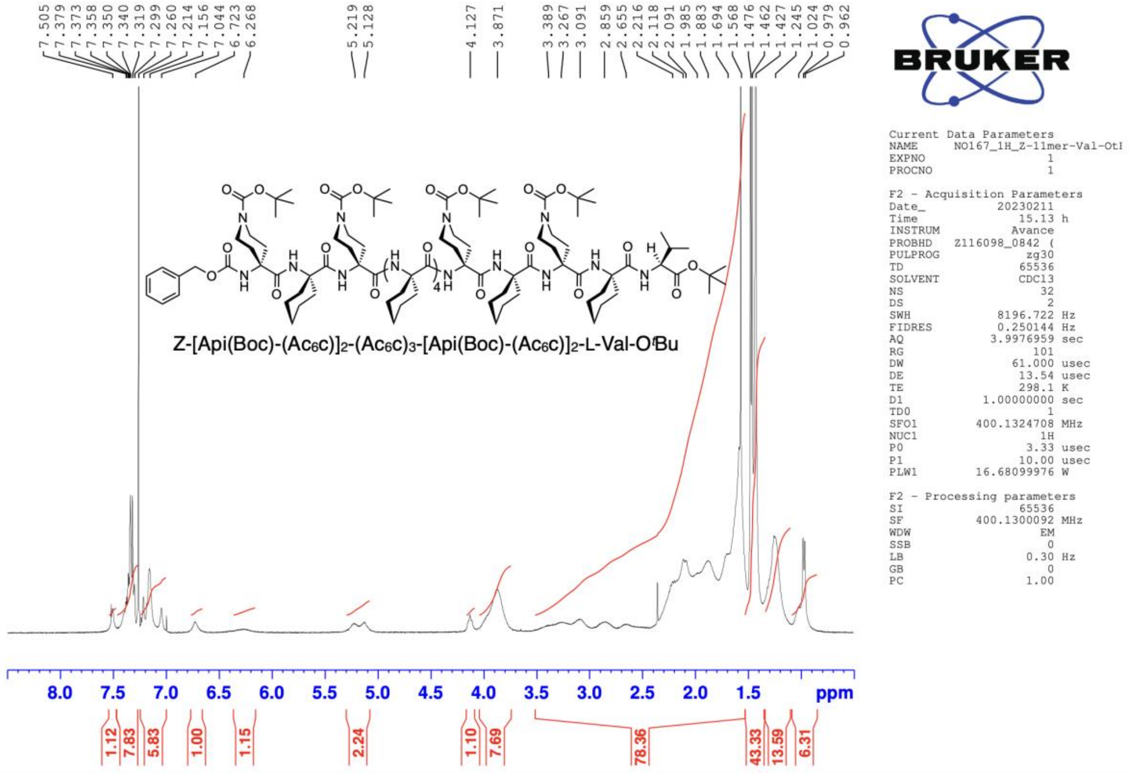

^{1}H NMR (400 MHz, $CDCl_3$, 25 ºC) spectrum of Z-[Api(Boc)-Ac$_6$c]$_2$-(Ac$_6$c)$_3$-[Api(Boc)-Ac$_6$c]$_2$-L-Val-O^{t}Bu.

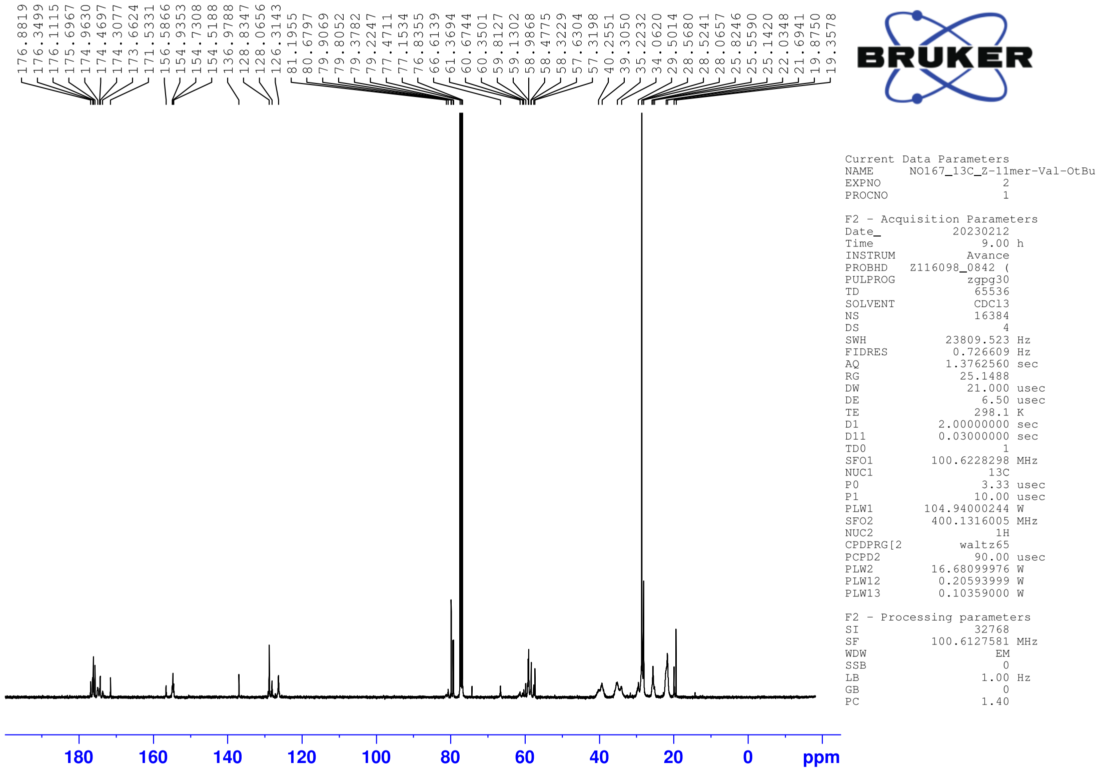

^{13}C NMR (100 MHz, $CDCl_3$, 25 ºC) spectrum of Z-[Api(Boc)-Ac$_6$c]$_2$-(Ac$_6$c)$_3$-[Api(Boc)-Ac$_6$c]$_2$-L-Val-O^{t}Bu.

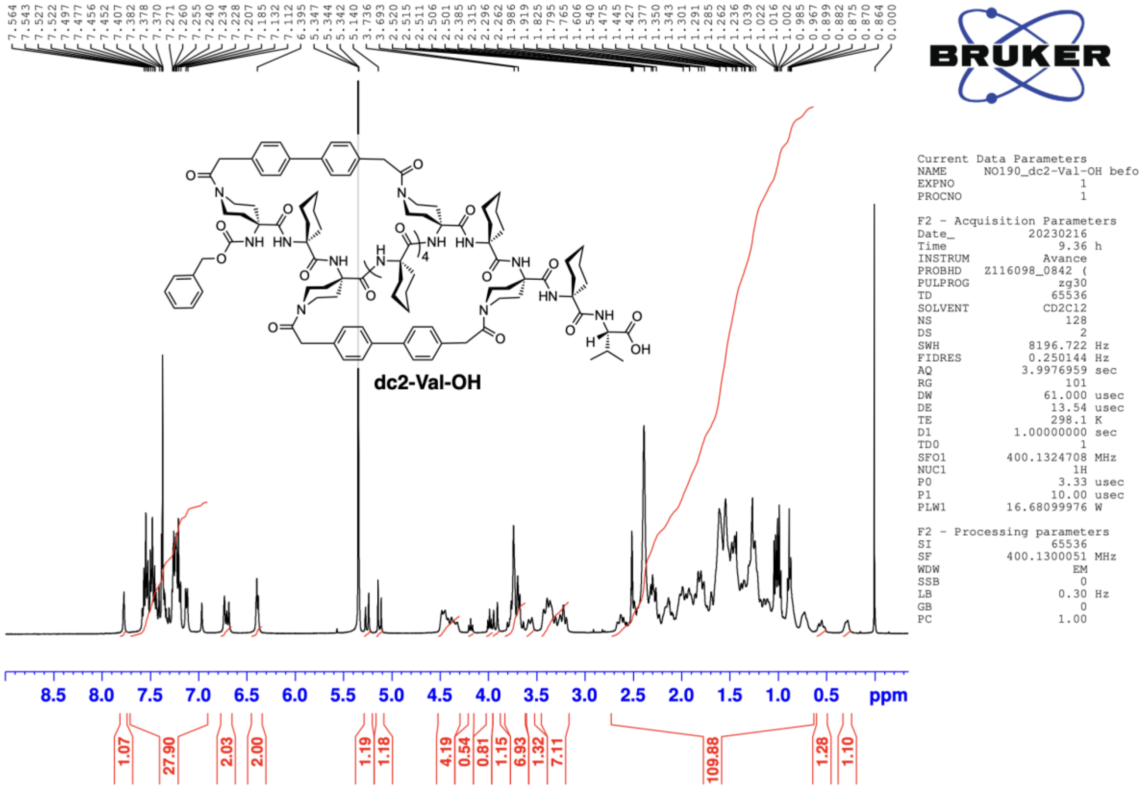

^{1}H NMR (400 MHz, $CD_2Cl_2/(CD_3)_2SO$ (19/1, v/v), 25 ºC) spectrum of the as-obtained **dc2-Val-OH**.

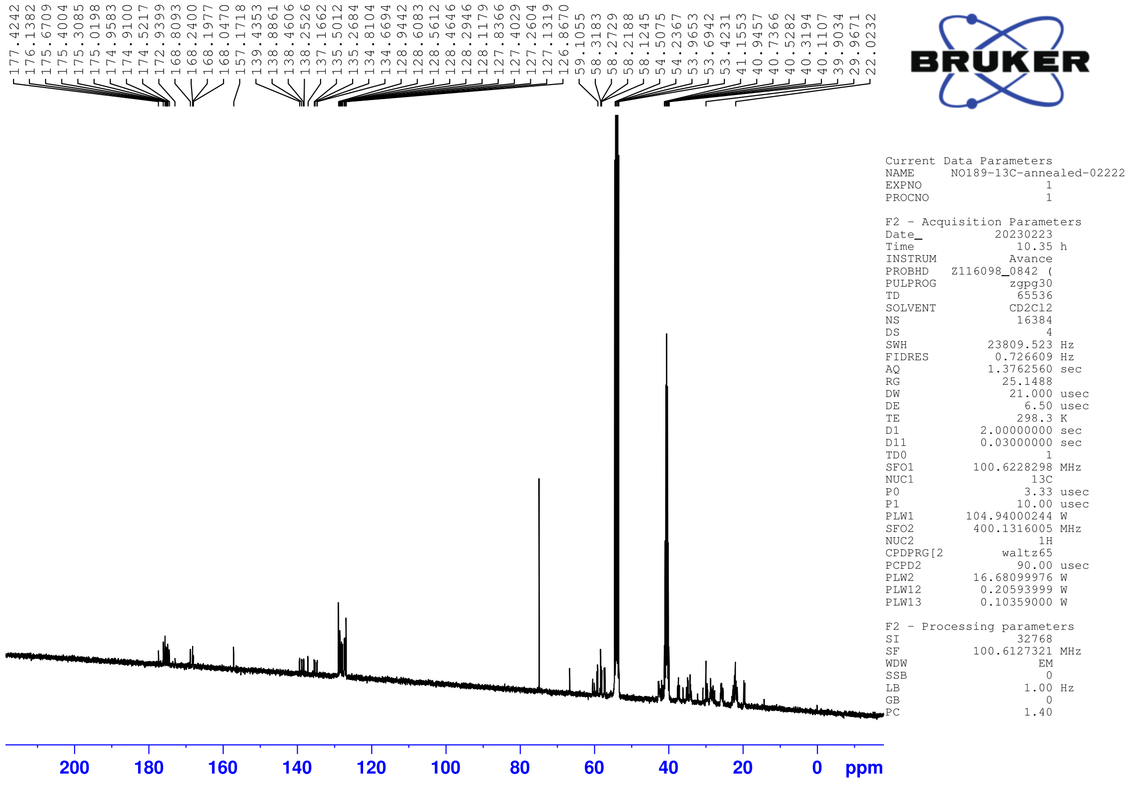

^{13}C NMR (100 MHz, $CD_2Cl_2/(CD_3)_2SO$ (9/1, v/v), 25 ºC) spectrum of the annealed **dc2-Val-OH**.

15. Supporting References

1 Pastuszak, J., Gardner, J. H., Singh, J. & Rich, D. H. Improved synthesis of [4-alanine]chlamydocin: Cyclization studies of tetrapeptides containing five α-substituents. *J. Org. Chem.* **47**, 2982-2987 (1982).

2 Ousaka, N., Sato, T. & Kuroda, R. Intramolecular crosslinking of an optically inactive 3_{10}-helical peptide: Stabilization of structure and helix sense. *J. Am. Chem. Soc.* **130**, 463-465 (2008).

3 Crisma, M. *et al.* Structural versatility of peptides from $C^{\alpha,\alpha}$-dialkylated glycines. An infrared absorption and ^{1}H nuclear magnetic resonance study of homopeptides from 1-aminocyclohexane-1-carboxylic acid. *Macromolecules* **21**, 2071-2074 (1988).

4 El-Faham, A., Funosas, R. S., Prohens, R. & Albericio, F. COMU: A safer and more effective replacement for benzotriazole-based uronium coupling reagents. *Chem. - Eur. J.* **15**, 9404-9416 (2009).

5 El-Faham, A. & Albericio, F. COMU: A third generation of uronium-type coupling reagents. *J. Pep. Sci.* **16**, 6-9 (2010).

6 Thompson, M. ArgusLab, Planaria Software LLC, Seattle, WA. (1996).

7 Arnott, S. & Wonacott, A. J. Atomic co-ordinates for an α-helix: Refinement of the crystal structure of α-poly-L-alanine. *J. Mol. Biol.* **21**, 371-383 (1966).

8 Arnott, S. & Dover, S. D. Refinement of bond angles of an α-helix. *J. Mol. Biol.* **30**, 209-212 (1967).

9 Toniolo, C. & Benedetti, E. The polypepetide-3_{10}-helix. *Trends Biochem. Sci.* **16**, 350-353 (1991).

10 Benedetti, E. *et al.* Helical screw sense of peptide molecules: The pentapeptide system $(Aib)_4$/L-Val[L-(αMe)Val] in the crystal state. *Biopolymers* **46**, 433-443 (1998).

11 Stewart, J. J. P. Optimization of parameters for semiempirical methods V: Modification of NDDO approximations and application to 70 elements. *J. Mol. Model.* **13**, 1173-1213 (2007).

12 Stewart, J. J. P. MOPAC2012, Stewart Computational Chemistry, Colorado Springs, CO, USA, http://openmopac.net/. (2012).

13 Grimme, S., Antony, J., Ehrlich, S. & Krieg, H. A consistent and accurate ab initio parametrization of density functional dispersion correction (DFT-D) for the 94 elements H-Pu. *J. Chem. Phys.* **132**, 154104 (2010).

14 Gaussian 16 Rev. A.03 (Wallingford, CT, 2016).

15 Sheldrick, G. M. *SHELXD-2002*, Program for crystal structure determination, University of Göttingen (2002).

16 Sheldrick, G. M. Crystal structure refinement with SHELXL. *Acta Cryst.* **C71**, 3-8 (2015).
